# The future of cold‐adapted plants in changing climates: *Micranthes* (Saxifragaceae) as a case study

**DOI:** 10.1002/ece3.4242

**Published:** 2018-06-25

**Authors:** Rebecca L. Stubbs, Douglas E. Soltis, Nico Cellinese

**Affiliations:** ^1^ Florida Museum of Natural History University of Florida Gainesville Florida; ^2^ Department of Biology University of Florida Gainesville Florida; ^3^ Genetics Institute University of Florida Gainesville Florida

**Keywords:** Arctic, climate change, ecological niche modeling, *Micranthes*, Saxifragaceae, temperate mountains

## Abstract

Research has shown species undergoing range contractions and/or northward and higher elevational movements as a result of changing climates. Here, we evaluate how the distribution of a group of cold‐adapted plant species with similar evolutionary histories changes in response to warming climates. We selected 29 species of *Micranthes* (Saxifragaceae) representing the mountain and Arctic biomes of the Northern Hemisphere. For this analysis, 24,755 data points were input into ecological niche models to assess both present fundamental niches and predicted future ranges under climate change scenarios. Comparisons were made across the Northern Hemisphere between all cold‐adapted *Micranthes,* including Arctic species, montane species, and species defined as narrow endemics. Under future climate change models, 72% of the species would occupy smaller geographical areas than at present. This loss of habitat is most pronounced in Arctic species in general, but is also prevalent in species restricted to higher elevations in mountains. Additionally, narrowly endemic species restricted to high elevations were more susceptible to habitat loss than those species found at lower elevations. Using a large dataset and modeling habitat suitability at a global scale, our results empirically model the threats to cold‐adapted species as a result of warming climates. Although Arctic and alpine biomes share many underlying climate similarities, such as cold and short growing seasons, our results confirm that species in these climates have varied responses to climate change and that key abiotic variables differ between these two habitats.

## 1 INTRODUCTION

Many cold‐adapted species are expected to suffer range contractions as a result of climate change during the next several decades (Alsos et al., 2009; Parmesan, 2006; Thuiller, Lavorel, Araujo, Sykes, & Prentice, 2005). Loss of suitable habitat could lead to extinction, and therefore, understanding how climate change will influence species extinction rates is critical for informing policy regarding the potential biological costs of high levels of greenhouse gas emissions (Urban, 2015). An important aspect is investigating organisms found in ecosystems that are at upper elevational (mountain) and latitudinal (Arctic) limits because these are areas considered to be particularly sensitive to climate change (Pauli, Gottfried, & Grabherr, 1996). Further, Arctic and mountain regions are environmentally similar in that both have a short growing season and low mean annual temperatures; thus, they provide natural systems for testing how species adapted to these conditions differ in climatic tolerances. Despite these similarities, there are pronounced differences in latitude and spatial distributions that in turn produce varied responses in species occupying these biomes. Latitudinal differences result in varied amounts of solar radiation, day length, and onset and duration of the growing season, while spatial differences are seen in the restricted habitats of isolated temperate mountains versus the continuous landscapes of Arctic tundra closer to the poles. Subsequently, the responses of Arctic and alpine ecosystems to climate change may diverge (Ernakovich et al., 2014). Additionally, due to the limited range of many of the high‐elevation and high‐latitude plants, species indicative of these ecosystems have the potential to act as indicators of climate change for these regions (Gignac, 2001). Therefore, by comparing and contrasting Arctic and alpine ecosystems at a global scale, factors critical for the survival and adaptation of the flora in these areas may be revealed (Abeli, Vamosi, & Orsenigo, 2009).

The Arctic, which has undergone an increase in surface temperature almost three times that of the global average in recent decades, is experiencing immense biotic and abiotic changes (Hinzman et al., 2005; Overpeck, Rind, Lacis, & Healy, 1996; Trenberth et al., 2007). In fact, multiple studies have shown that every Arctic ecosystem shows marked shifts including shrubs extending north into tundra (Chapin, Shaver, Giblin, Nadelhoffer, & Laundre, 1995; Parmesan, 2006; Sturm, Racine, & Tape, 2001) and poleward range shifts for individual species on all continents (Parmesan, 2006). Yet, the ecological consequences of climate change in this region, far exceeding those in temperate and tropical biomes, are comparatively underreported (Post et al., 2009). This is in part due to the Arctic often being regarded as a simple, species‐poor system, when in fact it has been shown that its biotic entities are strongly interconnected and that individual species play pivotal roles in supporting ecosystem functions (Post & Forchhammer, 2008; Walker, Epstein, & Welker, 2008). Finally, the Arctic is an ideal natural system for looking at the effects of climatic change because it has a dramatic and well‐documented history of repeated climate oscillations over a short period of time (Brochmann, Edwards, & Alsos, 2013).

Similar to species found at upper latitudinal limits, organisms found at upper elevational limits also provide important systems for investigating the effects of climate change. Mountains contain around 25% of terrestrial biological diversity, and these regions make up half of the world’s biodiversity hotspots (Stöcklin, 2011). Additionally, mountains play a unique ecological role because many cold‐adapted species reach their southernmost occurrences in midlatitude mountain chains; it has been argued that these montane areas likely harbor important biological diversity (Abeli et al., 2009). Mountain ecosystems have received much attention in global warming research because there is a marked decrease in surface area as elevation increases (Randin et al., 2009), and empirical evidence for upward elevational shifts has been demonstrated in temperate mountain systems for a range of species (Elsen & Tingley, 2015). It is, therefore, widely expected that montane species will, in the absence of broad latitudinal shifts, be left with less habitable area as they approach mountain summits, and subsequently be highly vulnerable to climate change (Elsen & Tingley, 2015).

Taken together, both Arctic and montane ecosystems have strong environmental filters, such as short growing seasons and low average annual temperature, which subsequently allows for only a limited number of well‐adapted lineages to survive (Brochmann et al., 2013). However, Arctic and alpine systems vary in duration and intensity of photoperiods; the start of the growing season occurs earlier in alpine systems, whereas solar radiation is delivered over a full 24‐hr period during summer in the Arctic. Both areas receive different amounts of solar radiation on an annual basis, with total yearly solar radiation decreasing toward the poles. In both biomes, the flora is mainly composed of frost‐resistant perennials, and seedling establishment is rare and slow, but alpine plants are better adapted to summer drought stress than Arctic plants and vegetative reproduction is more common in Arctic than alpine areas (Billings & Mooney, 1968).

Over the past decade, ecological niche models (ENMs) have become common and important techniques for evaluating the consequences of climate change on plant distributions (Randin et al., 2009). ENMs are useful tools for modeling realized niches and future niches. However, predictions from ENMs have many downfalls, including errors introduced from misidentified specimens and inaccurate georeferencing, lack of incorporation of eco‐evolutionary process. Furthermore, ENMs do not model microclimates generated by habitat heterogeneity (Cotto et al., 2017; Ferrarini et al., 2016; Kearney & Porter, 2009; Lozier, Aniello, & Hickerson, 2009). Despite, these limitations, ENMs provide a basis for directing conservation concerns (Ferrarini et al., 2016). There have been many studies employing ENMs at regional scales (i.e., political boundaries), and these include many species with vastly different evolutionary histories, climatic tolerances, and dispersal abilities (Loarie et al., 2008; Randin et al., 2009; Warren, Wright, Seifert, & Shaffer, 2014). Therefore, a particular aspect that needs to be assessed is how closely related species, sharing similar life histories, will respond to climate change. By comparing species with similar evolutionary histories, jointly incorporating their entire range on a global scale, questions fundamental to assessing the effects of climate change can be addressed.

Therefore, we examined three questions regarding the effects of climate change across Arctic and mountain ecosystems using the flowering plant clade *Micranthes* (Saxifragaceae). First, is the amount of suitable area for Arctic and montane plants predicted to change as a result of climate change, and, if so, what is driving these changes? Second, what are the differences in potential or predicted responses to climate change between mountain and Arctic species? Third, what variables affect habitat loss for narrow endemics in these regions? We examine these questions in an evolutionary context, and by investigating within the *Micranthes* clade, rather than between clades, we take into account that many of the factors influencing adaptation and dispersal are likely to share an evolutionary history.

## 2 MATERIALS AND METHODS

### 2.1 Study species

**Figure 1:**
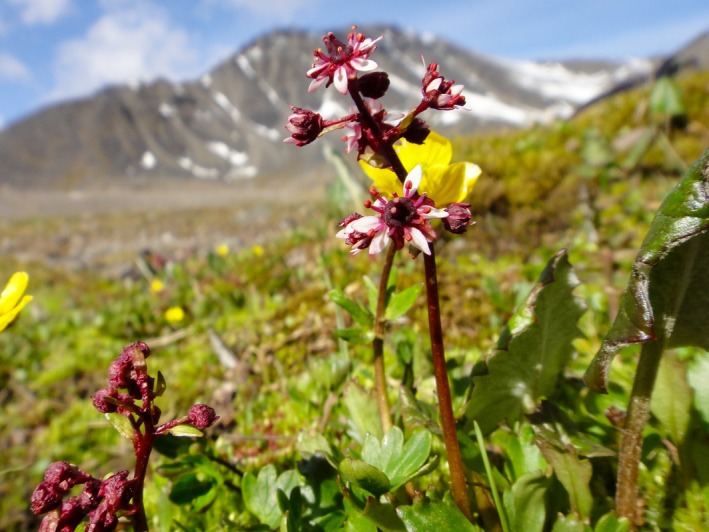
*Micranthes razshivinii* in the Brooks Range near Coldfoot, Alaska

*Micranthes* (Saxifragaceae) (Figure 1), a clade of small‐flowered herbs comprising 75 species, is an ideal group for investigating the possible biotic impact of climate change in montane and Arctic biomes. This is because they have varied spatial distributions, occur in a diversity of habitats, and exhibit a high occupancy of Arctic and alpine areas. *Micranthes* is one of the largest genera in Saxifragaceae with the highest diversity found in North America (Brouillet & Elvander, 2009; Tkach, Röser, & Hoffmann, 2015). Species are found primarily in north temperate, Arctic, and alpine regions and span from sea level in northern latitudes (e.g., *M. nudicaulis* (D. Don) Gornall & H. Ohba) to 5,300 m in the Himalayas (e.g., *M. melanocentra* (Franch.) Losinsk.) (Brouillet & Elvander, 2009; Jintang, Gornall, & Ohba, 2001). Some plants are narrow endemics (e.g., *M. eriophora* (S. Watson) Small) while others are circumpolar (e.g., *M. foliolosa* (R. Brown) Gornall) (Webb & Gornall, 1989). *Micranthes* shows a wide range of ecological diversity with species occurring in diverse locations, including the highest peaks of the Sierra Nevada in California, the subtropical montane forests of China, and the tundra of Alaska. Over one‐third of all species of *Micranthes* are cold‐adapted—in comparison with only four percent of all known vascular plant species (Chapin & Körner, 1994)—suggesting that this group is specialized for these conditions. This is further supported by the fact that many of the cold‐adapted *Micranthes* have a suite of specialized morphological and reproductive traits absent from low‐elevation and low‐latitude species within this clade, including leaf succulence, strongly asymmetric corollas, and asexual reproduction through bulbils. These attributes make *Micranthes* an exemplary group for exploring the evolution and geographic spread of cold‐adapted plants in the context of climate change.

**Figure 2:**
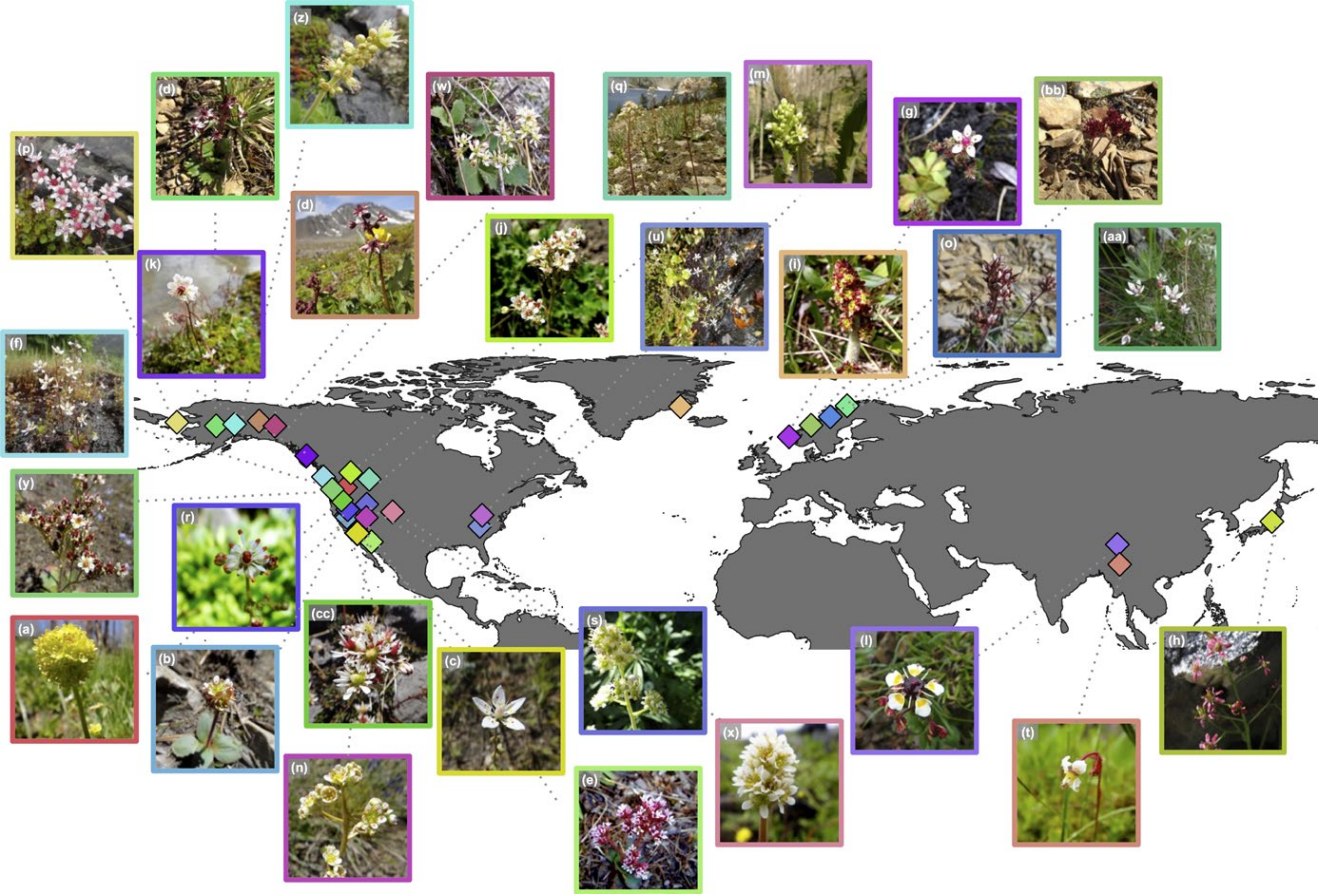
General distribution of the species of *Micranthes* used in this study. All markers are placed at the average latitude and longitude for each species. Letters correspond to species listed in Table 1. All photographs by R.L.S except for (aa) by Thomas Koffel and (h) by Martin Hajman

Specifically, to investigate how cold‐adapted plants will be affected by projected climate change this study focused on species that are present at high latitudes, high elevations, or both. This list was compiled from fieldwork and floras (Brouillet & Elvander, 2009; Webb & Gornall, 1989). We set a minimum threshold of 10 records per species. Further, one species, *Micranthes nelsoniana* (D. Don) Small, was removed from our analyses due to taxonomic confusion at the infraspecific level. Using these filtering criteria, 29 species were used for generating ENMs (Table 1, Figure 2); these species occupy different habitats, latitudes, and elevations, and are dispersed throughout the *Micranthes* clade (Stubbs, Folk, Xiang, Soltis, & Cellinese, 2018; Stubbs, R.L. unpublished).

**Table 1:** Summary of 29 *Micranthes* species

Taxon	Data points	Mean Latitude	Min–Max Latitude	Mean elev. (m)	Min–Max elev. (m)	Biome zone	TTP	AUC	Future/current
(a) *M. apetala*	15	46.573°N	42.684–48.679°N	1,392	87–4,351m	T	0.527	0.947	0.025
(b) *M. aprica*	280	38.608°N	36.284–42.089°N	2,589	1–1,950m	T	0.45	0.976	0.185
(c) *M. bryophora*	284	38.269°N	36.275–41.888°N	2,630	0–3,212m	T	0.413	0.977	0.844
(d) *M. calycina*	66	63.779°N	55.323–69.2661°N	751	1–1,590m	A	0.195	0.962	0.210
(e) *M. eriophora*	14	31.403°N	27.693–32.415°N	2,318	0–2,625m	T	0.08	0.904	0.462
(f) *M. ferruginea*	385	49.556°N	39.063–61.160°N	1,251	979–4,311m	T and A	0.244	0.889	0.256
(g) *M. foliolosa*	1,542	69.407°N	45.904–81.617°N	683	9–1,783m	A	0.364	0.938	1.905
(h) *M. fusca*	45	37.085°N	32.468–44.133°N	1,238	549–2,529m	T	0.143	0.923	0.310
(i) *M. hieraciifolia*	721	69.153°N	42.929–79.980°N	631	531–1,941m	A	0.19	0.95	3.564
(j) *M. idahoensis*	41	46.992°N	43.4683–49.683°N	1,107	838–4,779m	T	0.418	0.959	9.260
(k) *M. lyallii*	205	55.457°N	43.133–63.830°N	1,277	2–4,201m	T and A	0.313	0.929	0.650
(l) *M. melanocentra*	65	30.665°N	26.876–37.006°N	3,727	190–3,996m	T	0.292	0.919	0.629
(m) *M. micranthidifolia*	32	36.570°N	35.133–40.574°N	800	87–3,481m	T	0.378	0.964	1.574
(n) *M. nidifica*	294	41.887°N	35.965–50.666°N	1,692	3–636m	T	0.301	0.918	1.607
(o) *M. nivalis*	4,135	65.452°N	45.554–81.820°N	675	1–2,278m	T and A	0.195	0.677	0.676
(p) *M. nudicaulis*	18	64.796°N	61.730–65.696°N	270	23–3,750m	A	0.173	0.94	0.206
(q) *M. occidentalis*	139	48.881°N	40.591–59.416°N	1,667	160–1,553m	T	0.303	0.941	5.173
(r) *M. odontoloma*	497	40.434°N	34.117–53.099°N	2,410	1,524–5,548m	T	0.317	0.931	1.066
(s) *M. oregana*	481	40.389°N	35.799–48.672°N	1,900	7–2,731m	T	0.413	0.936	0.442
(t) *M. pallida*	37	28.111°N	23.763–34.670°N	3,152	175–3,590m	T	0.258	0.926	0.744
(u) *M. petiolaris*	39	35.741°N	35.008–36.650°N	1,600	1–2,297m	T	0.466	0.985	0.282
(v) *M. razshivinii*	60	65.143°N	60.966–69.433°N	1,304	0–2,647m	A	0.355	0.931	0.255
(w) *M. reflexa*	35	63.755°N	59.883–70.002°N	842	1–1,828m	A	0.221	0.846	0.395
(x) *M. rhomboidea*	160	39.775°N	36.850–52.858°N	3,090	4–2,820m	T	0.456	0.975	0.398
(y) *M. rufidula*	85	46.048°N	41.413–50.050°N	746	5–2,744m	T	0.21	0.94	1.925
(z) *M. spicata*	58	64.236°N	59.830–68.180°N	635	15–1,764m	A	0.24	0.86	0.258
(aa) *M. stellaris*	12,468	60.717°N	36.998–71.064°N	775	580–4,036m	T and A	0.28	0.67	0.625
(bb) *M. tenuis*	2,274	66.321°N	48.883–81.816°N	762	103–3,911m	T and A	0.191	0.768	0.518
(cc) *M. tolmiei*	280	43.101°N	36.399–58.284°N	2,338	582–2,457m	T	0.36	0.961	0.271

*Note*

T = temperate mountains, A = Arctic. TTP = 10% training presence threshold. AUC = area under the cover. Future/Current = the future area divided by the current area, numbers >1 represent an increase in future niche size.

### 2.2 Occurrence data

Georeferenced occurrence data were downloaded from the Global Biodiversity Information Facility (gibif.org, November 2017; available from https://doi.org/10.5061/dryad.7294nh2) using the “gbif” function in the R library “dismo.” All duplicate specimens were removed, and specimen data were further cleaned to only include specimens that had coordinates with two or more decimal places. This minimum requirement was established because locality specificity to two decimal places equates to 1,110 m (at the equator) and is therefore sufficient for running analyses with 30 arc second spatial resolution in environmental layers (Heap & Culham, 2010). *Micranthes* are restricted to the Northern Hemisphere, so erroneous occurrence points falling in the Southern Hemisphere and/or not on a land mass were removed using custom scripts in R. All occurrence data were visually examined in QGIS. Any locality points falling well outside the accepted known range (e.g., species endemic to Alaska with points in Central America) were removed. To estimate the geographical range and for delimiting the modeling area for each species, we placed a buffer around each location using the “gBuffer” function of “rgeos” library. The range buffer was set to “2” creating a square buffer approximately 170 km in any direction from each point. These custom shapefiles for each species were manually reviewed and edited to be continuous areas for the final shapefile.

### 2.3 Climate and soil data

We considered an initial set of 26 environmental variables representing various candidate predictors that are potentially relevant for the distribution of *Micranthes*. This included all Bioclim variables at 30 arc second resolution, downloaded from the Worldclim Global Climate Data website (worldclim.org; Hijmans, Cameron, Parra, Jones, & Jarvis, 2005); elevation; and bulk density, clay content, silt content, sand content, soil organic carbon content, and soil pH, downloaded from SoilGrids website (soilgrids.org, Hengl et al., 2017). Soil layers were downloaded in 2.5 arc minute resolutions; these were projected into 30 arc second resolutions using the “resample” function of the “raster” library in R. Climatic data for the projected time period 2061–2080 at RCP8.5 were also obtained from Worldclim. The RCP8.5 incorporates large populations, moderate rates of technological change, and an absence of climate change policies resulting in high‐energy demand and greenhouse gas emissions (Riahi et al., 2011). Each layer was cropped to each species’ custom shapefile, including buffered area, representing its geographic range and potentially habitable space.

### 2.4 Accounting for sampling bias

Sampling bias results from more species being collected from relatively accessible locations (i.e., near roads, urban areas, and bodies of water), so the resulting sampled localities are not representative of the actual range of environmental conditions in which each species occurs (Syfert, Smith, & Coomes, 2013). While this is the case for many species of *Micranthes*, some do not occur in easily accessible areas, and are therefore, not likely to be subject to this sampling bias. For this reason, we analyzed all species twice: both with and without sampling bias taken into account. For the analyses that accounted for sampling bias, we generated background data that represented survey effort for similar species across the targeted area (Kramer‐Schadt et al., 2013; Phillips et al., 2009; Syfert et al., 2013). It has been shown that using a target‐group background some sampling bias is removed by spreading predictions into unsampled areas with similar environmental conditions (Phillips et al., 2009). To create files that have background data with a similar bias as our datasets we chose to use all occurrences for Saxifragaceae. This set of data points likely represents similar collections efforts as *Micranthes*. These occurrence points were cleaned and filtered using the same methods as used for the *Micranthes* occurrence data (above) and resulted in 164,316 records. The remaining data points were converted into a raster file with the same resolution (30 arc seconds) as the climate and soil data. A bias grid was derived using a two‐dimensional kernel density estimation that down‐weights points smoothly as the distance from each occurrence point increases. This file was generated using the “kde2d” function in the R library “MASS.” In this bias file, the cell values reflect sampling effort and weight random background data for modeling (Fourcade, Engler, Rödder, & Secondi, 2014). The bias file was individually cropped to each of the custom shapefiles used to represent the distribution of each of our focal species. The resulting file was converted to a raster and used as the input biasfile in MaxEnt.

### 2.5 Ecological niche models

Habitat suitability for *Micranthes* under present and future environmental conditions was estimated using ENMs. To avoid over fitting the models, a cutoff of 0.8 Pearson’s correlation coefficient was imposed for both the climate and soil layers, which reduced the number of layers for subsequent analyses (Supporting Information Appendix S1). When multiple variables were highly correlated only one was retained, and although this varied by species, an attempt was made to keep layers Bio1 (annual mean temperature) and Bio12 (annual precipitation) for consistency between datasets. Environmental conditions in the distributional range of each species were captured using 10,000 randomly selected background sites. The same shapefile for each species was used to mask the area for both generating and projecting the model. Niche models were generated using the logistic output from MaxEnt v3.3.3k. Default settings were used, with the exception of using 20% random test percentage, 15 subsampled replicates, and 5,000 maximum iterations. Therefore, model calibration was performed on a random sample of the data (80%), and model evaluation was carried out on the remaining 20% using the area under the curve (AUC) statistic as averaged across all replicates. For each species, calibrated models were then used to project current and future suitable climatic habitats.

### 2.6 Area of suitable habitat

Changes in suitable habitat for each species were evaluated by comparing the geographical ranges predicted under both present and future conditions. To aid in model validation and to check for robustness of results, we compared three commonly used MaxEnt thresholds to define the percentage of suitable habitat: 10% training presence (TTP), minimum training presence (MTP), and the maximum training sensitivity plus specificity (MSS) (Supporting Information Appendix S2). These thresholds were applied and compared to define the probability of suitable habitat.

Species were classified as being narrow endemics if their current geographical niche was less than 120,000 km^2^. This cutoff was selected to align with known distributions and supported by the literature, as certain species (e.g., *M. eriophora, M. aprica* (Greene) Small) are regarded as endemics (Brouillet & Elvander, 2009; McGregor, 2008; Webb & Gornall, 1989). By this standard, 11 species were classified as narrow endemics with relatively small geographical niches (<120,000 km^2^).

Niche overlap between the present and future areas of suitable habitat was calculated using Schoener’s *D*, where 0 equals no similarity and 1 equals complete similarity (Broennimann et al., 2012). Further, we compared changes in area (km^2^) of suitable habitat. The area of each cell of the output MaxEnt raster that was considered suitable habitat was summed using the “area” and “zonal” functions of the R library “raster.” This was divided into five categories: total suitable area in the present, total suitable area in the future, suitable area only in the present, suitable area only in the future, and overlapping suitable area in the present and future. To quantify this change by species, we divided the total area of suitable habitat in the future by the total area of suitable habitat in the present; this ratio quantifies how much habitat each species is gaining or losing under this climate change scenario. Species were then classified as either gaining habitat (ratio >1) or losing habitat (ratio <1).

Although we are predicting the future possible distribution of our focal species, we are unable to predict the ability of these species to disperse and establish in new habitat. Therefore, we also wanted to consider how much of the current habitat will remain suitable, despite predicted climate change. For this reason, we quantified and compared how much of the current distribution overlaps with the future projected distribution. This area of overlap represents habitat that will remain suitable from the present into the future and therefore does not require plants to disperse to and colonize new habitat.

### 2.7 Statistical analysis

For our analyses, we ran tests on four groups: all, Arctic, montane, and narrowly endemic species. Data on latitude, elevation, soil pH, silt content, annual mean temperature, and annual precipitation were extracted from the layers used in our models for each of our accessions. These six variables are not significantly correlated (Supporting Information Appendix S3). We used principal component analysis (PCA) and multiple analysis of variance (MANOVA) to address which of six variables influenced the species in our study and to assess whether there was a statistically significant difference between species that lose or gain habitat under future climate models. There were several steps in this process. (a) The output from MaxEnt for all species was divided into the four datasets composed of different subgroups of species (i.e., all, Arctic, mountain, and endemic species). (b) For each group, we reduced the multivariate dataset to uncorrelated sets of variables using PCA based on the correlation matrices. For downstream analyses, we retained the principal components (PC) that had an eigenvalue greater than 1. (c) Using the R package “factoextra”, the PC score for each accession was obtained for every component. (d) To determine whether separation in environmental space was statistically significant between cold‐adapted species that are either positively or negatively impacted by climate change, we followed each PCA with a MANOVA in which species were divided into two groups. Species were classified as either having an increasing or a decreasing niche size as a result of climate change. This binary classification was the fixed factor, and PCA scores were the dependent variables. (e) Plots were constructed using the R package “ggplot2” (Wickham, H. 2016).

## 3 RESULTS

### 3.1 Occurrence data

A total of 22,318 occurrence records were compiled (Table 1). The number of occurrence points per species ranged from 14 to 12,468, and species are distributed throughout the Northern Hemisphere (Figure 2). All occurrence data are available for download in Dryad repository (https://doi.org/10.5061/dryad.7294nh2). The species were distributed between biomes, with seven occurring only in the Arctic, 17 occurring only in temperate regions, and five occurring in both temperate and Arctic areas. For elevation, averages ranged from 270 m (*M. nudicaulis* to 3,727 m (*M. melanocentra* (Franch.) Losinsk.) (Table 1).

### 3.2 Species distribution models

Initially, all analyses were run on two datasets: one corrected for sampling bias and one that was not corrected for sampling bias. As we were interested in the effect this bias had on ENMs, our results will address how these datasets differed, but the discussion is focused only on the results from the dataset that accounted for sampling bias (discussed in detail below). In both the dataset that accounted for sampling bias and the dataset that did not, the majority of species had adequate predictive performance (or AUC > 0.7). In the dataset that did not account for sampling bias, all species had AUC values above 0.7 (Supporting Information Appendix S4). The median training AUC was 0.86 ± 0.13. For the dataset that did account for sampling bias, two species had AUC values below 0.7: *M. stellaris* (0.673) and *M. nivalis* (0.677) (Table 1). The median training AUC from the remaining 27 species was 0.88 ± 0.11. These AUC scores should be interpreted cautiously. Sampling bias can result in spatial clustering of points and affects model quality by inflating model accuracy (higher AUC scores; Veloz, 2009). Conversely, but equally problematic, when incorporating spatial sampling bias into MaxEnt, AUC scores will be lower on both training and test data when presence‐only data are used for model validation, even though the model is in fact a better representation of the species distribution (Phillips et al., 2009).

### 3.3 Accounting for sampling bias

For all but two species, the AUC scores for the dataset that accounted for sampling bias were lower. A much larger difference than noted above was between the two datasets in the amount of suitable habitat predicted by the model under both present and future climate models. The difference in area varied by species, and this difference was not consistently greater or lesser in the models that corrected for sampling bias and the models that did not. Under the present climate conditions, the difference in suitable area between the models that accounted for sampling bias and the uncorrected models varied greatly (Table 1, Supporting Information Appendix S4).

### 3.4 Changes in habitat suitability

The results from using the TTP and MSS thresholds were identical in terms of species increasing or decreasing in geographical area as a result of climate change (Supporting Information Appendix S2). When using the MTP threshold, 26 of the 29 species were classified as being the same, but *M. odontoloma* (Piper) A. Heller*, M. bryophora* (A. Gray) Brouillet & Gornall, and *M. rhomboidea* (Greene) Small had opposite results to the other two thresholding methods (Supporting Information Appendix S3). Because the results from the TTP and MSS were in accordance, and the TTP is a less restrictive threshold value, this threshold was selected to determine suitable habitat.

**Figure 3:**
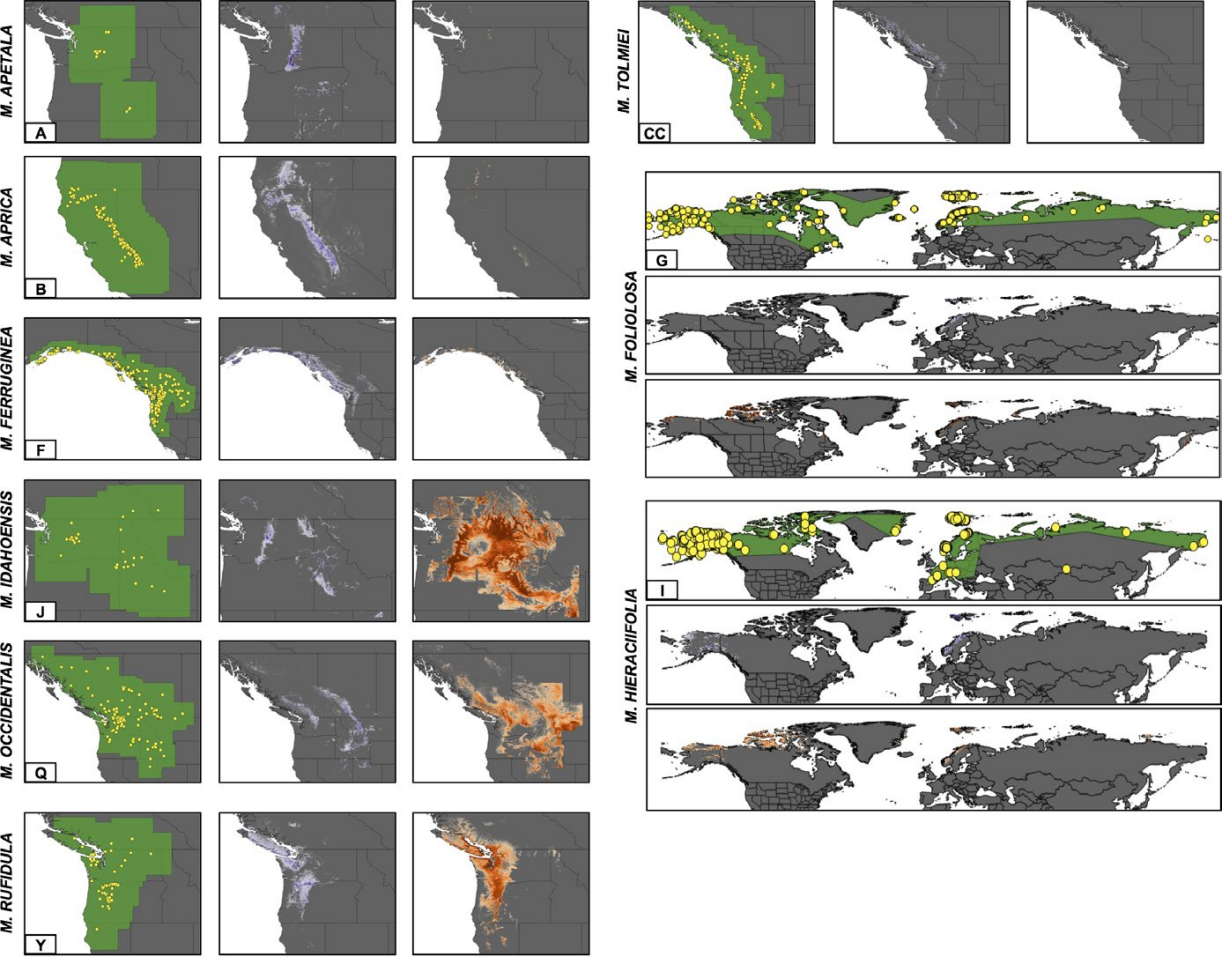
Ecological niche models for *Micranthes* species discussed in the text. All models corrected for sampling models and models for all species are in Supporting Information Figure S1. From left to right, in the first panel, green areas represent the original shapefiles that were used to trim layers. They represent all current and potential habitat. Yellow dots are accessions used to train and test the models. In the second panel, purple areas are designating current geographical area. In the third panel, orange areas are designating future predicted geographical area. In the second and third panels, darker shades represent more suitable areas. Suitable areas designated by the TTP threshold. All areas are in the Northern Hemisphere. Letters correspond to species listed in Table 1 and images are alphabetical, except for species with larger distributions: (g) *M. foliolosa*, (i) *M. hieraciifolia*

Overall, 21 species lose habitat and eight species gain habitat as a result of projected climate change. One species, *M. apetala* (Piper) Small (Figure 3a), is predicted to undergo a decrease in suitable habitat by 97% between its present and future distribution. Two species, *M. occidentalis* (S. Watson) Small and *M. idahoensis* (Piper) Brouillet & Gornall (Figure 3q,j; Supporting Information Figure S1), have the largest increases (4 to 8 times greater) in suitable habitat. Additionally, *M. heraciifolia* (Waldst. & Kit. Ex Willd.) Haw. (Figure 3i; Supporting Information Figure S1) has a twofold increase in suitable area under climate change models.

**Figure 4:**
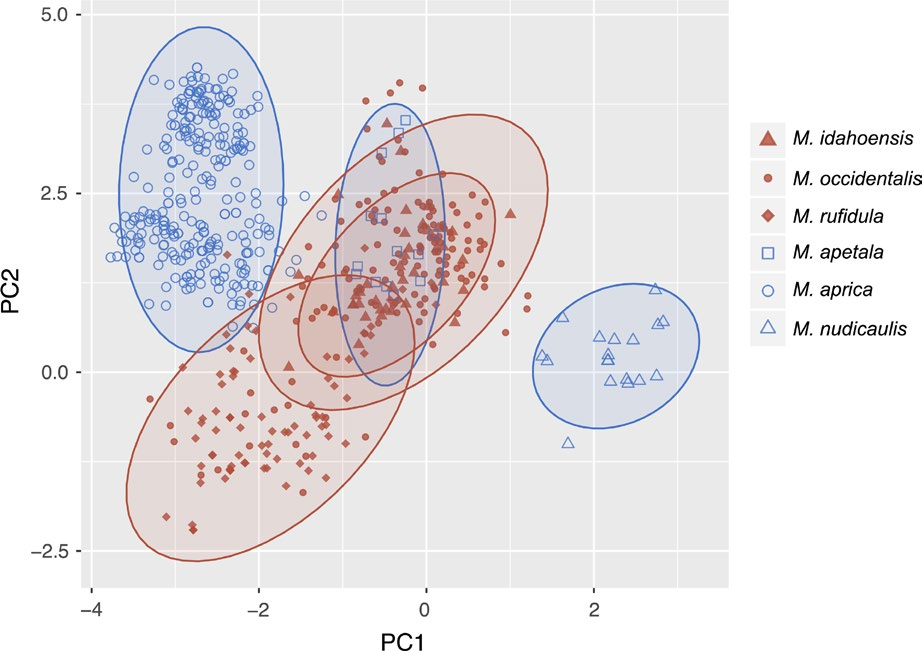
Plot of principal component 1 versus principal component 2 for the PCA performed on all *Micranthes* species. Simplified to show the three species that are predicted to lose the most habitat (blue) and the three species that are predicted to gain the most habitat (red) are shown. Ellipses show the 95% confidence interval for each species. A complete PCA is shown in Supporting Information Figure S2. Statistically significant separation among species that gain and lose habitat occurs along both PCA axis (overall: *F*_1,24723_ = 1726.7, *p* < 0.001; *x*‐axis: *F*_1,24723_ = 960.8, *p* < 0.001; *y*‐axis *F*_1,24723_ = 2301.5, *p* < 0.001). The *x*‐axis explains 39.8% of the variation and the *y*‐axis explains 25.7%

The results of the PCA and MANOVA for all groups and all species are summarized in Supporting Information Appendix S5. The PCA for all cold‐adapted *Micranthes* (Figure 4; complete PCA in Supporting Information Figure S2) suggested divergence in environmental space between all species based on the six variables in our analysis. The PCA was run on all species, but a simplified PCA showing only the three taxa that gain the most habitat and the three taxa that lose the most habitat is provided (Figure 4). The first principal component (PC1) was weighted most heavily on latitude, while the second principal component (PC2) was dominated by elevation and soil pH. The signs of these variable loads are all positive, and these two components explain 65% of the variance (Supporting Information Appendix S5). The MANOVA demonstrated that these components are able to differentiate between species that gain habitat versus those that lose habitat as a result of climate change (*p *<* *0.001). The three species that lose the most habitat are clustered in extremes of the PCA, with one species, *M. nudicaulis* found on the far right of the plot, while the two others are located high on the *y*‐axis.

**Figure 5:**
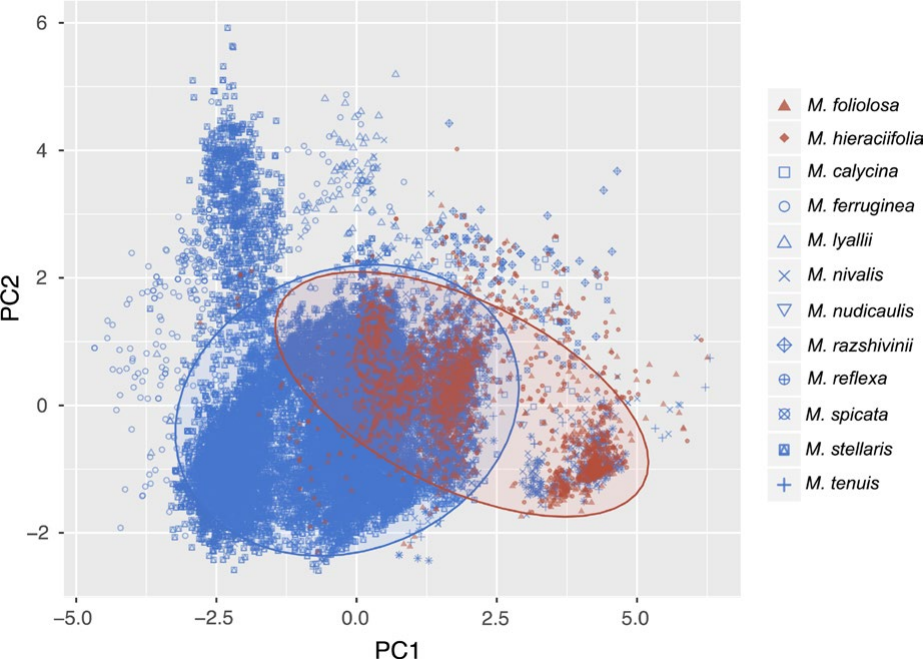
Plot of principal component 1 versus principal component 2 for the PCA performed on the 12 *Micranthes* species that occur in the Arctic. All species shown, with species that are predicted to lose habitat in blue and species that gain habitat in red. Ellipses are the 95% confidence intervals for all species that lose habitat (blue) and all species that gain habitat (red). Statistically significant separation among species that gain and lose habitat occurs along both PCA axis (overall: *F*_1,21960_ = 1598.2, *p* < 0.001; *x*‐axis: *F*_1, 21960_ = 4518.4, *p* < 0.001; *y*‐axis *F*_1, 21960_ = 49.2, *p* < 0.001). The *x*‐axis explains 44.8% of the variation and the *y*‐axis explains 21.5%

For the analysis of Arctic species (including species that span Arctic and temperate biomes), the PC1 was weighted most heavily on latitude (positive loading) and average precipitation (negative loading), while the PC2 was significantly influenced by elevation (Figure 5). For this analysis, three principal components had eigenvalues greater than 1, and the third principal component (PC3) was weighted most heavily by silt (negative loading) and soil pH (positive loading). These three components explain 83% of the variance. The MANOVA demonstrated that these three components are able to differentiate between Arctic species that gain or lose habitat (*p *<* *0.001) (Supporting Information Appendix S5).

**Figure 6:**
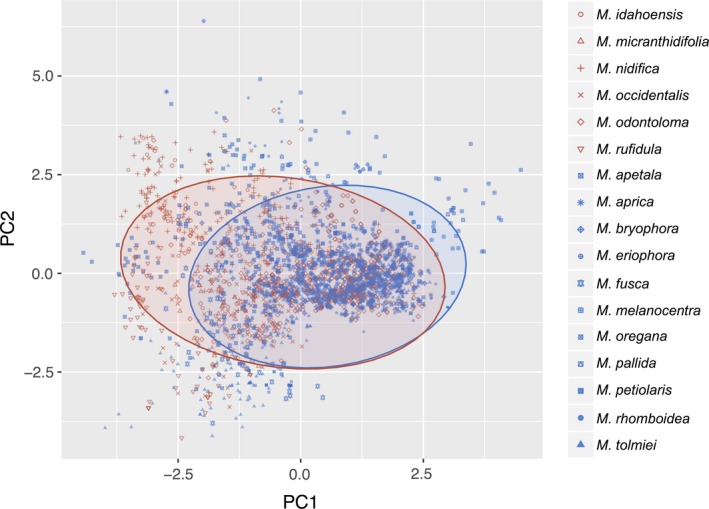
Plot of principal component 1 versus principal component 2 for the PCA performed on *Micranthes* species that occur in mountains. The montane species that are predicted to lose habitat are in blue and the species that are predicted to gain the habitat are shown in red. Ellipses show the 95% confidence intervals for all species that lose habitat (blue) and all species that gain habitat (red).Statistically significant separation among species that gain and lose habitat occurs along both PCA axis (overall: *F*_1,2761_ = 146.1, *p* < 0.001; *x*‐axis: *F*_1, 2761_ = 265.2, *p* < 0.001; *y*‐axis *F*_1, 2761_ = 7.8, *p* < 0.01). The *x*‐axis explains 37.0% of the variation and the *y*‐axis explains 26.4%

For species that only occur in mountains (does not include species that occur in both mountains and Arctic), three components had eigenvalues greater than 1, accounting for 85% of the variance (Figure 6). PC1 was dominated by elevation, while PC2 was influenced by soil pH (positive loading) and precipitation (negative loading). PC3 is driven by the remaining three variables—latitude and silt (both positive) and temperature (negative). The MANOVA demonstrated that these three components are able to differentiate between mountain species that gain or lose habitat (*p *<* *0.01) (Supporting Information Appendix S5).

**Figure 7:**
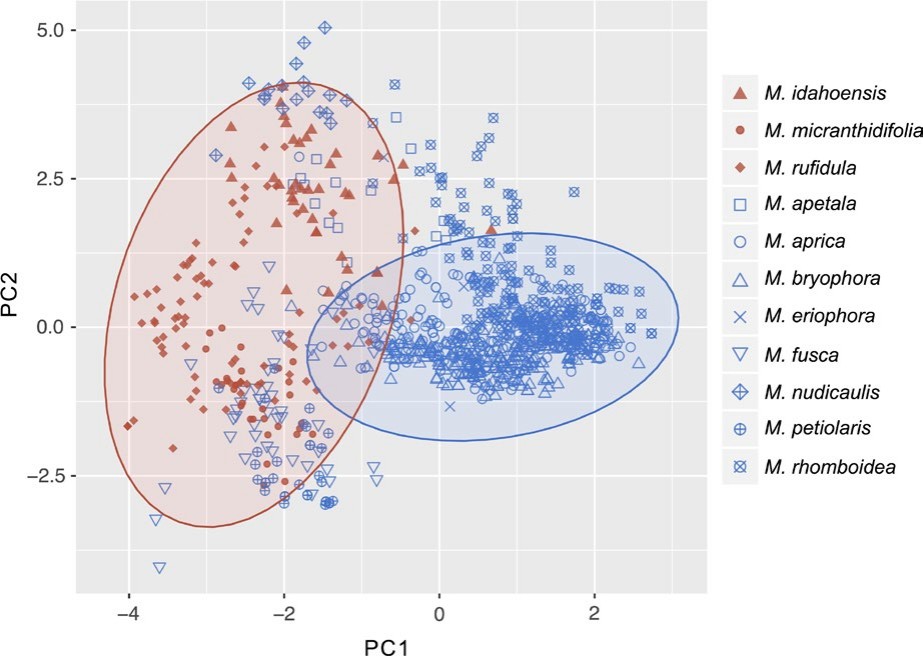
Plot of principal component 1 versus principal component 2 for the PCA performed on *Micranthes* species that are classified as narrow endemics. PCA run on all 11 species, with species that are predicted to lose habitat shown in blue and the species that are predicted to gain habitat shown in red. Statistically significant separation among species that gain and lose habitat occurs along both PCA axis (overall: *F*_1,1007_ = 257.3, *p* < 0.001; *x*‐axis: *F*_1, 1007_ = 702.1, *p* < 0.001; *y*‐axis *F*_1, 1007_ = 24.3, *p* < 0.001). The *x*‐axis explains 39.9% of the variation and the *y*‐axis explains 25.9%

Finally, for endemic species, three components had eigenvalues greater than 1 and accounted for 85% of the variability. PC1 was dominated by elevation (positive), PC2 was influenced by latitude and soil pH (both positive) and average precipitation (negative), and PC3 was most influenced by average temperature (positive). The MANOVA for this group demonstrated that the first two components are able to differentiate between endemic species that gain or lose habitat (*p *<* *0.001). The third component was not significant in this distinction (*p *>* *0.05) (Supporting Information Appendix S5). In Figure 7, which is a PCA with all 11 species classified as narrow endemics, on the *x*‐axis species occurring at higher elevations are found on the positive side of the graph, while on the *y*‐axis species found in high soil pH and low precipitation areas are found on the top of the graph.

## 4 DISCUSSION

Climate change is expected to have a negative impact on cold environments in the future, and this will put strong selection pressures on cold‐adapted plant species (Abeli et al., 2009; Gottfried et al., 2012; Stocker et al., 2013). Our results suggest, in agreement with previous work (Abeli et al., 2009; Ernakovich et al., 2014; Thuiller et al., 2005), that although Arctic and alpine ecosystems have similar climate conditions with short and cold growing seasons, climate change will lead to varied responses even among closely related species. Nine of 12 species of *Micranthes* that occur in the Arctic lose habitat (83%), while six out of 17 species that occur in mountains (65%) lose habitat as a result of predicted climate change. This supports research that has shown that Arctic sites are projected to warm more than montane sites (Ernakovich et al., 2014; Stocker et al., 2013). A more detailed study on *Micranthes* taking into account the dispersal ability, genetic diversity, and phenology of these species would help to elucidate these patterns (CaraDonna & Inouye, 2015; Cotto et al., 2017; Kearney & Porter, 2009), but our investigation into climatic and abiotic drivers of this pattern provides initial insights into this variation in response to a changing climate.

Our first objective was to test how the geographic areas of Arctic plants will change as a result of climate change. Of the seven species that exclusively occur in the Arctic, five lose habitat under future climate change models and two experience increases in total amount of habitat. The two species that are predicted to have an increase in habitat, *M. foliolosa* and *M. hieraciifolia* (Waldst. & Kit. Ex Willd.) Haw. (Figure 3g,i; Supporting Information Figure S1), are the only two species in our analyses with widespread, circumpolar, and circumboreal distributions. Notably, the original shapefiles for both species were the largest of any species used in this analysis and they considerably overlap with one another. In a meta‐analysis by Slatyer, Hirst, and Sexton (2013) a positive correlation between niche breadth and range size was supported across both taxonomic groups and spatial scales, and their results support that niche breadth can explain at least some of the variation in geographical range size among taxa. Therefore, one explanation for why these two Arctic species show an increase in suitable habitat under projected future climate change scenarios is that with their widespread distribution they have broad ecological niche tolerances. Although we do not have information on the genetic diversity of these species, the correlation between increased genetic diversity and increased range size allows us to hypothesize that these two circumpolar species may have increased genetic diversity. High levels of genetic diversity within a species have been correlated with wider ecological breadth, and consequently, these species are thought to be able to cope with a broader range of climatic changes (Theodoridis, Patsiou, Randin, & Conti, 2017). In spite of the fact that these species are predicted to maintain large geographic areas despite changing climate, it is notable that less than half of the current habitat for both species overlaps with predicted future habitat (Table 2). Therefore, for the future geographic area to be realized, both *M. foliolosa* and *M. hieraciifolia* would be required to disperse to and occupy >300,000 km^2^ and >400,000 km^2^ of novel habitat, respectively, by 2080. Consequently, without knowledge about the dispersal ability of these two species, it is not possible to predict if they will be able to migrate to new areas within the appropriate time frame (Bakkenes, Eickhout, & Alkemade, 2006).

**Table 2:** Results from ecological niche models (ENMs) for dataset corrected for sampling bias

Taxon	Schoener’s D	Current area	Future area	Overlap	Future/current
*M. apetala*	0.795	27,671	698	698	0.025
*M. aprica*	0.396	18,483	3,416	2,766	0.185
*M. bryophora*	0.823	14,114	11,908	10,627	0.844
*M. calycina*	0.330	164,649	34,613	27,574	0.210
*M. eriophora*	0.901	118,750	54,895	54,895	0.462
*M. ferruginea*	0.836	413,143	105,937	105,888	0.256
*M. foliolosa*	0.486	256,861	489,224	100,244	1.905
*M. fusca*	0.811	75,747	23,500	23,114	0.310
*M. hieraciifolia*	0.321	152,947	545,160	58,708	3.564
*M. idahoensis*	0.769	50,269	465,495	49,296	9.260
*M. lyallii*	0.626	468,111	304,502	135,051	0.650
*M. melanocentra*	0.892	794,120	499,373	455,066	0.629
*M. micranthidifolia*	0.845	46,261	72,821	38,427	1.574
*M. nidifica*	0.777	285,441	458,629	226,856	1.607
*M. nivalis*	0.778	3,349,750	2,263,874	2,227,814	0.676
*M. nudicaulis*	0.816	31,252	6,451	5,820	0.206
*M. occidentalis*	0.727	219,339	1,134,623	196,534	5.173
*M. odontoloma*	0.895	290,213	309,436	263,911	1.066
*M. oregana*	0.751	208,639	92,294	67,997	0.442
*M. pallida*	0.838	370,755	276,002	234,287	0.744
*M. petiolaris*	0.785	2,174	613	487	0.282
*M. razshivinii*	0.712	196,858	50,145	38,189	0.255
*M. reflexa*	0.775	566,316	223,471	206,545	0.395
*M. rhomboidea*	0.611	52,595	20,940	5,536	0.398
*M. rufidula*	0.754	115,881	223,091	109,287	1.925
*M. spicata*	0.747	440,622	113,734	96,267	0.258
*M. stellaris*	0.821	963,529	602,365	593,039	0.625
*M. tenuis*	0.656	2,343,503	1,214,732	1,130,145	0.518
*M. tolmiei*	0.591	540,079	146,606	142,982	0.271

*Note*

Schoener’s *D* is a statistical measures for niche overlap. Current, Future, and Overlap is the amount of land area (in square kilometers) predicted by ENMS using the TTP threshold to be suitable habitat. Future/Current is the future area divided by the current area.

Five species occur in both the Arctic and temperate biomes. All of these species are predicted to experience a decrease in their present range size as a result of projected climate change. One species, *M. ferruginea* (Graham) Brouillet & Gornall (Figure 3f; Supporting Information Figure S1), is predicted to experience a substantial decrease in available niche space (74%). Our models predict that the five species spanning from Arctic biomes to temperate mountains will lose the most southern parts of their ranges (Supporting Information Figure S1F,K,O,AA,BB). Many cold‐adapted species have been shown to reach their southernmost occurrences in temperate mountain chains, and these populations are thought to be hotspots of unique ecological conditions and/or contain unique alleles as a result of acting as refugia during glacial expansions (Abeli et al., 2009). Subsequently, further in‐depth studies on the genetic diversity and biology of these species, and those with similar distributions, are warranted. Further, these southern populations may harbor unique adaptations, phylogenetic history, and evolutionary potential, and therefore, should be given special conservation status (Abeli et al., 2009).

Additionally, our analyses with Arctic plants recovered range shifts into cooler, northern habitats, which is in support of previous research (Jump & Penuelas, 2005; Sturm et al., 2001). The results of the PCA for Arctic species suggest that species with a high component score will be those occurring in high latitudes in dry, cold climates. This, in combination with the statistically significant relationship (*p *<* *0.001) between high PC1 scores (most heavily weighted by an inverse relationship of latitude and precipitation) and loss of habitat (Figure 5), suggests that among Arctic species those at higher latitudes are more likely at risk for losing substantial habitat as a result of climate change.

Second, we tested if species of *Micranthes* occupying mountains will be impacted by climate change. The unique climatic conditions in mountains have promoted highly specialized species with strong adaptations to the limited opportunities for growth and survival. Additionally, the narrow habitat tolerances of the mountain flora, in conjunction with marginal habitats for many species, have been shown to be highly impacted by climate change (Thuiller et al., 2005). In our analyses, of the 17 montane species, 11 lose habitat and six gain habitat. In Figure 6, the montane species that are predicted to increase in geographical area under future climate models are found at lower elevations (left side of *y*‐axis). In contrast, the species that are predicted to experience a decrease in suitable habitat are clustered toward higher elevations (right side of *x*‐axis). Therefore, it can be inferred that species of *Micranthes* restricted to higher elevations may be more threatened by climate change than mountain species of the same genus that occupy lower elevations. This supports the theory that species at upper elevational limits are at a disadvantage in terms of range expansion (Chapin & Körner, 1994; Elsen & Tingley, 2015; Jump & Penuelas, 2005).

The difference in response between Arctic and alpine plants can likely be explained, in part, by latitude. Latitude determines many ecosystem processes across biomes by affecting day length; in Arctic and alpine habitats this has a notable impact on the timing of snowmelt, cues for plant phenology, and winter temperatures (Ernakovich et al., 2014). Additionally, the effects of latitude are more pronounced toward the poles, and in two of our analyses, i.e., all cold‐adapted *Micranthes* (Figure 4) and *Micranthes* occurring in the Arctic (Figure 5), latitude was a significant component (Supporting Information Appendix S5). Therefore, as warming climates differentially change abiotic factors that are fundamental to plant growth in these biomes, it follows that the flora of these regions would have divergent responses.

Third, we tested which variables affect plants that are narrowly endemic in terms of their response to climate change. Our results suggest that, under future predicted climate change, species with small fundamental niches are susceptible to habitat loss if they are restricted to higher elevations (Figure 7). For this analysis, PC1 is dominated by elevation, suggesting that this variable is critical in the ability of a species to expand its range. The second component is heavily weighted by latitude and soil pH with a negative correlation to average precipitation. The inverse correlation of soil pH and precipitation is supported in the literature, as more acidic, or lower pH soils, are found in wetter areas (Slessarev et al., 2016). These results support the hypothesis of mountains as islands, and that these high‐elevation islands are spatial and temporally isolated (Billings, 1974; Hadley, 1987).

## 5 CONCLUSION

The goals of this study were to predict how plants found in cold habitats respond to climate change, whether there is any variation in their response, and which variables significantly affect it. First, we found that most Arctic species will be negatively affected by climate change, while the response by mountain species is not as uniform. This result aligns with the notion that climate change is altering abiotic factors such as temperature and timing of snowmelt, and these factors are more severely impacted in Arctic than alpine habitats due to high latitudes (Ernakovich et al., 2014). However, within montane species, the higher elevation species are more likely to be negatively impacted by climate change. Overall, this research demonstrates that there is a variable response to predicted warming climates in regard to habitat expansion or contraction, and our results suggest several general trends for plants occupying these habitats. Specifically, our study supports the hypotheses that species at upper elevational and latitudinal limits are more susceptible to the negative impacts of climate change, and these results can be used to generate new hypotheses to be tested through subsequent fieldwork and ecological and population genetic analyses (Graham, Ron, Santos, Schneider, & Moritz, 2004).

## AUTHORS CONTRIBUTION

R.L.S, D.E.S, and N.C. designed the research. R.L.S. ran the analyses and analyzed the results. R.L.S, D.E.S, and N.C. wrote the manuscript.

## DATA ACCESSIBILITY

All occurrence data, shapefiles, and the ecological niche model results generated from this study are available as raster grids from the Dryad repository (https://doi.org/10.5061/dryad.7294nh2).
