# Supplementary material for: The future of cold‐adapted plants in changing climates: Micranthes (Saxifragaceae) as a case study
Source: Ecol Evol. 2018 Jun 25;8(14):7164–77. doi: 10.1002/ece3.4242 (PMC6065370; doi:10.1002/ece3.4242)
Supplement: Supplementary file 1 [file ECE3-8-7164-s001.pdf]

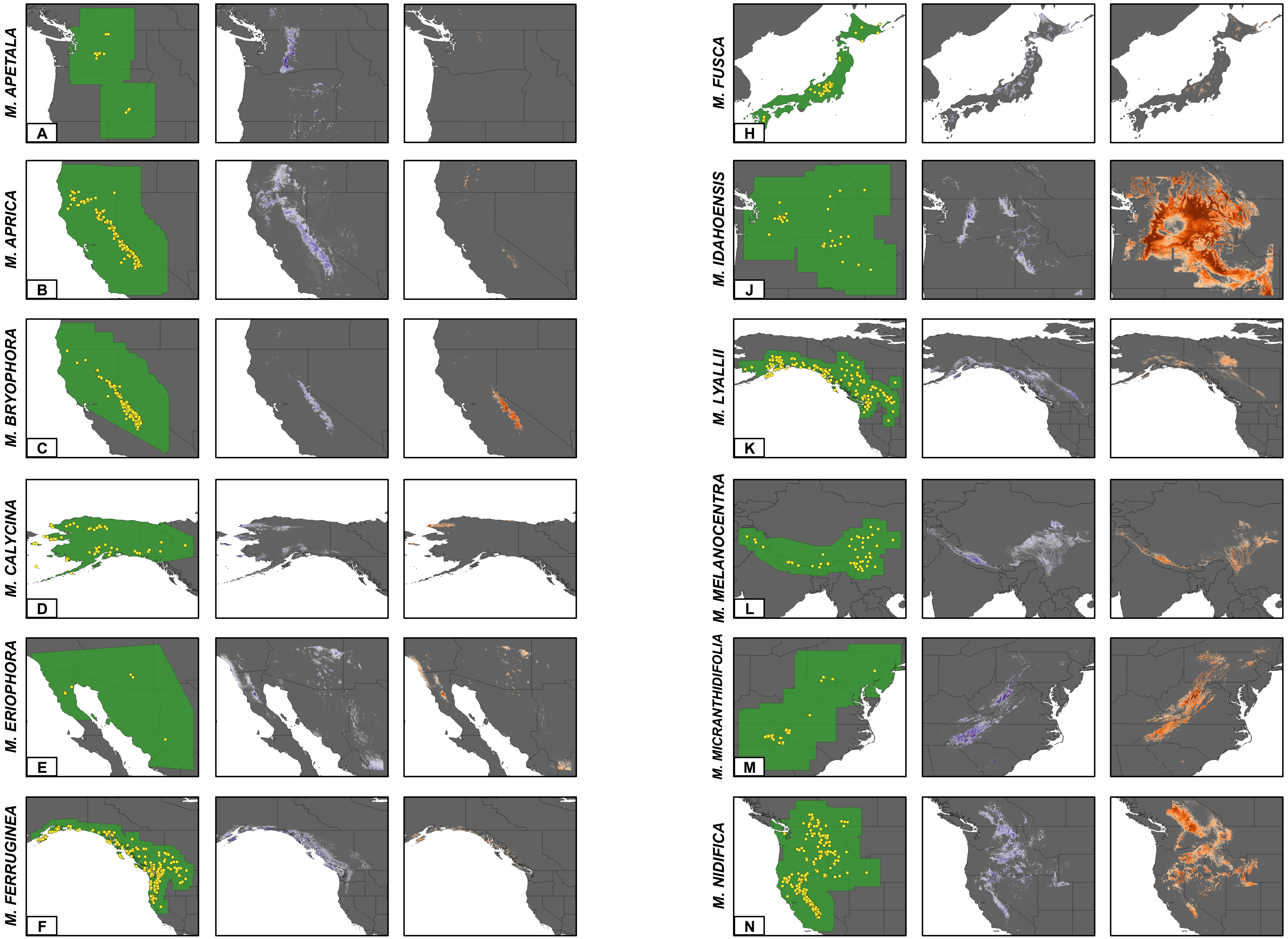
M. APETALA
A
M. APRICA
B
M. BRYOPHORA
C
M. CALYCINA
D
M. ERIOPHORA
E
M. FERRUGINEA
F
M. FUSCA
H
M. IDAHOENSIS
J
M. LYALLII
K
M. MELANOCENTRA
L
M. MICRANTHIDIFOLIA
M
M. NIDIFICA
N

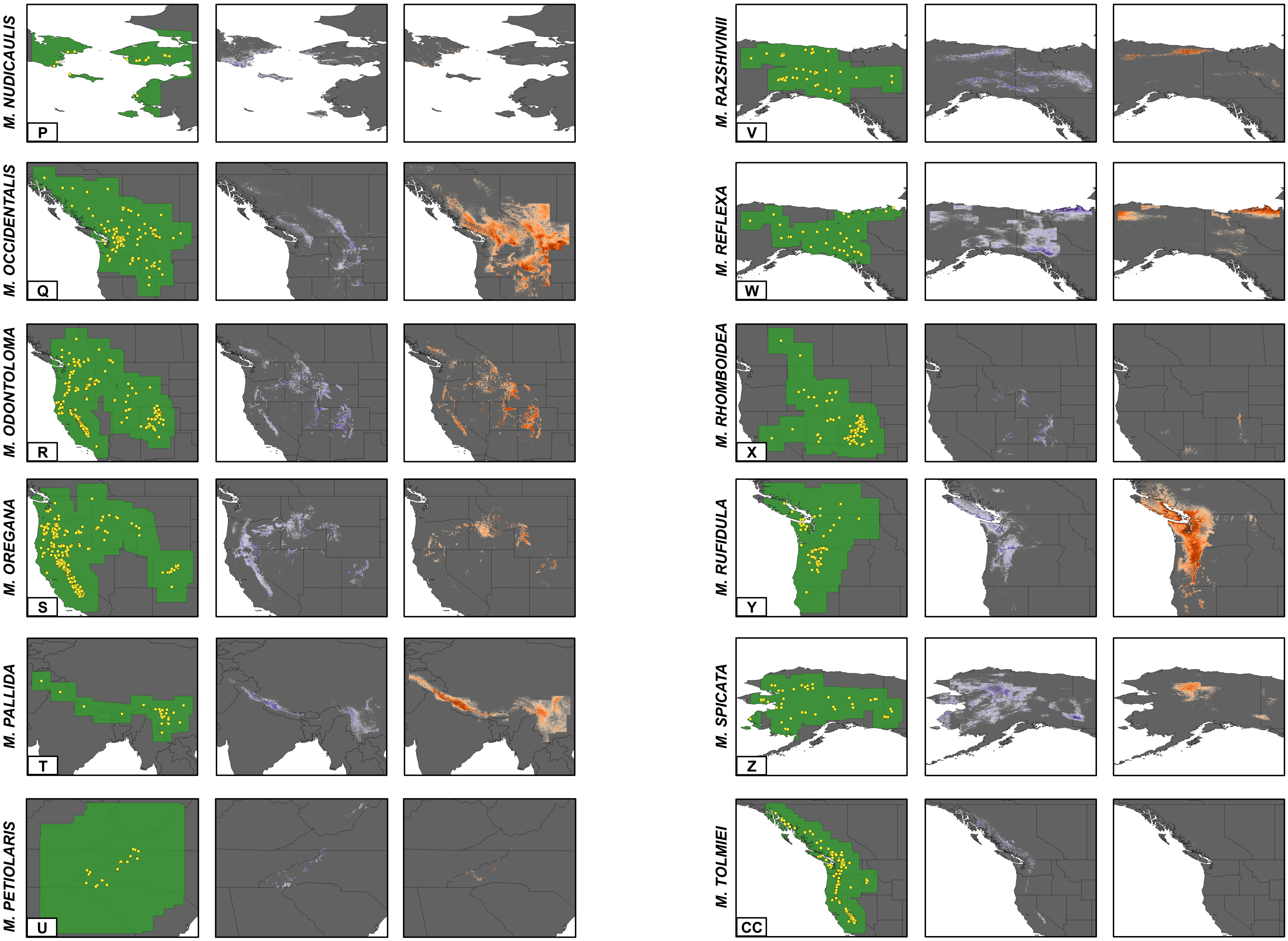
M. NUDICAULIS
P
M. OCCIDENTALIS
Q
M. ODONTOLOMA
R
M. OREGANA
S
M. PALLIDA
T
M. PETIOLARIS
U
M. RAZSHIVINII
V
M. REFLEXA
W
M. RHOMBOIDEA
X
M. RUFIDULA
Y
M. SPICATA
Z
M. TOLMIEI
CC

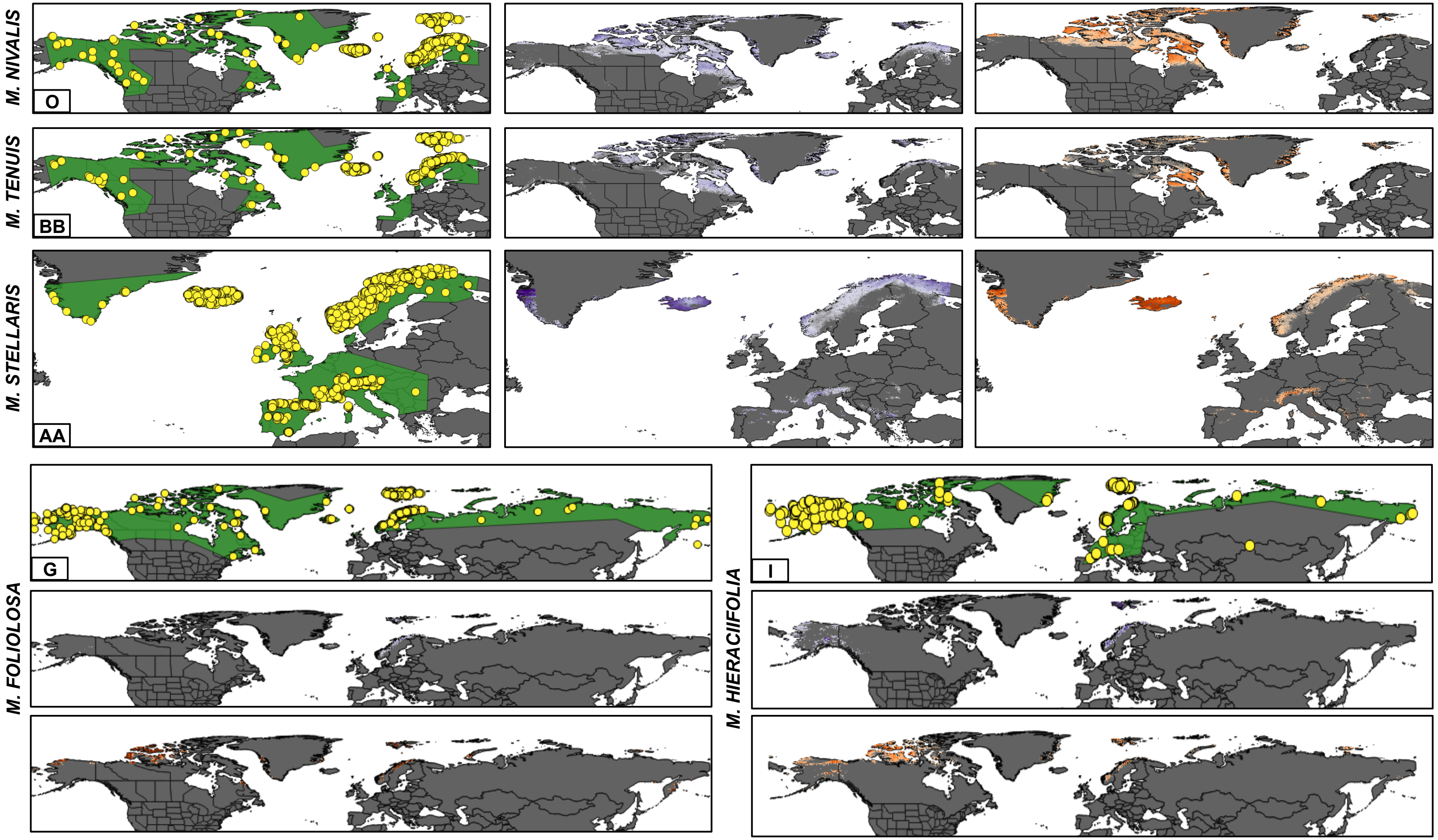

Fig. S1. a–cc. Ecological niche models for all species. Green areas represent the original shapefiles that were used to trim layers. They represent all current and potential habitat. Yellow dots are accessions used to train and test the models. Purple areas are designating current fundamental niche space. Orange areas are designating future predicted fundamental niche space. Darker shades represent more suitable areas. Letters correspond to species listed in Table 1 and images are alphabetical, except for species with larger distributions: g) *M. foliolosa*, i) *M. hieraciifolia*, o) *M. nivalis*, aa*) M. stellaris*, bb) *M. tenuis*.
