# Supplementary figures and images for: The future of cold‐adapted plants in changing climates: Micranthes (Saxifragaceae) as a case study
Source: Ecol Evol. 2018 Jun 25;8(14):7164–77. doi: 10.1002/ece3.4242 (PMC6065370; doi:10.1002/ece3.4242)

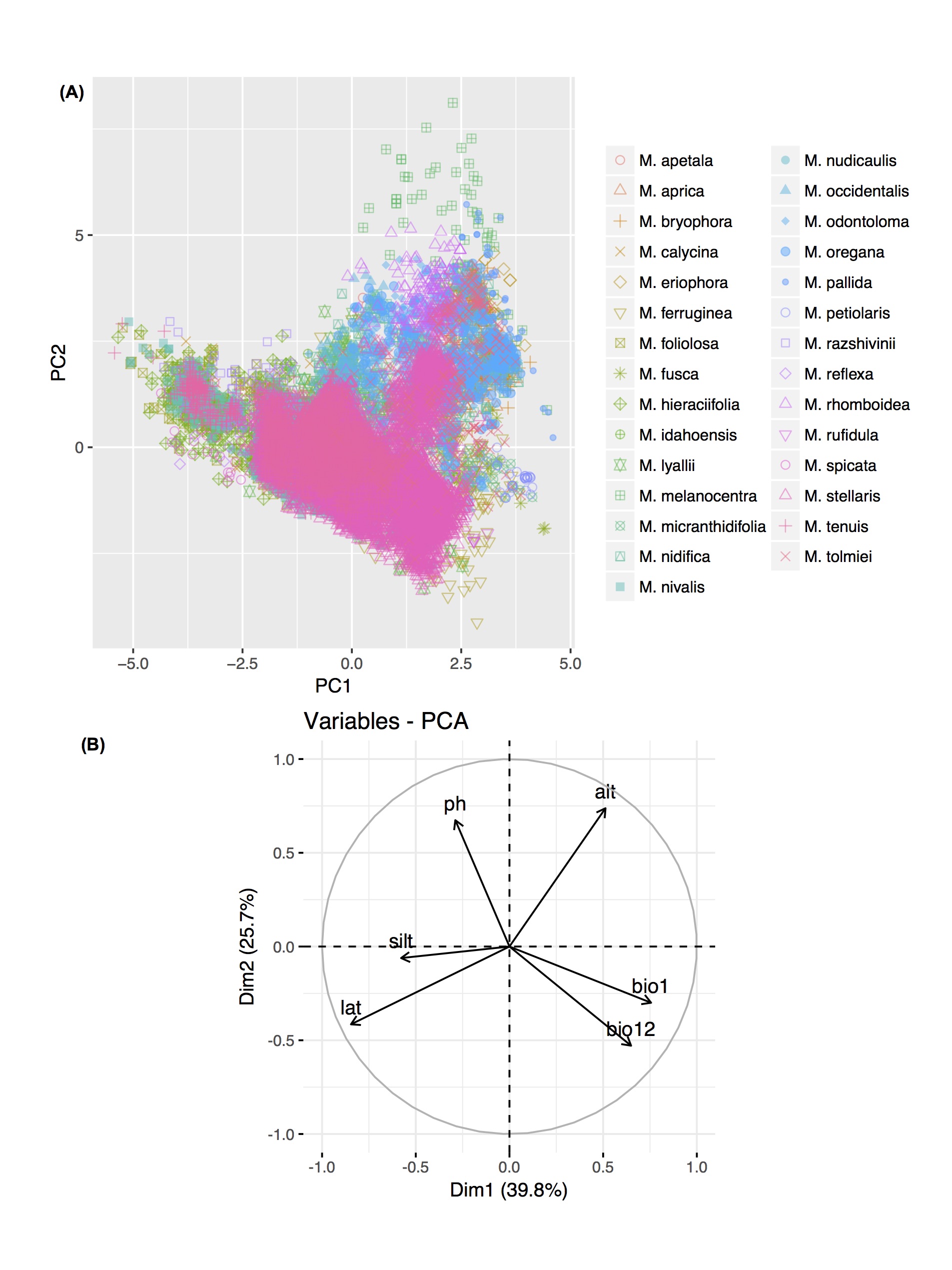

Supplement: Supplementary file 2 [file ECE3-8-7164-s002.jpg]
