# Supplementary material for: The future of cold‐adapted plants in changing climates: Micranthes (Saxifragaceae) as a case study
Source: Ecol Evol. 2018 Jun 25;8(14):7164–77. doi: 10.1002/ece3.4242 (PMC6065370; doi:10.1002/ece3.4242)
Supplement: Supplementary file 3 [file ECE3-8-7164-s003.pdf]

Correlation table for climate variables for M. apetala

	bio1	bio2	bio3	bio4	bio5	bio6	bio7	bio8	bio9	bio10	bio11	bio12	bio13	bio14	bio15	bio16	bio17	bio18	bio19
bio1	1.000	0.305	0.441	0.001	0.766	0.762	0.137	0.481	0.763	0.915	0.889	-0.166	-0.138	-0.516	0.201	-0.130	-0.409	-0.492	-0.132
bio2	0.305	1.000	0.727	0.451	0.789	-0.254	0.858	0.087	0.478	0.495	0.097	-0.671	-0.660	-0.766	-0.453	-0.654	-0.767	-0.763	-0.652
bio3	0.441	0.727	1.000	-0.262	0.533	0.265	0.278	0.258	0.557	0.332	0.538	-0.167	-0.162	-0.488	-0.041	-0.151	-0.367	-0.433	-0.153
bio4	0.001	0.451	-0.262	1.000	0.507	-0.582	0.841	-0.149	0.033	0.400	-0.451	-0.756	-0.739	-0.524	-0.529	-0.745	-0.666	-0.584	-0.739
bio5	0.766	0.789	0.533	0.507	1.000	0.189	0.735	0.291	0.712	0.921	0.468	-0.632	-0.605	-0.834	-0.238	-0.599	-0.806	-0.831	-0.597
bio6	0.762	-0.254	0.265	-0.582	0.189	1.000	-0.527	0.443	0.502	0.454	0.934	0.414	0.430	0.020	0.571	0.437	0.182	0.072	0.433
bio7	0.137	0.858	0.278	0.841	0.735	-0.527	1.000	-0.054	0.269	0.483	-0.240	-0.833	-0.821	-0.735	-0.600	-0.820	-0.823	-0.768	-0.816
bio8	0.481	0.087	0.258	-0.149	0.291	0.443	-0.054	1.000	0.128	0.383	0.496	-0.023	-0.025	-0.228	-0.013	-0.018	-0.153	-0.129	-0.023
bio9	0.763	0.478	0.557	0.033	0.712	0.502	0.269	0.128	1.000	0.724	0.679	-0.144	-0.102	-0.515	0.303	-0.094	-0.404	-0.547	-0.095
bio10	0.915	0.495	0.332	0.400	0.921	0.454	0.483	0.383	0.724	1.000	0.636	-0.462	-0.430	-0.699	-0.043	-0.425	-0.654	-0.697	-0.424
bio11	0.889	0.097	0.538	-0.451	0.468	0.934	-0.240	0.496	0.679	0.636	1.000	0.187	0.204	-0.240	0.407	0.214	-0.076	-0.188	0.210
bio12	-0.166	-0.671	-0.167	-0.756	-0.632	0.414	-0.833	-0.023	-0.144	-0.462	0.187	1.000	0.995	0.730	0.671	0.995	0.890	0.833	0.995
bio13	-0.138	-0.660	-0.162	-0.739	-0.605	0.430	-0.821	-0.025	-0.102	-0.430	0.204	0.995	1.000	0.680	0.729	1.000	0.851	0.786	1.000
bio14	-0.516	-0.766	-0.488	-0.524	-0.834	0.020	-0.735	-0.228	-0.515	-0.699	-0.240	0.730	0.680	1.000	0.204	0.676	0.955	0.956	0.673
bio15	0.201	-0.453	-0.041	-0.529	-0.238	0.571	-0.600	-0.013	0.303	-0.043	0.407	0.671	0.729	0.204	1.000	0.728	0.391	0.259	0.729
bio16	-0.130	-0.654	-0.151	-0.745	-0.599	0.437	-0.820	-0.018	-0.094	-0.425	0.214	0.995	1.000	0.676	0.728	1.000	0.850	0.784	1.000
bio17	-0.409	-0.767	-0.367	-0.666	-0.806	0.182	-0.823	-0.153	-0.404	-0.654	-0.076	0.890	0.851	0.955	0.391	0.850	1.000	0.978	0.847
bio18	-0.492	-0.763	-0.433	-0.584	-0.831	0.072	-0.768	-0.129	-0.547	-0.697	-0.188	0.833	0.786	0.956	0.259	0.784	0.978	1.000	0.782
bio19	-0.132	-0.652	-0.153	-0.739	-0.597	0.433	-0.816	-0.023	-0.095	-0.424	0.210	0.995	1.000	0.673	0.729	1.000	0.847	0.782	1.000

Correlation table for climate variables for M. aprica

	bio1	bio2	bio3	bio4	bio5	bio6	bio7	bio8	bio9	bio10	bio11	bio12	bio13	bio14	bio15	bio16	bio17	bio18	bio19
bio1	1.000	0.070	0.144	0.032	0.808	0.836	-0.001	0.714	0.749	0.929	0.926	-0.188	-0.088	-0.856	0.453	-0.104	-0.788	-0.798	-0.085
bio2	0.070	1.000	-0.284	0.717	0.574	-0.395	0.875	0.114	0.089	0.326	-0.212	-0.644	-0.652	-0.065	-0.509	-0.649	-0.250	-0.268	-0.646
bio3	0.144	-0.284	1.000	-0.847	-0.285	0.495	-0.699	0.175	0.073	-0.181	0.443	0.430	0.484	-0.311	0.588	0.479	-0.179	-0.159	0.487
bio4	0.032	0.717	-0.847	1.000	0.575	-0.492	0.961	0.028	0.033	0.398	-0.342	-0.687	-0.722	0.111	-0.692	-0.717	-0.082	-0.098	-0.720
bio5	0.808	0.574	-0.285	0.575	1.000	0.383	0.576	0.574	0.655	0.952	0.542	-0.526	-0.465	-0.640	-0.007	-0.474	-0.689	-0.712	-0.461
bio6	0.836	-0.395	0.495	-0.492	0.383	1.000	-0.534	0.561	0.656	0.586	0.972	0.269	0.370	-0.775	0.790	0.355	-0.588	-0.593	0.370
bio7	-0.001	0.875	-0.699	0.961	0.576	-0.534	1.000	0.029	0.019	0.352	-0.364	-0.719	-0.753	0.099	-0.706	-0.748	-0.110	-0.127	-0.750
bio8	0.714	0.114	0.175	0.028	0.574	0.561	0.029	1.000	0.285	0.669	0.658	-0.174	-0.105	-0.604	0.173	-0.114	-0.582	-0.547	-0.108
bio9	0.749	0.089	0.073	0.033	0.655	0.656	0.019	0.285	1.000	0.697	0.693	-0.105	-0.030	-0.692	0.490	-0.042	-0.609	-0.666	-0.024
bio10	0.929	0.326	-0.181	0.398	0.952	0.586	0.352	0.669	0.697	1.000	0.725	-0.422	-0.344	-0.745	0.159	-0.356	-0.754	-0.766	-0.340
bio11	0.926	-0.212	0.443	-0.342	0.542	0.972	-0.364	0.658	0.693	0.725	1.000	0.093	0.199	-0.850	0.695	0.184	-0.711	-0.713	0.202
bio12	-0.188	-0.644	0.430	-0.687	-0.526	0.269	-0.719	-0.174	-0.105	-0.422	0.093	1.000	0.989	0.151	0.522	0.994	0.457	0.455	0.989
bio13	-0.088	-0.652	0.484	-0.722	-0.465	0.370	-0.753	-0.105	-0.030	-0.344	0.199	0.989	1.000	0.039	0.614	0.998	0.342	0.340	0.999
bio14	-0.856	-0.065	-0.311	0.111	-0.640	-0.775	0.099	-0.604	-0.692	-0.745	-0.850	0.151	0.039	1.000	-0.579	0.058	0.917	0.901	0.026
bio15	0.453	-0.509	0.588	-0.692	-0.007	0.790	-0.706	0.173	0.490	0.159	0.695	0.522	0.614	-0.579	1.000	0.598	-0.369	-0.408	0.619
bio16	-0.104	-0.649	0.479	-0.717	-0.474	0.355	-0.748	-0.114	-0.042	-0.356	0.184	0.994	0.998	0.058	0.598	1.000	0.366	0.365	0.998
bio17	-0.788	-0.250	-0.179	-0.082	-0.689	-0.588	-0.110	-0.582	-0.609	-0.754	-0.711	0.457	0.342	0.917	-0.369	0.366	1.000	0.976	0.331
bio18	-0.798	-0.268	-0.159	-0.098	-0.712	-0.593	-0.127	-0.547	-0.666	-0.766	-0.713	0.455	0.340	0.901	-0.408	0.365	0.976	1.000	0.330
bio19	-0.085	-0.646	0.487	-0.720	-0.461	0.370	-0.750	-0.108	-0.024	-0.340	0.202	0.989	0.999	0.026	0.619	0.998	0.331	0.330	1.000

Correlation table for climate variables for M. bryophora

	bio1	bio2	bio3	bio4	bio5	bio6	bio7	bio8	bio9	bio10	bio11	bio12	bio13	bio14	bio15	bio16	bio17	bio18	bio19
bio1	1.000	0.108	0.017	0.159	0.840	0.812	0.100	0.668	0.724	0.938	0.925	-0.167	-0.078	-0.844	0.341	-0.097	-0.774	-0.774	-0.079
bio2	0.108	1.000	-0.375	0.793	0.592	-0.408	0.914	0.183	0.077	0.368	-0.208	-0.686	-0.687	-0.110	-0.573	-0.684	-0.332	-0.357	-0.680
bio3	0.017	-0.375	1.000	-0.835	-0.343	0.440	-0.706	0.017	0.056	-0.278	0.326	0.653	0.684	-0.183	0.582	0.679	0.057	0.077	0.683
bio4	0.159	0.793	-0.835	1.000	0.630	-0.428	0.968	0.194	0.054	0.490	-0.224	-0.822	-0.835	-0.030	-0.694	-0.831	-0.302	-0.318	-0.830
bio5	0.840	0.592	-0.343	0.630	1.000	0.395	0.611	0.597	0.623	0.960	0.586	-0.535	-0.471	-0.685	-0.075	-0.484	-0.757	-0.775	-0.470
bio6	0.812	-0.408	0.440	-0.428	0.395	1.000	-0.485	0.462	0.653	0.567	0.967	0.354	0.440	-0.738	0.746	0.421	-0.501	-0.499	0.435
bio7	0.100	0.914	-0.706	0.968	0.611	-0.485	1.000	0.169	0.031	0.425	-0.276	-0.813	-0.828	-0.017	-0.715	-0.823	-0.289	-0.308	-0.822
bio8	0.668	0.183	0.017	0.194	0.597	0.462	0.169	1.000	0.189	0.660	0.581	-0.179	-0.123	-0.533	-0.008	-0.132	-0.533	-0.477	-0.130
bio9	0.724	0.077	0.056	0.054	0.623	0.653	0.031	0.189	1.000	0.655	0.697	-0.046	0.023	-0.685	0.469	0.007	-0.581	-0.645	0.027
bio10	0.938	0.368	-0.278	0.490	0.960	0.567	0.425	0.660	0.655	1.000	0.739	-0.428	-0.355	-0.754	0.055	-0.370	-0.786	-0.789	-0.354
bio11	0.925	-0.208	0.326	-0.224	0.586	0.967	-0.276	0.581	0.697	0.739	1.000	0.163	0.256	-0.824	0.612	0.235	-0.647	-0.640	0.253
bio12	-0.167	-0.686	0.653	-0.822	-0.535	0.354	-0.813	-0.179	-0.046	-0.428	0.163	1.000	0.990	0.105	0.636	0.995	0.460	0.469	0.991
bio13	-0.078	-0.687	0.684	-0.835	-0.471	0.440	-0.828	-0.123	0.023	-0.355	0.256	0.990	1.000	0.005	0.709	0.998	0.356	0.364	0.999
bio14	-0.844	-0.110	-0.183	-0.030	-0.685	-0.738	-0.017	-0.533	-0.685	-0.754	-0.824	0.105	0.005	1.000	-0.480	0.028	0.892	0.869	-0.004
bio15	0.341	-0.573	0.582	-0.694	-0.075	0.746	-0.715	-0.008	0.469	0.055	0.612	0.636	0.709	-0.480	1.000	0.688	-0.208	-0.251	0.709
bio16	-0.097	-0.684	0.679	-0.831	-0.484	0.421	-0.823	-0.132	0.007	-0.370	0.235	0.995	0.998	0.028	0.688	1.000	0.385	0.395	0.998
bio17	-0.774	-0.332	0.057	-0.302	-0.757	-0.501	-0.289	-0.533	-0.581	-0.786	-0.647	0.460	0.356	0.892	-0.208	0.385	1.000	0.971	0.348
bio18	-0.774	-0.357	0.077	-0.318	-0.775	-0.499	-0.308	-0.477	-0.645	-0.789	-0.640	0.469	0.364	0.869	-0.251	0.395	0.971	1.000	0.359
bio19	-0.079	-0.680	0.683	-0.830	-0.470	0.435	-0.822	-0.130	0.027	-0.354	0.253	0.991	0.999	-0.004	0.709	0.998	0.348	0.359	1.000

Correlation table for climate variables for M. calycina

	bio1	bio2	bio3	bio4	bio5	bio6	bio7	bio8	bio9	bio10	bio11	bio12	bio13	bio14	bio15	bio16	bio17	bio18	bio19
bio1	1.000	-0.138	0.680	-0.691	0.308	0.867	-0.623	0.180	0.806	0.555	0.907	0.649	0.654	0.581	-0.434	0.671	0.586	0.650	0.588
bio2	-0.138	1.000	0.282	0.523	0.688	-0.523	0.716	0.457	-0.152	0.354	-0.410	-0.221	-0.261	-0.246	-0.070	-0.245	-0.232	-0.102	-0.195
bio3	0.680	0.282	1.000	-0.648	0.164	0.604	-0.454	-0.071	0.618	0.144	0.691	0.678	0.590	0.635	-0.558	0.645	0.652	0.701	0.653
bio4	-0.691	0.523	-0.648	1.000	0.438	-0.926	0.964	0.429	-0.657	0.210	-0.925	-0.721	-0.685	-0.698	0.416	-0.716	-0.704	-0.668	-0.677
bio5	0.308	0.688	0.164	0.438	1.000	-0.172	0.536	0.746	0.133	0.909	-0.098	-0.089	-0.079	-0.140	-0.108	-0.078	-0.140	-0.015	-0.094
bio6	0.867	-0.523	0.604	-0.926	-0.172	1.000	-0.924	-0.191	0.748	0.132	0.988	0.723	0.723	0.681	-0.390	0.745	0.682	0.687	0.660
bio7	-0.623	0.716	-0.454	0.964	0.536	-0.924	1.000	0.454	-0.589	0.240	-0.885	-0.654	-0.650	-0.638	0.292	-0.668	-0.639	-0.595	-0.602
bio8	0.180	0.457	-0.071	0.429	0.746	-0.191	0.454	1.000	-0.056	0.724	-0.153	-0.314	-0.216	-0.355	0.184	-0.241	-0.360	-0.089	-0.389
bio9	0.806	-0.152	0.618	-0.657	0.133	0.748	-0.589	-0.056	1.000	0.329	0.781	0.633	0.612	0.568	-0.438	0.630	0.590	0.561	0.628
bio10	0.555	0.354	0.144	0.210	0.909	0.132	0.240	0.724	0.329	1.000	0.174	0.059	0.110	-0.002	-0.105	0.098	-0.006	0.115	0.025
bio11	0.907	-0.410	0.691	-0.925	-0.098	0.988	-0.885	-0.153	0.781	0.174	1.000	0.745	0.730	0.698	-0.449	0.757	0.702	0.712	0.686
bio12	0.649	-0.221	0.678	-0.721	-0.089	0.723	-0.654	-0.314	0.633	0.059	0.745	1.000	0.932	0.975	-0.567	0.970	0.983	0.872	0.975
bio13	0.654	-0.261	0.590	-0.685	-0.079	0.723	-0.650	-0.216	0.612	0.110	0.730	0.932	1.000	0.868	-0.316	0.987	0.875	0.894	0.863
bio14	0.581	-0.246	0.635	-0.698	-0.140	0.681	-0.638	-0.355	0.568	-0.002	0.698	0.975	0.868	1.000	-0.622	0.917	0.994	0.845	0.952
bio15	-0.434	-0.070	-0.558	0.416	-0.108	-0.390	0.292	0.184	-0.438	-0.105	-0.449	-0.567	-0.316	-0.622	1.000	-0.399	-0.629	-0.414	-0.608
bio16	0.671	-0.245	0.645	-0.716	-0.078	0.745	-0.668	-0.241	0.630	0.098	0.757	0.970	0.987	0.917	-0.399	1.000	0.922	0.910	0.911
bio17	0.586	-0.232	0.652	-0.704	-0.140	0.682	-0.639	-0.360	0.590	-0.006	0.702	0.983	0.875	0.994	-0.629	0.922	1.000	0.843	0.967
bio18	0.650	-0.102	0.701	-0.668	-0.015	0.687	-0.595	-0.089	0.561	0.115	0.712	0.872	0.894	0.845	-0.414	0.910	0.843	1.000	0.756
bio19	0.588	-0.195	0.653	-0.677	-0.094	0.660	-0.602	-0.389	0.628	0.025	0.686	0.975	0.863	0.952	-0.608	0.911	0.967	0.756	1.000

Correlation table for climate variables for M. eriophora

	bio1	bio2	bio3	bio4	bio5	bio6	bio7	bio8	bio9	bio10	bio11	bio12	bio13	bio14	bio15	bio16	bio17	bio18	bio19
bio1	1.000	-0.328	-0.080	0.003	0.867	0.929	-0.187	0.654	0.883	0.933	0.947	-0.351	-0.186	-0.688	0.158	-0.225	-0.660	-0.169	-0.383
bio2	-0.328	1.000	0.221	0.205	-0.084	-0.549	0.602	-0.014	-0.389	-0.258	-0.402	0.153	0.175	0.365	0.121	0.178	0.291	0.244	-0.240
bio3	-0.080	0.221	1.000	-0.888	-0.443	0.103	-0.640	-0.262	-0.295	-0.394	0.197	0.511	0.595	0.195	0.641	0.620	0.176	0.579	0.134
bio4	0.003	0.205	-0.888	1.000	0.463	-0.282	0.891	0.317	0.208	0.356	-0.313	-0.516	-0.588	-0.090	-0.616	-0.613	-0.120	-0.532	-0.279
bio5	0.867	-0.084	-0.443	0.463	1.000	0.663	0.305	0.670	0.860	0.973	0.676	-0.539	-0.425	-0.647	-0.160	-0.471	-0.626	-0.393	-0.460
bio6	0.929	-0.549	0.103	-0.282	0.663	1.000	-0.511	0.446	0.818	0.778	0.981	-0.252	-0.105	-0.686	0.213	-0.133	-0.632	-0.128	-0.184
bio7	-0.187	0.602	-0.640	0.891	0.305	-0.511	1.000	0.203	-0.053	0.128	-0.472	-0.298	-0.355	0.130	-0.456	-0.371	0.085	-0.288	-0.295
bio8	0.654	-0.014	-0.262	0.317	0.670	0.446	0.203	1.000	0.536	0.697	0.498	-0.197	-0.064	-0.254	0.130	-0.118	-0.295	0.024	-0.485
bio9	0.883	-0.389	-0.295	0.208	0.860	0.818	-0.053	0.536	1.000	0.913	0.787	-0.485	-0.401	-0.672	-0.169	-0.437	-0.645	-0.417	-0.214
bio10	0.933	-0.258	-0.394	0.356	0.973	0.778	0.128	0.697	0.913	1.000	0.775	-0.544	-0.421	-0.701	-0.105	-0.465	-0.684	-0.390	-0.443
bio11	0.947	-0.402	0.197	-0.313	0.676	0.981	-0.472	0.498	0.787	0.775	1.000	-0.191	-0.017	-0.644	0.315	-0.046	-0.604	-0.025	-0.253
bio12	-0.351	0.153	0.511	-0.516	-0.539	-0.252	-0.298	-0.197	-0.485	-0.544	-0.191	1.000	0.954	0.744	0.423	0.962	0.814	0.917	0.591
bio13	-0.186	0.175	0.595	-0.588	-0.425	-0.105	-0.355	-0.064	-0.401	-0.421	-0.017	0.954	1.000	0.597	0.632	0.991	0.668	0.971	0.404
bio14	-0.688	0.365	0.195	-0.090	-0.647	-0.686	0.130	-0.254	-0.672	-0.701	-0.644	0.744	0.597	1.000	-0.003	0.611	0.943	0.615	0.452
bio15	0.158	0.121	0.641	-0.616	-0.160	0.213	-0.456	0.130	-0.169	-0.105	0.315	0.423	0.632	-0.003	1.000	0.626	0.011	0.622	-0.130
bio16	-0.225	0.178	0.620	-0.613	-0.471	-0.133	-0.371	-0.118	-0.437	-0.465	-0.046	0.962	0.991	0.611	0.626	1.000	0.668	0.964	0.418
bio17	-0.660	0.291	0.176	-0.120	-0.626	-0.632	0.085	-0.295	-0.645	-0.684	-0.604	0.814	0.668	0.943	0.011	0.668	1.000	0.657	0.586
bio18	-0.169	0.244	0.579	-0.532	-0.393	-0.128	-0.288	0.024	-0.417	-0.390	-0.025	0.917	0.971	0.615	0.622	0.964	0.657	1.000	0.258
bio19	-0.383	-0.240	0.134	-0.279	-0.460	-0.184	-0.295	-0.485	-0.214	-0.443	-0.253	0.591	0.404	0.452	-0.130	0.418	0.586	0.258	1.000

Correlation table for climate variables for M. ferruginea

	bio1	bio2	bio3	bio4	bio5	bio6	bio7	bio8	bio9	bio10	bio11	bio12	bio13	bio14	bio15	bio16	bio17	bio18	bio19
bio1	1.000	0.259	0.750	-0.637	0.807	0.904	-0.412	-0.090	0.881	0.908	0.949	0.271	0.338	-0.074	0.434	0.351	-0.050	-0.390	0.444
bio2	0.259	1.000	0.558	0.262	0.720	-0.059	0.629	0.068	0.205	0.477	0.080	-0.594	-0.507	-0.715	0.222	-0.498	-0.718	-0.693	-0.416
bio3	0.750	0.558	1.000	-0.612	0.689	0.701	-0.271	-0.174	0.727	0.618	0.773	0.135	0.222	-0.270	0.467	0.246	-0.236	-0.532	0.364
bio4	-0.637	0.262	-0.612	1.000	-0.142	-0.884	0.908	0.370	-0.681	-0.260	-0.845	-0.686	-0.672	-0.441	-0.165	-0.687	-0.473	-0.066	-0.753
bio5	0.807	0.720	0.689	-0.142	1.000	0.521	0.180	0.010	0.691	0.944	0.621	-0.236	-0.139	-0.505	0.401	-0.128	-0.495	-0.687	-0.021
bio6	0.904	-0.059	0.701	-0.884	0.521	1.000	-0.746	-0.252	0.875	0.660	0.986	0.540	0.571	0.214	0.350	0.587	0.244	-0.171	0.670
bio7	-0.412	0.629	-0.271	0.908	0.180	-0.746	1.000	0.298	-0.469	-0.025	-0.652	-0.806	-0.767	-0.641	-0.090	-0.776	-0.667	-0.339	-0.789
bio8	-0.090	0.068	-0.174	0.370	0.010	-0.252	0.298	1.000	-0.373	0.074	-0.216	-0.170	-0.100	-0.244	0.265	-0.115	-0.245	0.101	-0.226
bio9	0.881	0.205	0.727	-0.681	0.691	0.875	-0.469	-0.373	1.000	0.746	0.891	0.339	0.381	0.028	0.343	0.398	0.054	-0.384	0.514
bio10	0.908	0.477	0.618	-0.260	0.944	0.660	-0.025	0.074	0.746	1.000	0.735	-0.041	0.052	-0.341	0.459	0.060	-0.328	-0.539	0.142
bio11	0.949	0.080	0.773	-0.845	0.621	0.986	-0.652	-0.216	0.891	0.735	1.000	0.460	0.504	0.119	0.381	0.519	0.148	-0.252	0.610
bio12	0.271	-0.594	0.135	-0.686	-0.236	0.540	-0.806	-0.170	0.339	-0.041	0.460	1.000	0.976	0.754	0.169	0.974	0.808	0.573	0.937
bio13	0.338	-0.507	0.222	-0.672	-0.139	0.571	-0.767	-0.100	0.381	0.052	0.504	0.976	1.000	0.632	0.353	0.995	0.689	0.471	0.954
bio14	-0.074	-0.715	-0.270	-0.441	-0.505	0.214	-0.641	-0.244	0.028	-0.341	0.119	0.754	0.632	1.000	-0.344	0.602	0.988	0.836	0.513
bio15	0.434	0.222	0.467	-0.165	0.401	0.350	-0.090	0.265	0.343	0.459	0.381	0.169	0.353	-0.344	1.000	0.359	-0.318	-0.306	0.345
bio16	0.351	-0.498	0.246	-0.687	-0.128	0.587	-0.776	-0.115	0.398	0.060	0.519	0.974	0.995	0.602	0.359	1.000	0.664	0.437	0.972
bio17	-0.050	-0.718	-0.236	-0.473	-0.495	0.244	-0.667	-0.245	0.054	-0.328	0.148	0.808	0.689	0.988	-0.318	0.664	1.000	0.833	0.582
bio18	-0.390	-0.693	-0.532	-0.066	-0.687	-0.171	-0.339	0.101	-0.384	-0.539	-0.252	0.573	0.471	0.836	-0.306	0.437	0.833	1.000	0.271
bio19	0.444	-0.416	0.364	-0.753	-0.021	0.670	-0.789	-0.226	0.514	0.142	0.610	0.937	0.954	0.513	0.345	0.972	0.582	0.271	1.000

Correlation table for climate variables for M. foliolosa

	bio1	bio2	bio3	bio4	bio5	bio6	bio7	bio8	bio9	bio10	bio11	bio12	bio13	bio14	bio15	bio16	bio17	bio18	bio19
bio1	1.000	0.355	0.801	-0.451	0.677	0.829	-0.280	0.598	0.737	0.765	0.884	0.368	0.428	0.262	-0.294	0.411	0.264	0.517	0.303
bio2	0.355	1.000	0.435	0.523	0.826	-0.156	0.679	0.774	0.010	0.747	-0.047	-0.232	-0.085	-0.338	0.360	-0.118	-0.329	0.077	-0.303
bio3	0.801	0.435	1.000	-0.483	0.469	0.731	-0.332	0.407	0.752	0.518	0.773	0.418	0.479	0.336	-0.284	0.450	0.342	0.522	0.409
bio4	-0.451	0.523	-0.483	1.000	0.340	-0.857	0.973	0.367	-0.735	0.225	-0.812	-0.634	-0.537	-0.664	0.688	-0.551	-0.664	-0.469	-0.665
bio5	0.677	0.826	0.469	0.340	1.000	0.171	0.507	0.926	0.181	0.985	0.267	-0.127	0.009	-0.258	0.230	-0.019	-0.257	0.162	-0.216
bio6	0.829	-0.156	0.731	-0.857	0.171	1.000	-0.763	0.114	0.860	0.295	0.990	0.571	0.547	0.533	-0.560	0.543	0.532	0.536	0.566
bio7	-0.280	0.679	-0.332	0.973	0.507	-0.763	1.000	0.508	-0.633	0.389	-0.690	-0.582	-0.473	-0.636	0.641	-0.487	-0.634	-0.363	-0.637
bio8	0.598	0.774	0.407	0.367	0.926	0.114	0.508	1.000	0.080	0.917	0.205	-0.298	-0.148	-0.427	0.362	-0.185	-0.424	0.011	-0.381
bio9	0.737	0.010	0.752	-0.735	0.181	0.860	-0.633	0.080	1.000	0.274	0.863	0.636	0.609	0.608	-0.606	0.603	0.616	0.604	0.655
bio10	0.765	0.747	0.518	0.225	0.985	0.295	0.389	0.917	0.274	1.000	0.385	-0.068	0.064	-0.200	0.169	0.035	-0.200	0.205	-0.155
bio11	0.884	-0.047	0.773	-0.812	0.267	0.990	-0.690	0.205	0.863	0.385	1.000	0.552	0.540	0.501	-0.540	0.535	0.501	0.556	0.533
bio12	0.368	-0.232	0.418	-0.634	-0.127	0.571	-0.582	-0.298	0.636	-0.068	0.552	1.000	0.949	0.967	-0.685	0.973	0.975	0.901	0.974
bio13	0.428	-0.085	0.479	-0.537	0.009	0.547	-0.473	-0.148	0.609	0.064	0.540	0.949	1.000	0.865	-0.504	0.992	0.873	0.921	0.898
bio14	0.262	-0.338	0.336	-0.664	-0.258	0.533	-0.636	-0.427	0.608	-0.200	0.501	0.967	0.865	1.000	-0.749	0.898	0.996	0.820	0.965
bio15	-0.294	0.360	-0.284	0.688	0.230	-0.560	0.641	0.362	-0.606	0.169	-0.540	-0.685	-0.504	-0.749	1.000	-0.552	-0.748	-0.543	-0.701
bio16	0.411	-0.118	0.450	-0.551	-0.019	0.543	-0.487	-0.185	0.603	0.035	0.535	0.973	0.992	0.898	-0.552	1.000	0.906	0.935	0.919
bio17	0.264	-0.329	0.342	-0.664	-0.257	0.532	-0.634	-0.424	0.616	-0.200	0.501	0.975	0.873	0.996	-0.748	0.906	1.000	0.829	0.973
bio18	0.517	0.077	0.522	-0.469	0.162	0.536	-0.363	0.011	0.604	0.205	0.556	0.901	0.921	0.820	-0.543	0.935	0.829	1.000	0.799
bio19	0.303	-0.303	0.409	-0.665	-0.216	0.566	-0.637	-0.381	0.655	-0.155	0.533	0.974	0.898	0.965	-0.701	0.919	0.973	0.799	1.000

Correlation table for climate variables for M. fusca

	bio1	bio2	bio3	bio4	bio5	bio6	bio7	bio8	bio9	bio10	bio11	bio12	bio13	bio14	bio15	bio16	bio17	bio18	bio19
bio1	1.000	0.132	0.685	-0.799	0.944	0.973	-0.696	0.513	0.815	0.983	0.991	0.471	0.493	0.079	0.432	0.450	0.116	0.391	0.059
bio2	0.132	1.000	0.708	0.111	0.298	-0.040	0.440	0.252	-0.060	0.185	0.079	-0.076	-0.019	-0.252	0.320	0.019	-0.281	0.051	-0.278
bio3	0.685	0.708	1.000	-0.602	0.673	0.597	-0.312	0.520	0.411	0.650	0.688	0.394	0.472	-0.138	0.648	0.479	-0.125	0.480	-0.222
bio4	-0.799	0.111	-0.602	1.000	-0.577	-0.860	0.929	-0.451	-0.588	-0.678	-0.871	-0.583	-0.636	0.009	-0.583	-0.608	-0.041	-0.580	0.088
bio5	0.944	0.298	0.673	-0.577	1.000	0.884	-0.458	0.466	0.814	0.984	0.900	0.334	0.320	0.143	0.267	0.283	0.161	0.229	0.149
bio6	0.973	-0.040	0.597	-0.860	0.884	1.000	-0.820	0.471	0.841	0.937	0.987	0.528	0.526	0.177	0.378	0.482	0.216	0.429	0.143
bio7	-0.696	0.440	-0.312	0.929	-0.458	-0.820	1.000	-0.325	-0.605	-0.578	-0.776	-0.594	-0.610	-0.161	-0.393	-0.571	-0.214	-0.536	-0.089
bio8	0.513	0.252	0.520	-0.451	0.466	0.471	-0.325	1.000	0.194	0.487	0.513	0.132	0.247	-0.216	0.434	0.232	-0.213	0.306	-0.450
bio9	0.815	-0.060	0.411	-0.588	0.814	0.841	-0.605	0.194	1.000	0.828	0.802	0.450	0.373	0.399	0.043	0.328	0.424	0.244	0.491
bio10	0.983	0.185	0.650	-0.678	0.984	0.937	-0.578	0.487	0.828	1.000	0.951	0.393	0.401	0.109	0.339	0.356	0.137	0.292	0.110
bio11	0.991	0.079	0.688	-0.871	0.900	0.987	-0.776	0.513	0.802	0.951	1.000	0.510	0.534	0.077	0.465	0.493	0.118	0.440	0.044
bio12	0.471	-0.076	0.394	-0.583	0.334	0.528	-0.594	0.132	0.450	0.393	0.510	1.000	0.927	0.510	0.408	0.938	0.555	0.916	0.391
bio13	0.493	-0.019	0.472	-0.636	0.320	0.526	-0.610	0.247	0.373	0.401	0.534	0.927	1.000	0.271	0.647	0.987	0.316	0.938	0.138
bio14	0.079	-0.252	-0.138	0.009	0.143	0.177	-0.161	-0.216	0.399	0.109	0.077	0.510	0.271	1.000	-0.482	0.263	0.987	0.243	0.895
bio15	0.432	0.320	0.648	-0.583	0.267	0.378	-0.393	0.434	0.043	0.339	0.465	0.408	0.647	-0.482	1.000	0.653	-0.452	0.642	-0.564
bio16	0.450	0.019	0.479	-0.608	0.283	0.482	-0.571	0.232	0.328	0.356	0.493	0.938	0.987	0.263	0.653	1.000	0.305	0.969	0.128
bio17	0.116	-0.281	-0.125	-0.041	0.161	0.216	-0.214	-0.213	0.424	0.137	0.118	0.555	0.316	0.987	-0.452	0.305	1.000	0.279	0.899
bio18	0.391	0.051	0.480	-0.580	0.229	0.429	-0.536	0.306	0.244	0.292	0.440	0.916	0.938	0.243	0.642	0.969	0.279	1.000	0.065
bio19	0.059	-0.278	-0.222	0.088	0.149	0.143	-0.089	-0.450	0.491	0.110	0.044	0.391	0.138	0.895	-0.564	0.128	0.899	0.065	1.000

Correlation table for climate variables for M. hieraciifolia

	bio1	bio2	bio3	bio4	bio5	bio6	bio7	bio8	bio9	bio10	bio11	bio12	bio13	bio14	bio15	bio16	bio17	bio18	bio19
bio1	1.000	0.284	0.877	-0.647	0.778	0.927	-0.539	0.705	0.832	0.858	0.946	0.515	0.533	0.436	-0.359	0.532	0.435	0.607	0.444
bio2	0.284	1.000	0.380	0.370	0.712	-0.031	0.516	0.665	0.113	0.602	0.030	-0.171	-0.012	-0.265	0.317	-0.056	-0.257	0.075	-0.218
bio3	0.877	0.380	1.000	-0.677	0.620	0.859	-0.567	0.530	0.847	0.674	0.875	0.579	0.580	0.532	-0.423	0.573	0.537	0.636	0.548
bio4	-0.647	0.370	-0.677	1.000	-0.033	-0.875	0.982	0.012	-0.785	-0.167	-0.857	-0.701	-0.591	-0.724	0.696	-0.613	-0.722	-0.593	-0.695
bio5	0.778	0.712	0.620	-0.033	1.000	0.498	0.103	0.932	0.456	0.985	0.540	0.104	0.215	-0.017	0.085	0.194	-0.016	0.312	0.019
bio6	0.927	-0.031	0.859	-0.875	0.498	1.000	-0.811	0.433	0.886	0.615	0.996	0.648	0.603	0.612	-0.546	0.614	0.610	0.645	0.605
bio7	-0.539	0.516	-0.567	0.982	0.103	-0.811	1.000	0.132	-0.708	-0.042	-0.778	-0.673	-0.547	-0.713	0.683	-0.573	-0.711	-0.530	-0.681
bio8	0.705	0.665	0.530	0.012	0.932	0.433	0.132	1.000	0.351	0.921	0.474	-0.048	0.084	-0.172	0.204	0.057	-0.176	0.210	-0.139
bio9	0.832	0.113	0.847	-0.785	0.456	0.886	-0.708	0.351	1.000	0.548	0.891	0.672	0.631	0.640	-0.604	0.630	0.651	0.645	0.670
bio10	0.858	0.602	0.674	-0.167	0.985	0.615	-0.042	0.921	0.548	1.000	0.650	0.187	0.281	0.070	0.000	0.264	0.070	0.372	0.101
bio11	0.946	0.030	0.875	-0.857	0.540	0.996	-0.778	0.474	0.891	0.650	1.000	0.634	0.599	0.591	-0.531	0.608	0.590	0.647	0.587
bio12	0.515	-0.171	0.579	-0.701	0.104	0.648	-0.673	-0.048	0.672	0.187	0.634	1.000	0.941	0.964	-0.652	0.966	0.971	0.898	0.972
bio13	0.533	-0.012	0.580	-0.591	0.215	0.603	-0.547	0.084	0.631	0.281	0.599	0.941	1.000	0.848	-0.440	0.991	0.855	0.921	0.881
bio14	0.436	-0.265	0.532	-0.724	-0.017	0.612	-0.713	-0.172	0.640	0.070	0.591	0.964	0.848	1.000	-0.744	0.882	0.995	0.831	0.955
bio15	-0.359	0.317	-0.423	0.696	0.085	-0.546	0.683	0.204	-0.604	0.000	-0.531	-0.652	-0.440	-0.744	1.000	-0.488	-0.738	-0.490	-0.686
bio16	0.532	-0.056	0.573	-0.613	0.194	0.614	-0.573	0.057	0.630	0.264	0.608	0.966	0.991	0.882	-0.488	1.000	0.888	0.934	0.905
bio17	0.435	-0.257	0.537	-0.722	-0.016	0.610	-0.711	-0.176	0.651	0.070	0.590	0.971	0.855	0.995	-0.738	0.888	1.000	0.828	0.968
bio18	0.607	0.075	0.636	-0.593	0.312	0.645	-0.530	0.210	0.645	0.372	0.647	0.898	0.921	0.831	-0.490	0.934	0.828	1.000	0.793
bio19	0.444	-0.218	0.548	-0.695	0.019	0.605	-0.681	-0.139	0.670	0.101	0.587	0.972	0.881	0.955	-0.686	0.905	0.968	0.793	1.000

Correlation table for climate variables for M. idahoensis

	bio1	bio2	bio3	bio4	bio5	bio6	bio7	bio8	bio9	bio10	bio11	bio12	bio13	bio14	bio15	bio16	bio17	bio18	bio19
bio1	1.000	0.061	0.390	-0.210	0.788	0.842	-0.179	0.210	0.665	0.920	0.913	-0.012	0.055	-0.566	0.388	0.074	-0.457	-0.637	0.098
bio2	0.061	1.000	0.564	0.476	0.590	-0.321	0.796	0.181	0.079	0.269	-0.144	-0.617	-0.596	-0.584	-0.264	-0.594	-0.623	-0.508	-0.588
bio3	0.390	0.564	1.000	-0.426	0.410	0.383	-0.040	-0.001	0.423	0.235	0.500	0.059	0.077	-0.395	0.132	0.093	-0.254	-0.467	0.128
bio4	-0.210	0.476	-0.426	1.000	0.305	-0.669	0.903	0.313	-0.331	0.188	-0.589	-0.733	-0.706	-0.330	-0.306	-0.721	-0.504	-0.110	-0.763
bio5	0.788	0.590	0.410	0.305	1.000	0.391	0.436	0.257	0.537	0.925	0.528	-0.486	-0.428	-0.769	0.055	-0.412	-0.751	-0.798	-0.387
bio6	0.842	-0.321	0.383	-0.669	0.391	1.000	-0.658	-0.072	0.679	0.579	0.975	0.397	0.426	-0.186	0.429	0.450	-0.023	-0.397	0.498
bio7	-0.179	0.796	-0.040	0.903	0.436	-0.658	1.000	0.280	-0.225	0.190	-0.521	-0.786	-0.766	-0.447	-0.375	-0.777	-0.592	-0.264	-0.803
bio8	0.210	0.181	-0.001	0.313	0.257	-0.072	0.280	1.000	-0.364	0.323	0.037	-0.201	-0.114	-0.390	0.221	-0.138	-0.388	0.036	-0.244
bio9	0.665	0.079	0.423	-0.331	0.537	0.679	-0.225	-0.364	1.000	0.547	0.693	0.108	0.101	-0.228	0.109	0.134	-0.134	-0.599	0.226
bio10	0.920	0.269	0.235	0.188	0.925	0.579	0.190	0.323	0.547	1.000	0.682	-0.313	-0.237	-0.703	0.249	-0.223	-0.663	-0.698	-0.213
bio11	0.913	-0.144	0.500	-0.589	0.528	0.975	-0.521	0.037	0.693	0.682	1.000	0.293	0.335	-0.331	0.442	0.358	-0.168	-0.485	0.396
bio12	-0.012	-0.617	0.059	-0.733	-0.486	0.397	-0.786	-0.201	0.108	-0.313	0.293	1.000	0.985	0.554	0.455	0.987	0.748	0.513	0.984
bio13	0.055	-0.596	0.077	-0.706	-0.428	0.426	-0.766	-0.114	0.101	-0.237	0.335	0.985	1.000	0.437	0.581	0.999	0.644	0.458	0.982
bio14	-0.566	-0.584	-0.395	-0.330	-0.769	-0.186	-0.447	-0.390	-0.228	-0.703	-0.331	0.554	0.437	1.000	-0.282	0.432	0.958	0.785	0.450
bio15	0.388	-0.264	0.132	-0.306	0.055	0.429	-0.375	0.221	0.109	0.249	0.442	0.455	0.581	-0.282	1.000	0.575	-0.110	0.003	0.507
bio16	0.074	-0.594	0.093	-0.721	-0.412	0.450	-0.777	-0.138	0.134	-0.223	0.358	0.987	0.999	0.432	0.575	1.000	0.642	0.433	0.988
bio17	-0.457	-0.623	-0.254	-0.504	-0.751	-0.023	-0.592	-0.388	-0.134	-0.663	-0.168	0.748	0.644	0.958	-0.110	0.642	1.000	0.769	0.660
bio18	-0.637	-0.508	-0.467	-0.110	-0.798	-0.397	-0.264	0.036	-0.599	-0.698	-0.485	0.513	0.458	0.785	0.003	0.433	0.769	1.000	0.363
bio19	0.098	-0.588	0.128	-0.763	-0.387	0.498	-0.803	-0.244	0.226	-0.213	0.396	0.984	0.982	0.450	0.507	0.988	0.660	0.363	1.000

Correlation table for climate variables for M. lyallii

	bio1	bio2	bio3	bio4	bio5	bio6	bio7	bio8	bio9	bio10	bio11	bio12	bio13	bio14	bio15	bio16	bio17	bio18	bio19
bio1	1.000	0.088	0.754	-0.703	0.651	0.889	-0.578	-0.253	0.853	0.743	0.930	0.404	0.364	0.258	-0.166	0.394	0.291	-0.154	0.487
bio2	0.088	1.000	0.355	0.284	0.671	-0.232	0.576	0.138	0.022	0.383	-0.105	-0.566	-0.559	-0.589	0.020	-0.540	-0.583	-0.583	-0.453
bio3	0.754	0.355	1.000	-0.759	0.465	0.758	-0.539	-0.449	0.775	0.353	0.818	0.340	0.263	0.197	-0.311	0.311	0.245	-0.276	0.469
bio4	-0.703	0.284	-0.759	1.000	0.004	-0.922	0.942	0.581	-0.771	-0.053	-0.912	-0.656	-0.545	-0.610	0.459	-0.576	-0.648	-0.127	-0.685
bio5	0.651	0.671	0.465	0.004	1.000	0.287	0.213	0.094	0.455	0.928	0.374	-0.226	-0.196	-0.361	0.117	-0.174	-0.350	-0.549	-0.091
bio6	0.889	-0.232	0.758	-0.922	0.287	1.000	-0.875	-0.471	0.866	0.399	0.987	0.622	0.552	0.511	-0.290	0.581	0.547	0.019	0.678
bio7	-0.578	0.576	-0.539	0.942	0.213	-0.875	1.000	0.528	-0.654	0.061	-0.818	-0.748	-0.662	-0.703	0.355	-0.680	-0.734	-0.297	-0.737
bio8	-0.253	0.138	-0.449	0.581	0.094	-0.471	0.528	1.000	-0.546	0.173	-0.446	-0.355	-0.231	-0.418	0.483	-0.260	-0.437	0.110	-0.434
bio9	0.853	0.022	0.775	-0.771	0.455	0.866	-0.654	-0.546	1.000	0.483	0.884	0.498	0.422	0.380	-0.297	0.459	0.417	-0.176	0.602
bio10	0.743	0.383	0.353	-0.053	0.928	0.399	0.061	0.173	0.483	1.000	0.457	-0.053	0.000	-0.216	0.215	0.011	-0.208	-0.359	0.041
bio11	0.930	-0.105	0.818	-0.912	0.374	0.987	-0.818	-0.446	0.884	0.457	1.000	0.569	0.494	0.459	-0.312	0.525	0.496	-0.029	0.633
bio12	0.404	-0.566	0.340	-0.656	-0.226	0.622	-0.748	-0.355	0.498	-0.053	0.569	1.000	0.968	0.843	-0.123	0.973	0.885	0.572	0.954
bio13	0.364	-0.559	0.263	-0.545	-0.196	0.552	-0.662	-0.231	0.422	0.000	0.494	0.968	1.000	0.741	0.098	0.993	0.780	0.566	0.927
bio14	0.258	-0.589	0.197	-0.610	-0.361	0.511	-0.703	-0.418	0.380	-0.216	0.459	0.843	0.741	1.000	-0.394	0.723	0.988	0.704	0.694
bio15	-0.166	0.020	-0.311	0.459	0.117	-0.290	0.355	0.483	-0.297	0.215	-0.312	-0.123	0.098	-0.394	1.000	0.077	-0.404	0.007	-0.103
bio16	0.394	-0.540	0.311	-0.576	-0.174	0.581	-0.680	-0.260	0.459	0.011	0.525	0.973	0.993	0.723	0.077	1.000	0.769	0.522	0.954
bio17	0.291	-0.583	0.245	-0.648	-0.350	0.547	-0.734	-0.437	0.417	-0.208	0.496	0.885	0.780	0.988	-0.404	0.769	1.000	0.688	0.754
bio18	-0.154	-0.583	-0.276	-0.127	-0.549	0.019	-0.297	0.110	-0.176	-0.359	-0.029	0.572	0.566	0.704	0.007	0.522	0.688	1.000	0.327
bio19	0.487	-0.453	0.469	-0.685	-0.091	0.678	-0.737	-0.434	0.602	0.041	0.633	0.954	0.927	0.694	-0.103	0.954	0.754	0.327	1.000

Correlation table for climate variables for M. melanocentra

	bio1	bio2	bio3	bio4	bio5	bio6	bio7	bio8	bio9	bio10	bio11	bio12	bio13	bio14	bio15	bio16	bio17	bio18	bio19
bio1	1.000	-0.451	0.100	-0.470	0.948	0.979	-0.519	0.946	0.957	0.977	0.984	0.716	0.737	0.408	0.084	0.727	0.475	0.618	0.360
bio2	-0.451	1.000	0.438	0.225	-0.362	-0.554	0.568	-0.338	-0.461	-0.444	-0.446	-0.525	-0.440	-0.632	0.506	-0.452	-0.581	-0.443	-0.453
bio3	0.100	0.438	1.000	-0.751	-0.114	0.146	-0.478	0.079	0.151	-0.074	0.240	0.271	0.317	-0.176	0.492	0.317	-0.063	0.358	-0.127
bio4	-0.470	0.225	-0.751	1.000	-0.180	-0.587	0.921	-0.371	-0.536	-0.273	-0.619	-0.685	-0.683	-0.276	-0.222	-0.689	-0.373	-0.702	-0.215
bio5	0.948	-0.362	-0.114	-0.180	1.000	0.880	-0.234	0.926	0.887	0.990	0.880	0.528	0.566	0.327	0.057	0.549	0.391	0.409	0.330
bio6	0.979	-0.554	0.146	-0.587	0.880	1.000	-0.667	0.902	0.963	0.928	0.990	0.768	0.777	0.480	0.035	0.770	0.555	0.674	0.420
bio7	-0.519	0.568	-0.478	0.921	-0.234	-0.667	1.000	-0.394	-0.580	-0.350	-0.648	-0.745	-0.705	-0.470	0.017	-0.716	-0.522	-0.740	-0.342
bio8	0.946	-0.338	0.079	-0.371	0.926	0.902	-0.394	1.000	0.849	0.944	0.917	0.630	0.660	0.268	0.176	0.652	0.327	0.561	0.204
bio9	0.957	-0.461	0.151	-0.536	0.887	0.963	-0.580	0.849	1.000	0.915	0.960	0.711	0.739	0.475	0.043	0.724	0.578	0.591	0.489
bio10	0.977	-0.444	-0.074	-0.273	0.990	0.928	-0.350	0.944	0.915	1.000	0.924	0.613	0.635	0.382	0.036	0.624	0.430	0.503	0.342
bio11	0.984	-0.446	0.240	-0.619	0.880	0.990	-0.648	0.917	0.960	0.924	1.000	0.771	0.790	0.419	0.122	0.782	0.499	0.687	0.365
bio12	0.716	-0.525	0.271	-0.685	0.528	0.768	-0.745	0.630	0.711	0.613	0.771	1.000	0.970	0.519	0.010	0.983	0.562	0.961	0.384
bio13	0.737	-0.440	0.317	-0.683	0.566	0.777	-0.705	0.660	0.739	0.635	0.790	0.970	1.000	0.399	0.174	0.995	0.500	0.933	0.337
bio14	0.408	-0.632	-0.176	-0.276	0.327	0.480	-0.470	0.268	0.475	0.382	0.419	0.519	0.399	1.000	-0.516	0.410	0.897	0.395	0.829
bio15	0.084	0.506	0.492	-0.222	0.057	0.035	0.017	0.176	0.043	0.036	0.122	0.010	0.174	-0.516	1.000	0.145	-0.402	0.084	-0.406
bio16	0.727	-0.452	0.317	-0.689	0.549	0.770	-0.716	0.652	0.724	0.624	0.782	0.983	0.995	0.410	0.145	1.000	0.489	0.953	0.317
bio17	0.475	-0.581	-0.063	-0.373	0.391	0.555	-0.522	0.327	0.578	0.430	0.499	0.562	0.500	0.897	-0.402	0.489	1.000	0.404	0.948
bio18	0.618	-0.443	0.358	-0.702	0.409	0.674	-0.740	0.561	0.591	0.503	0.687	0.961	0.933	0.395	0.084	0.953	0.404	1.000	0.212
bio19	0.360	-0.453	-0.127	-0.215	0.330	0.420	-0.342	0.204	0.489	0.342	0.365	0.384	0.337	0.829	-0.406	0.317	0.948	0.212	1.000

Correlation table for climate variables for M. micranthidifolia

	bio1	bio2	bio3	bio4	bio5	bio6	bio7	bio8	bio9	bio10	bio11	bio12	bio13	bio14	bio15	bio16	bio17	bio18	bio19
bio1	1.000	0.589	0.775	-0.793	0.941	0.988	-0.724	-0.051	0.790	0.974	0.990	0.373	0.425	0.339	-0.014	0.406	0.355	0.264	0.581
bio2	0.589	1.000	0.887	-0.655	0.556	0.538	-0.337	-0.084	0.556	0.498	0.618	0.300	0.364	0.278	0.018	0.332	0.276	0.278	0.402
bio3	0.775	0.887	1.000	-0.922	0.624	0.779	-0.725	-0.201	0.731	0.637	0.834	0.516	0.574	0.487	-0.016	0.551	0.473	0.458	0.636
bio4	-0.793	-0.655	-0.922	1.000	-0.566	-0.842	0.927	0.252	-0.752	-0.635	-0.871	-0.617	-0.649	-0.597	0.067	-0.639	-0.572	-0.541	-0.724
bio5	0.941	0.556	0.624	-0.566	1.000	0.895	-0.462	0.063	0.665	0.985	0.890	0.156	0.218	0.124	0.035	0.195	0.151	0.056	0.385
bio6	0.988	0.538	0.779	-0.842	0.895	1.000	-0.809	-0.095	0.786	0.943	0.993	0.413	0.463	0.386	-0.022	0.446	0.390	0.300	0.613
bio7	-0.724	-0.337	-0.725	0.927	-0.462	-0.809	1.000	0.270	-0.686	-0.577	-0.803	-0.615	-0.634	-0.603	0.090	-0.629	-0.576	-0.522	-0.711
bio8	-0.051	-0.084	-0.201	0.252	0.063	-0.095	0.270	1.000	-0.348	0.031	-0.101	-0.539	-0.586	-0.402	-0.052	-0.561	-0.403	-0.154	-0.582
bio9	0.790	0.556	0.731	-0.752	0.665	0.786	-0.686	-0.348	1.000	0.716	0.807	0.667	0.705	0.507	-0.005	0.678	0.593	0.437	0.823
bio10	0.974	0.498	0.637	-0.635	0.985	0.943	-0.577	0.031	0.716	1.000	0.932	0.241	0.291	0.212	-0.005	0.270	0.238	0.127	0.464
bio11	0.990	0.618	0.834	-0.871	0.890	0.993	-0.803	-0.101	0.807	0.932	1.000	0.441	0.489	0.412	-0.031	0.471	0.418	0.334	0.635
bio12	0.373	0.300	0.516	-0.617	0.156	0.413	-0.615	-0.539	0.667	0.241	0.441	1.000	0.923	0.883	-0.262	0.941	0.941	0.798	0.949
bio13	0.425	0.364	0.574	-0.649	0.218	0.463	-0.634	-0.586	0.705	0.291	0.489	0.923	1.000	0.741	0.052	0.978	0.803	0.773	0.915
bio14	0.339	0.278	0.487	-0.597	0.124	0.386	-0.603	-0.402	0.507	0.212	0.412	0.883	0.741	1.000	-0.589	0.740	0.968	0.670	0.815
bio15	-0.014	0.018	-0.016	0.067	0.035	-0.022	0.090	-0.052	-0.005	-0.005	-0.031	-0.262	0.052	-0.589	1.000	0.041	-0.524	-0.038	-0.194
bio16	0.406	0.332	0.551	-0.639	0.195	0.446	-0.629	-0.561	0.678	0.270	0.471	0.941	0.978	0.740	0.041	1.000	0.808	0.819	0.915
bio17	0.355	0.276	0.473	-0.572	0.151	0.390	-0.576	-0.403	0.593	0.238	0.418	0.941	0.803	0.968	-0.524	0.808	1.000	0.738	0.875
bio18	0.264	0.278	0.458	-0.541	0.056	0.300	-0.522	-0.154	0.437	0.127	0.334	0.798	0.773	0.670	-0.038	0.819	0.738	1.000	0.669
bio19	0.581	0.402	0.636	-0.724	0.385	0.613	-0.711	-0.582	0.823	0.464	0.635	0.949	0.915	0.815	-0.194	0.915	0.875	0.669	1.000

Correlation table for climate variables for M. nidifica

	bio1	bio2	bio3	bio4	bio5	bio6	bio7	bio8	bio9	bio10	bio11	bio12	bio13	bio14	bio15	bio16	bio17	bio18	bio19
bio1	1.000	0.279	0.580	-0.325	0.829	0.882	-0.165	0.461	0.719	0.933	0.945	-0.093	0.038	-0.703	0.602	0.040	-0.655	-0.732	0.075
bio2	0.279	1.000	0.491	0.323	0.675	-0.059	0.695	0.265	0.242	0.432	0.133	-0.622	-0.560	-0.639	-0.085	-0.559	-0.675	-0.639	-0.541
bio3	0.580	0.491	1.000	-0.639	0.426	0.626	-0.269	0.233	0.525	0.381	0.709	0.061	0.178	-0.625	0.580	0.180	-0.543	-0.647	0.218
bio4	-0.325	0.323	-0.639	1.000	0.167	-0.697	0.900	0.073	-0.368	0.035	-0.614	-0.633	-0.689	0.052	-0.649	-0.692	-0.072	0.111	-0.724
bio5	0.829	0.675	0.426	0.167	1.000	0.508	0.394	0.449	0.612	0.947	0.640	-0.487	-0.380	-0.777	0.238	-0.377	-0.783	-0.809	-0.347
bio6	0.882	-0.059	0.626	-0.697	0.508	1.000	-0.592	0.250	0.721	0.668	0.974	0.282	0.394	-0.480	0.732	0.400	-0.381	-0.549	0.441
bio7	-0.165	0.695	-0.269	0.900	0.394	-0.592	1.000	0.153	-0.197	0.173	-0.441	-0.756	-0.777	-0.215	-0.559	-0.779	-0.327	-0.171	-0.796
bio8	0.461	0.265	0.233	0.073	0.449	0.250	0.153	1.000	-0.048	0.507	0.353	-0.205	-0.104	-0.472	0.244	-0.118	-0.480	-0.273	-0.164
bio9	0.719	0.242	0.525	-0.368	0.612	0.721	-0.197	-0.048	1.000	0.629	0.733	0.028	0.098	-0.455	0.431	0.110	-0.391	-0.636	0.174
bio10	0.933	0.432	0.381	0.035	0.947	0.668	0.173	0.507	0.629	1.000	0.767	-0.342	-0.225	-0.729	0.379	-0.224	-0.723	-0.744	-0.198
bio11	0.945	0.133	0.709	-0.614	0.640	0.974	-0.441	0.353	0.733	0.767	1.000	0.137	0.265	-0.610	0.722	0.268	-0.526	-0.658	0.309
bio12	-0.093	-0.622	0.061	-0.633	-0.487	0.282	-0.756	-0.205	0.028	-0.342	0.137	1.000	0.980	0.470	0.374	0.983	0.616	0.478	0.975
bio13	0.038	-0.560	0.178	-0.689	-0.380	0.394	-0.777	-0.104	0.098	-0.225	0.265	0.980	1.000	0.308	0.527	0.999	0.462	0.344	0.990
bio14	-0.703	-0.639	-0.625	0.052	-0.777	-0.480	-0.215	-0.472	-0.455	-0.729	-0.610	0.470	0.308	1.000	-0.465	0.311	0.974	0.900	0.293
bio15	0.602	-0.085	0.580	-0.649	0.238	0.732	-0.559	0.244	0.431	0.379	0.722	0.374	0.527	-0.465	1.000	0.516	-0.365	-0.367	0.520
bio16	0.040	-0.559	0.180	-0.692	-0.377	0.400	-0.779	-0.118	0.110	-0.224	0.268	0.983	0.999	0.311	0.516	1.000	0.468	0.340	0.993
bio17	-0.655	-0.675	-0.543	-0.072	-0.783	-0.381	-0.327	-0.480	-0.391	-0.723	-0.526	0.616	0.462	0.974	-0.365	0.468	1.000	0.896	0.451
bio18	-0.732	-0.639	-0.647	0.111	-0.809	-0.549	-0.171	-0.273	-0.636	-0.744	-0.658	0.478	0.344	0.900	-0.367	0.340	0.896	1.000	0.284
bio19	0.075	-0.541	0.218	-0.724	-0.347	0.441	-0.796	-0.164	0.174	-0.198	0.309	0.975	0.990	0.293	0.520	0.993	0.451	0.284	1.000

Correlation table for climate variables for M. nivalis

	bio1	bio2	bio3	bio4	bio5	bio6	bio7	bio8	bio9	bio10	bio11	bio12	bio13	bio14	bio15	bio16	bio17	bio18	bio19
bio1	1.000	0.596	0.897	-0.569	0.872	0.922	-0.353	0.675	0.832	0.907	0.954	0.292	0.345	0.170	-0.180	0.328	0.183	0.364	0.312
bio2	0.596	1.000	0.642	0.116	0.834	0.300	0.400	0.711	0.399	0.762	0.397	-0.185	-0.058	-0.297	0.284	-0.092	-0.278	0.000	-0.145
bio3	0.897	0.642	1.000	-0.633	0.743	0.871	-0.410	0.498	0.860	0.752	0.903	0.352	0.396	0.235	-0.232	0.380	0.257	0.349	0.416
bio4	-0.569	0.116	-0.633	1.000	-0.107	-0.828	0.951	0.120	-0.785	-0.174	-0.787	-0.725	-0.641	-0.697	0.708	-0.657	-0.709	-0.608	-0.723
bio5	0.872	0.834	0.743	-0.107	1.000	0.627	0.141	0.864	0.564	0.991	0.692	-0.069	0.045	-0.202	0.194	0.016	-0.192	0.076	-0.034
bio6	0.922	0.300	0.871	-0.828	0.627	1.000	-0.683	0.396	0.896	0.687	0.993	0.508	0.504	0.415	-0.419	0.502	0.426	0.478	0.530
bio7	-0.353	0.400	-0.410	0.951	0.141	-0.683	1.000	0.307	-0.610	0.055	-0.613	-0.710	-0.598	-0.716	0.714	-0.622	-0.721	-0.536	-0.705
bio8	0.675	0.711	0.498	0.120	0.864	0.396	0.307	1.000	0.282	0.866	0.462	-0.278	-0.151	-0.394	0.391	-0.188	-0.390	-0.041	-0.277
bio9	0.832	0.399	0.860	-0.785	0.564	0.896	-0.610	0.282	1.000	0.594	0.905	0.608	0.599	0.525	-0.474	0.596	0.547	0.579	0.630
bio10	0.907	0.762	0.752	-0.174	0.991	0.687	0.055	0.866	0.594	1.000	0.744	-0.027	0.077	-0.158	0.147	0.050	-0.150	0.109	0.003
bio11	0.954	0.397	0.903	-0.787	0.692	0.993	-0.613	0.462	0.905	0.744	1.000	0.475	0.484	0.374	-0.383	0.478	0.387	0.477	0.494
bio12	0.292	-0.185	0.352	-0.725	-0.069	0.508	-0.710	-0.278	0.608	-0.027	0.475	1.000	0.949	0.937	-0.644	0.965	0.952	0.869	0.962
bio13	0.345	-0.058	0.396	-0.641	0.045	0.504	-0.598	-0.151	0.599	0.077	0.484	0.949	1.000	0.804	-0.444	0.994	0.823	0.816	0.941
bio14	0.170	-0.297	0.235	-0.697	-0.202	0.415	-0.716	-0.394	0.525	-0.158	0.374	0.937	0.804	1.000	-0.761	0.827	0.994	0.860	0.850
bio15	-0.180	0.284	-0.232	0.708	0.194	-0.419	0.714	0.391	-0.474	0.147	-0.383	-0.644	-0.444	-0.761	1.000	-0.480	-0.758	-0.600	-0.568
bio16	0.328	-0.092	0.380	-0.657	0.016	0.502	-0.622	-0.188	0.596	0.050	0.478	0.965	0.994	0.827	-0.480	1.000	0.847	0.821	0.958
bio17	0.183	-0.278	0.257	-0.709	-0.192	0.426	-0.721	-0.390	0.547	-0.150	0.387	0.952	0.823	0.994	-0.758	0.847	1.000	0.866	0.873
bio18	0.364	0.000	0.349	-0.608	0.076	0.478	-0.536	-0.041	0.579	0.109	0.477	0.869	0.816	0.860	-0.600	0.821	0.866	1.000	0.730
bio19	0.312	-0.145	0.416	-0.723	-0.034	0.530	-0.705	-0.277	0.630	0.003	0.494	0.962	0.941	0.850	-0.568	0.958	0.873	0.730	1.000

Correlation table for climate variables for M. nudicaulis

	bio1	bio2	bio3	bio4	bio5	bio6	bio7	bio8	bio9	bio10	bio11	bio12	bio13	bio14	bio15	bio16	bio17	bio18	bio19
bio1	1.000	0.468	0.933	-0.437	0.642	0.943	-0.421	0.519	0.380	0.723	0.933	0.711	0.935	0.531	0.374	0.924	0.611	0.872	0.438
bio2	0.468	1.000	0.477	0.513	0.927	0.189	0.542	0.829	0.325	0.895	0.165	-0.100	0.474	-0.279	0.878	0.347	-0.200	0.189	-0.332
bio3	0.933	0.477	1.000	-0.489	0.551	0.915	-0.465	0.461	0.338	0.621	0.908	0.732	0.950	0.574	0.326	0.938	0.649	0.898	0.472
bio4	-0.437	0.513	-0.489	1.000	0.399	-0.697	0.997	0.387	0.017	0.301	-0.722	-0.823	-0.453	-0.864	0.574	-0.571	-0.844	-0.690	-0.807
bio5	0.642	0.927	0.551	0.399	1.000	0.375	0.417	0.840	0.416	0.992	0.343	0.060	0.586	-0.154	0.858	0.478	-0.056	0.313	-0.192
bio6	0.943	0.189	0.915	-0.697	0.375	1.000	-0.686	0.260	0.329	0.470	0.994	0.872	0.909	0.741	0.086	0.942	0.804	0.938	0.664
bio7	-0.421	0.542	-0.465	0.997	0.417	-0.686	1.000	0.403	0.003	0.318	-0.705	-0.807	-0.432	-0.847	0.589	-0.548	-0.832	-0.674	-0.802
bio8	0.519	0.829	0.461	0.387	0.840	0.260	0.403	1.000	0.234	0.838	0.241	-0.087	0.480	-0.308	0.819	0.361	-0.211	0.252	-0.383
bio9	0.380	0.325	0.338	0.017	0.416	0.329	0.003	0.234	1.000	0.434	0.290	0.233	0.401	0.077	0.270	0.353	0.203	0.290	0.251
bio10	0.723	0.895	0.621	0.301	0.992	0.470	0.318	0.838	0.434	1.000	0.440	0.139	0.654	-0.084	0.837	0.554	0.018	0.400	-0.131
bio11	0.933	0.165	0.908	-0.722	0.343	0.994	-0.705	0.241	0.290	0.440	1.000	0.876	0.898	0.754	0.068	0.939	0.808	0.934	0.665
bio12	0.711	-0.100	0.732	-0.823	0.060	0.872	-0.807	-0.087	0.233	0.139	0.876	1.000	0.774	0.951	-0.283	0.857	0.983	0.884	0.918
bio13	0.935	0.474	0.950	-0.453	0.586	0.909	-0.432	0.480	0.401	0.654	0.898	0.774	1.000	0.583	0.357	0.986	0.689	0.938	0.508
bio14	0.531	-0.279	0.574	-0.864	-0.154	0.741	-0.847	-0.308	0.077	-0.084	0.754	0.951	0.583	1.000	-0.494	0.693	0.974	0.747	0.950
bio15	0.374	0.878	0.326	0.574	0.858	0.086	0.589	0.819	0.270	0.837	0.068	-0.283	0.357	-0.494	1.000	0.231	-0.392	0.087	-0.548
bio16	0.924	0.347	0.938	-0.571	0.478	0.942	-0.548	0.361	0.353	0.554	0.939	0.857	0.986	0.693	0.231	1.000	0.781	0.967	0.614
bio17	0.611	-0.200	0.649	-0.844	-0.056	0.804	-0.832	-0.211	0.203	0.018	0.808	0.983	0.689	0.974	-0.392	0.781	1.000	0.826	0.955
bio18	0.872	0.189	0.898	-0.690	0.313	0.938	-0.674	0.252	0.290	0.400	0.934	0.884	0.938	0.747	0.087	0.967	0.826	1.000	0.665
bio19	0.438	-0.332	0.472	-0.807	-0.192	0.664	-0.802	-0.383	0.251	-0.131	0.665	0.918	0.508	0.950	-0.548	0.614	0.955	0.665	1.000

Correlation table for climate variables for M. occidentalis

	bio1	bio2	bio3	bio4	bio5	bio6	bio7	bio8	bio9	bio10	bio11	bio12	bio13	bio14	bio15	bio16	bio17	bio18	bio19
bio1	1.000	0.317	0.667	-0.446	0.797	0.836	-0.250	-0.083	0.749	0.857	0.901	0.149	0.188	-0.078	0.173	0.202	-0.076	-0.482	0.259
bio2	0.317	1.000	0.541	0.254	0.749	-0.056	0.622	0.161	0.167	0.528	0.108	-0.640	-0.589	-0.661	-0.073	-0.582	-0.690	-0.629	-0.546
bio3	0.667	0.541	1.000	-0.637	0.541	0.697	-0.302	-0.321	0.674	0.394	0.773	0.110	0.114	-0.115	-0.057	0.140	-0.081	-0.563	0.254
bio4	-0.446	0.254	-0.637	1.000	0.071	-0.842	0.910	0.597	-0.627	0.075	-0.788	-0.644	-0.570	-0.529	0.164	-0.589	-0.579	0.081	-0.706
bio5	0.797	0.749	0.541	0.071	1.000	0.393	0.354	0.142	0.508	0.945	0.522	-0.378	-0.309	-0.508	0.108	-0.300	-0.536	-0.676	-0.260
bio6	0.836	-0.056	0.697	-0.842	0.393	1.000	-0.721	-0.417	0.818	0.450	0.981	0.507	0.485	0.307	0.018	0.504	0.339	-0.305	0.605
bio7	-0.250	0.622	-0.302	0.910	0.354	-0.721	1.000	0.531	-0.450	0.255	-0.605	-0.800	-0.727	-0.695	0.063	-0.739	-0.748	-0.199	-0.812
bio8	-0.083	0.161	-0.321	0.597	0.142	-0.417	0.531	1.000	-0.510	0.235	-0.350	-0.287	-0.174	-0.395	0.416	-0.195	-0.417	0.192	-0.363
bio9	0.749	0.167	0.674	-0.627	0.508	0.818	-0.450	-0.510	1.000	0.483	0.822	0.281	0.251	0.144	-0.103	0.275	0.163	-0.488	0.411
bio10	0.857	0.528	0.394	0.075	0.945	0.450	0.255	0.235	0.483	1.000	0.555	-0.228	-0.144	-0.403	0.256	-0.140	-0.430	-0.522	-0.137
bio11	0.901	0.108	0.773	-0.788	0.522	0.981	-0.605	-0.350	0.822	0.555	1.000	0.403	0.394	0.197	0.031	0.413	0.221	-0.384	0.510
bio12	0.149	-0.640	0.110	-0.644	-0.378	0.507	-0.800	-0.287	0.281	-0.228	0.403	1.000	0.977	0.761	0.215	0.978	0.834	0.483	0.966
bio13	0.188	-0.589	0.114	-0.570	-0.309	0.485	-0.727	-0.174	0.251	-0.144	0.394	0.977	1.000	0.657	0.393	0.996	0.729	0.466	0.951
bio14	-0.078	-0.661	-0.115	-0.529	-0.508	0.307	-0.695	-0.395	0.144	-0.403	0.197	0.761	0.657	1.000	-0.198	0.635	0.980	0.624	0.627
bio15	0.173	-0.073	-0.057	0.164	0.108	0.018	0.063	0.416	-0.103	0.256	0.031	0.215	0.393	-0.198	1.000	0.387	-0.179	0.199	0.216
bio16	0.202	-0.582	0.140	-0.589	-0.300	0.504	-0.739	-0.195	0.275	-0.140	0.413	0.978	0.996	0.635	0.387	1.000	0.714	0.434	0.966
bio17	-0.076	-0.690	-0.081	-0.579	-0.536	0.339	-0.748	-0.417	0.163	-0.430	0.221	0.834	0.729	0.980	-0.179	0.714	1.000	0.622	0.718
bio18	-0.482	-0.629	-0.563	0.081	-0.676	-0.305	-0.199	0.192	-0.488	-0.522	-0.384	0.483	0.466	0.624	0.199	0.434	0.622	1.000	0.266
bio19	0.259	-0.546	0.254	-0.706	-0.260	0.605	-0.812	-0.363	0.411	-0.137	0.510	0.966	0.951	0.627	0.216	0.966	0.718	0.266	1.000

Correlation table for climate variables for M. odontoloma

	bio1	bio2	bio3	bio4	bio5	bio6	bio7	bio8	bio9	bio10	bio11	bio12	bio13	bio14	bio15	bio16	bio17	bio18	bio19
bio1	1.000	0.337	0.553	-0.210	0.876	0.861	-0.062	0.449	0.610	0.945	0.944	-0.198	-0.045	-0.728	0.550	-0.045	-0.686	-0.664	-0.013
bio2	0.337	1.000	0.495	0.374	0.647	-0.041	0.708	0.437	0.019	0.477	0.177	-0.622	-0.534	-0.611	0.044	-0.542	-0.648	-0.404	-0.562
bio3	0.553	0.495	1.000	-0.594	0.413	0.601	-0.247	0.179	0.463	0.371	0.685	0.032	0.159	-0.548	0.524	0.157	-0.481	-0.521	0.189
bio4	-0.210	0.374	-0.594	1.000	0.204	-0.633	0.912	0.302	-0.490	0.119	-0.518	-0.618	-0.653	-0.052	-0.447	-0.659	-0.156	0.160	-0.723
bio5	0.876	0.647	0.413	0.204	1.000	0.564	0.395	0.499	0.443	0.964	0.703	-0.508	-0.372	-0.793	0.309	-0.373	-0.794	-0.700	-0.350
bio6	0.861	-0.041	0.601	-0.633	0.564	1.000	-0.536	0.122	0.746	0.665	0.966	0.187	0.309	-0.485	0.598	0.315	-0.399	-0.605	0.384
bio7	-0.062	0.708	-0.247	0.912	0.395	-0.536	1.000	0.374	-0.377	0.245	-0.356	-0.727	-0.724	-0.271	-0.349	-0.732	-0.368	-0.042	-0.785
bio8	0.449	0.437	0.179	0.302	0.499	0.122	0.374	1.000	-0.211	0.552	0.287	-0.364	-0.239	-0.512	0.278	-0.257	-0.535	-0.045	-0.379
bio9	0.610	0.019	0.463	-0.490	0.443	0.746	-0.377	-0.211	1.000	0.457	0.700	0.110	0.150	-0.296	0.255	0.166	-0.222	-0.626	0.312
bio10	0.945	0.477	0.371	0.119	0.964	0.665	0.245	0.552	0.457	1.000	0.787	-0.410	-0.267	-0.759	0.402	-0.269	-0.752	-0.631	-0.257
bio11	0.944	0.177	0.685	-0.518	0.703	0.966	-0.356	0.287	0.700	0.787	1.000	0.029	0.174	-0.622	0.625	0.176	-0.551	-0.643	0.227
bio12	-0.198	-0.622	0.032	-0.618	-0.508	0.187	-0.727	-0.364	0.110	-0.410	0.029	1.000	0.969	0.504	0.233	0.974	0.630	0.361	0.960
bio13	-0.045	-0.534	0.159	-0.653	-0.372	0.309	-0.724	-0.239	0.150	-0.267	0.174	0.969	1.000	0.307	0.438	0.998	0.438	0.254	0.962
bio14	-0.728	-0.611	-0.548	-0.052	-0.793	-0.485	-0.271	-0.512	-0.296	-0.759	-0.622	0.504	0.307	1.000	-0.528	0.315	0.976	0.714	0.324
bio15	0.550	0.044	0.524	-0.447	0.309	0.598	-0.349	0.278	0.255	0.402	0.625	0.233	0.438	-0.528	1.000	0.419	-0.465	-0.216	0.333
bio16	-0.045	-0.542	0.157	-0.659	-0.373	0.315	-0.732	-0.257	0.166	-0.269	0.176	0.974	0.998	0.315	0.419	1.000	0.449	0.243	0.970
bio17	-0.686	-0.648	-0.481	-0.156	-0.794	-0.399	-0.368	-0.535	-0.222	-0.752	-0.551	0.630	0.438	0.976	-0.465	0.449	1.000	0.696	0.463
bio18	-0.664	-0.404	-0.521	0.160	-0.700	-0.605	-0.042	-0.045	-0.626	-0.631	-0.643	0.361	0.254	0.714	-0.216	0.243	0.696	1.000	0.101
bio19	-0.013	-0.562	0.189	-0.723	-0.350	0.384	-0.785	-0.379	0.312	-0.257	0.227	0.960	0.962	0.324	0.333	0.970	0.463	0.101	1.000

Correlation table for climate variables for M. oregana

	bio1	bio2	bio3	bio4	bio5	bio6	bio7	bio8	bio9	bio10	bio11	bio12	bio13	bio14	bio15	bio16	bio17	bio18	bio19
bio1	1.000	0.109	0.482	-0.294	0.810	0.855	-0.201	0.383	0.594	0.925	0.938	-0.077	0.052	-0.694	0.610	0.051	-0.637	-0.637	0.079
bio2	0.109	1.000	0.317	0.522	0.554	-0.317	0.787	0.399	-0.144	0.325	-0.094	-0.682	-0.643	-0.513	-0.191	-0.649	-0.582	-0.318	-0.658
bio3	0.482	0.317	1.000	-0.617	0.258	0.523	-0.321	0.219	0.332	0.262	0.623	0.099	0.200	-0.513	0.540	0.194	-0.435	-0.413	0.216
bio4	-0.294	0.522	-0.617	1.000	0.255	-0.702	0.932	0.203	-0.444	0.088	-0.604	-0.667	-0.714	-0.012	-0.606	-0.716	-0.142	0.078	-0.746
bio5	0.810	0.554	0.258	0.255	1.000	0.436	0.386	0.429	0.400	0.948	0.586	-0.511	-0.409	-0.776	0.233	-0.409	-0.786	-0.692	-0.384
bio6	0.855	-0.317	0.523	-0.702	0.436	1.000	-0.661	0.074	0.700	0.614	0.966	0.325	0.431	-0.439	0.717	0.434	-0.327	-0.501	0.478
bio7	-0.201	0.787	-0.321	0.932	0.386	-0.661	1.000	0.282	-0.384	0.161	-0.501	-0.759	-0.782	-0.197	-0.540	-0.786	-0.320	-0.063	-0.810
bio8	0.383	0.399	0.219	0.203	0.429	0.074	0.282	1.000	-0.330	0.479	0.242	-0.256	-0.168	-0.411	0.210	-0.185	-0.430	0.001	-0.281
bio9	0.594	-0.144	0.332	-0.444	0.400	0.700	-0.384	-0.330	1.000	0.443	0.657	0.140	0.176	-0.282	0.325	0.188	-0.202	-0.580	0.302
bio10	0.925	0.325	0.262	0.088	0.948	0.614	0.161	0.479	0.443	1.000	0.740	-0.347	-0.231	-0.735	0.392	-0.232	-0.725	-0.636	-0.216
bio11	0.938	-0.094	0.623	-0.604	0.586	0.966	-0.501	0.242	0.657	0.740	1.000	0.172	0.297	-0.580	0.726	0.297	-0.485	-0.564	0.331
bio12	-0.077	-0.682	0.099	-0.667	-0.511	0.325	-0.759	-0.256	0.140	-0.347	0.172	1.000	0.980	0.472	0.343	0.984	0.625	0.411	0.972
bio13	0.052	-0.643	0.200	-0.714	-0.409	0.431	-0.782	-0.168	0.176	-0.231	0.297	0.980	1.000	0.314	0.499	0.999	0.474	0.308	0.981
bio14	-0.694	-0.513	-0.513	-0.012	-0.776	-0.439	-0.197	-0.411	-0.282	-0.735	-0.580	0.472	0.314	1.000	-0.483	0.321	0.970	0.790	0.310
bio15	0.610	-0.191	0.540	-0.606	0.233	0.717	-0.540	0.210	0.325	0.392	0.726	0.343	0.499	-0.483	1.000	0.485	-0.386	-0.274	0.457
bio16	0.051	-0.649	0.194	-0.716	-0.409	0.434	-0.786	-0.185	0.188	-0.232	0.297	0.984	0.999	0.321	0.485	1.000	0.483	0.302	0.985
bio17	-0.637	-0.582	-0.435	-0.142	-0.786	-0.327	-0.320	-0.430	-0.202	-0.725	-0.485	0.625	0.474	0.970	-0.386	0.483	1.000	0.775	0.475
bio18	-0.637	-0.318	-0.413	0.078	-0.692	-0.501	-0.063	0.001	-0.580	-0.636	-0.564	0.411	0.308	0.790	-0.274	0.302	0.775	1.000	0.196
bio19	0.079	-0.658	0.216	-0.746	-0.384	0.478	-0.810	-0.281	0.302	-0.216	0.331	0.972	0.981	0.310	0.457	0.985	0.475	0.196	1.000

Correlation table for climate variables for M. pallida

	bio1	bio2	bio3	bio4	bio5	bio6	bio7	bio8	bio9	bio10	bio11	bio12	bio13	bio14	bio15	bio16	bio17	bio18	bio19
bio1	1.000	-0.339	0.209	-0.546	0.953	0.986	-0.543	0.951	0.973	0.982	0.989	0.705	0.702	0.325	0.115	0.702	0.342	0.626	0.180
bio2	-0.339	1.000	0.484	0.125	-0.271	-0.439	0.516	-0.233	-0.376	-0.347	-0.320	-0.479	-0.391	-0.623	0.524	-0.401	-0.581	-0.395	-0.423
bio3	0.209	0.484	1.000	-0.783	0.011	0.221	-0.487	0.255	0.158	0.056	0.328	0.244	0.265	-0.330	0.492	0.283	-0.299	0.345	-0.396
bio4	-0.546	0.125	-0.783	1.000	-0.285	-0.618	0.901	-0.543	-0.501	-0.380	-0.664	-0.638	-0.612	-0.034	-0.287	-0.636	-0.061	-0.680	0.156
bio5	0.953	-0.271	0.011	-0.285	1.000	0.907	-0.279	0.908	0.940	0.989	0.902	0.553	0.567	0.318	0.067	0.556	0.347	0.447	0.248
bio6	0.986	-0.439	0.221	-0.618	0.907	1.000	-0.656	0.932	0.964	0.952	0.989	0.746	0.730	0.375	0.060	0.732	0.398	0.670	0.209
bio7	-0.543	0.516	-0.487	0.901	-0.279	-0.656	1.000	-0.500	-0.516	-0.400	-0.642	-0.711	-0.650	-0.287	-0.018	-0.675	-0.285	-0.729	-0.033
bio8	0.951	-0.233	0.255	-0.543	0.908	0.932	-0.500	1.000	0.888	0.930	0.946	0.653	0.668	0.208	0.227	0.665	0.226	0.609	0.052
bio9	0.973	-0.376	0.158	-0.501	0.940	0.964	-0.516	0.888	1.000	0.962	0.957	0.694	0.684	0.397	0.011	0.681	0.442	0.585	0.308
bio10	0.982	-0.347	0.056	-0.380	0.989	0.952	-0.400	0.930	0.962	1.000	0.944	0.634	0.636	0.355	0.062	0.630	0.366	0.538	0.234
bio11	0.989	-0.320	0.328	-0.664	0.902	0.989	-0.642	0.946	0.957	0.944	1.000	0.739	0.732	0.295	0.155	0.736	0.316	0.677	0.132
bio12	0.705	-0.479	0.244	-0.638	0.553	0.746	-0.711	0.653	0.694	0.634	0.739	1.000	0.971	0.359	0.081	0.984	0.400	0.958	0.203
bio13	0.702	-0.391	0.265	-0.612	0.567	0.730	-0.650	0.668	0.684	0.636	0.732	0.971	1.000	0.227	0.241	0.995	0.314	0.940	0.136
bio14	0.325	-0.623	-0.330	-0.034	0.318	0.375	-0.287	0.208	0.397	0.355	0.295	0.359	0.227	1.000	-0.582	0.241	0.886	0.226	0.784
bio15	0.115	0.524	0.492	-0.287	0.067	0.060	-0.018	0.227	0.011	0.062	0.155	0.081	0.241	-0.582	1.000	0.214	-0.508	0.179	-0.514
bio16	0.702	-0.401	0.283	-0.636	0.556	0.732	-0.675	0.665	0.681	0.630	0.736	0.984	0.995	0.241	0.214	1.000	0.306	0.958	0.117
bio17	0.342	-0.581	-0.299	-0.061	0.347	0.398	-0.285	0.226	0.442	0.366	0.316	0.400	0.314	0.886	-0.508	0.306	1.000	0.226	0.932
bio18	0.626	-0.395	0.345	-0.680	0.447	0.670	-0.729	0.609	0.585	0.538	0.677	0.958	0.940	0.226	0.179	0.958	0.226	1.000	0.015
bio19	0.180	-0.423	-0.396	0.156	0.248	0.209	-0.033	0.052	0.308	0.234	0.132	0.203	0.136	0.784	-0.514	0.117	0.932	0.015	1.000

Correlation table for climate variables for M. petiolaris

	bio1	bio2	bio3	bio4	bio5	bio6	bio7	bio8	bio9	bio10	bio11	bio12	bio13	bio14	bio15	bio16	bio17	bio18	bio19
bio1	1.000	0.363	0.633	-0.532	0.949	0.977	-0.411	-0.118	0.547	0.978	0.982	-0.044	0.131	-0.299	0.594	0.161	-0.267	-0.046	0.254
bio2	0.363	1.000	0.659	-0.094	0.489	0.270	0.261	0.004	0.161	0.385	0.338	-0.192	-0.104	-0.274	0.205	-0.121	-0.255	-0.166	-0.055
bio3	0.633	0.659	1.000	-0.789	0.514	0.685	-0.538	-0.279	0.248	0.513	0.723	0.136	0.235	0.004	0.344	0.272	-0.047	0.133	0.316
bio4	-0.532	-0.094	-0.789	1.000	-0.268	-0.678	0.929	0.358	-0.174	-0.346	-0.679	-0.346	-0.390	-0.243	-0.271	-0.462	-0.157	-0.340	-0.460
bio5	0.949	0.489	0.514	-0.268	1.000	0.871	-0.113	-0.003	0.525	0.989	0.882	-0.203	-0.027	-0.437	0.553	-0.018	-0.384	-0.202	0.097
bio6	0.977	0.270	0.685	-0.678	0.871	1.000	-0.587	-0.187	0.495	0.919	0.996	0.030	0.188	-0.198	0.553	0.227	-0.200	0.012	0.314
bio7	-0.411	0.261	-0.538	0.929	-0.113	-0.587	1.000	0.373	-0.135	-0.229	-0.559	-0.395	-0.423	-0.320	-0.207	-0.488	-0.228	-0.356	-0.476
bio8	-0.118	0.004	-0.279	0.358	-0.003	-0.187	0.373	1.000	-0.460	-0.041	-0.181	-0.620	-0.659	-0.506	-0.004	-0.613	-0.497	-0.035	-0.751
bio9	0.547	0.161	0.248	-0.174	0.525	0.495	-0.135	-0.460	1.000	0.554	0.505	0.458	0.538	0.060	0.394	0.543	0.244	0.118	0.680
bio10	0.978	0.385	0.513	-0.346	0.989	0.919	-0.229	-0.041	0.554	1.000	0.924	-0.139	0.041	-0.389	0.579	0.056	-0.336	-0.142	0.163
bio11	0.982	0.338	0.723	-0.679	0.882	0.996	-0.559	-0.181	0.505	0.924	1.000	0.034	0.193	-0.204	0.564	0.233	-0.198	0.029	0.317
bio12	-0.044	-0.192	0.136	-0.346	-0.203	0.030	-0.395	-0.620	0.458	-0.139	0.034	1.000	0.905	0.834	-0.113	0.924	0.920	0.677	0.919
bio13	0.131	-0.104	0.235	-0.390	-0.027	0.188	-0.423	-0.659	0.538	0.041	0.193	0.905	1.000	0.662	0.235	0.977	0.744	0.616	0.906
bio14	-0.299	-0.274	0.004	-0.243	-0.437	-0.198	-0.320	-0.506	0.060	-0.389	-0.204	0.834	0.662	1.000	-0.516	0.658	0.949	0.566	0.651
bio15	0.594	0.205	0.344	-0.271	0.553	0.553	-0.207	-0.004	0.394	0.579	0.564	-0.113	0.235	-0.516	1.000	0.236	-0.433	0.057	0.099
bio16	0.161	-0.121	0.272	-0.462	-0.018	0.227	-0.488	-0.613	0.543	0.056	0.233	0.924	0.977	0.658	0.236	1.000	0.748	0.676	0.917
bio17	-0.267	-0.255	-0.047	-0.157	-0.384	-0.200	-0.228	-0.497	0.244	-0.336	-0.198	0.920	0.744	0.949	-0.433	0.748	1.000	0.648	0.747
bio18	-0.046	-0.166	0.133	-0.340	-0.202	0.012	-0.356	-0.035	0.118	-0.142	0.029	0.677	0.616	0.566	0.057	0.676	0.648	1.000	0.472
bio19	0.254	-0.055	0.316	-0.460	0.097	0.314	-0.476	-0.751	0.680	0.163	0.317	0.919	0.906	0.651	0.099	0.917	0.747	0.472	1.000

Correlation table for climate variables for M. razshivinii

	bio1	bio2	bio3	bio4	bio5	bio6	bio7	bio8	bio9	bio10	bio11	bio12	bio13	bio14	bio15	bio16	bio17	bio18	bio19
bio1	1.000	0.302	0.677	-0.449	0.579	0.792	-0.342	0.340	0.797	0.664	0.850	0.535	0.518	0.444	-0.390	0.551	0.456	0.543	0.513
bio2	0.302	1.000	0.341	0.301	0.686	-0.175	0.526	0.581	0.088	0.517	-0.033	-0.220	-0.179	-0.259	0.095	-0.178	-0.265	-0.066	-0.249
bio3	0.677	0.341	1.000	-0.768	0.062	0.765	-0.606	-0.141	0.639	0.048	0.829	0.661	0.644	0.612	-0.415	0.675	0.620	0.742	0.629
bio4	-0.449	0.301	-0.768	1.000	0.439	-0.853	0.958	0.548	-0.562	0.362	-0.845	-0.758	-0.720	-0.740	0.481	-0.745	-0.749	-0.762	-0.741
bio5	0.579	0.686	0.062	0.439	1.000	0.008	0.547	0.842	0.280	0.967	0.088	-0.155	-0.139	-0.227	0.026	-0.123	-0.228	-0.119	-0.167
bio6	0.792	-0.175	0.765	-0.853	0.008	1.000	-0.833	-0.180	0.763	0.136	0.981	0.772	0.733	0.708	-0.492	0.775	0.722	0.763	0.751
bio7	-0.342	0.526	-0.606	0.958	0.547	-0.833	1.000	0.617	-0.484	0.421	-0.773	-0.732	-0.691	-0.719	0.427	-0.717	-0.731	-0.705	-0.721
bio8	0.340	0.581	-0.141	0.548	0.842	-0.180	0.617	1.000	0.025	0.803	-0.116	-0.363	-0.334	-0.414	0.170	-0.319	-0.416	-0.230	-0.413
bio9	0.797	0.088	0.639	-0.562	0.280	0.763	-0.484	0.025	1.000	0.369	0.792	0.576	0.562	0.498	-0.406	0.577	0.520	0.538	0.581
bio10	0.664	0.517	0.048	0.362	0.967	0.136	0.421	0.803	0.369	1.000	0.191	-0.058	-0.048	-0.137	-0.020	-0.032	-0.133	-0.061	-0.065
bio11	0.850	-0.033	0.829	-0.845	0.088	0.981	-0.773	-0.116	0.792	0.191	1.000	0.763	0.728	0.698	-0.514	0.766	0.710	0.767	0.741
bio12	0.535	-0.220	0.661	-0.758	-0.155	0.772	-0.732	-0.363	0.576	-0.058	0.763	1.000	0.968	0.980	-0.572	0.983	0.989	0.900	0.987
bio13	0.518	-0.179	0.644	-0.720	-0.139	0.733	-0.691	-0.334	0.562	-0.048	0.728	0.968	1.000	0.921	-0.416	0.991	0.939	0.904	0.941
bio14	0.444	-0.259	0.612	-0.740	-0.227	0.708	-0.719	-0.414	0.498	-0.137	0.698	0.980	0.921	1.000	-0.638	0.941	0.995	0.882	0.966
bio15	-0.390	0.095	-0.415	0.481	0.026	-0.492	0.427	0.170	-0.406	-0.020	-0.514	-0.572	-0.416	-0.638	1.000	-0.457	-0.622	-0.494	-0.583
bio16	0.551	-0.178	0.675	-0.745	-0.123	0.775	-0.717	-0.319	0.577	-0.032	0.766	0.983	0.991	0.941	-0.457	1.000	0.954	0.922	0.956
bio17	0.456	-0.265	0.620	-0.749	-0.228	0.722	-0.731	-0.416	0.520	-0.133	0.710	0.989	0.939	0.995	-0.622	0.954	1.000	0.883	0.977
bio18	0.543	-0.066	0.742	-0.762	-0.119	0.763	-0.705	-0.230	0.538	-0.061	0.767	0.900	0.904	0.882	-0.494	0.922	0.883	1.000	0.826
bio19	0.513	-0.249	0.629	-0.741	-0.167	0.751	-0.721	-0.413	0.581	-0.065	0.741	0.987	0.941	0.966	-0.583	0.956	0.977	0.826	1.000

Correlation table for climate variables for M. reflexa

	bio1	bio2	bio3	bio4	bio5	bio6	bio7	bio8	bio9	bio10	bio11	bio12	bio13	bio14	bio15	bio16	bio17	bio18	bio19
bio1	1.000	0.229	0.740	-0.630	0.398	0.822	-0.514	0.071	0.823	0.500	0.883	0.622	0.586	0.543	-0.449	0.620	0.562	0.580	0.624
bio2	0.229	1.000	0.452	0.198	0.652	-0.221	0.470	0.515	0.140	0.421	-0.056	-0.203	-0.172	-0.250	0.159	-0.158	-0.242	0.018	-0.234
bio3	0.740	0.452	1.000	-0.767	0.063	0.706	-0.563	-0.149	0.731	0.007	0.802	0.636	0.604	0.579	-0.396	0.640	0.601	0.733	0.614
bio4	-0.630	0.198	-0.767	1.000	0.428	-0.911	0.949	0.533	-0.693	0.347	-0.913	-0.774	-0.718	-0.744	0.541	-0.746	-0.766	-0.733	-0.777
bio5	0.398	0.652	0.063	0.428	1.000	-0.149	0.561	0.714	0.136	0.946	-0.055	-0.207	-0.191	-0.264	0.095	-0.177	-0.270	-0.171	-0.210
bio6	0.822	-0.221	0.706	-0.911	-0.149	1.000	-0.902	-0.352	0.771	0.009	0.981	0.804	0.751	0.755	-0.551	0.786	0.772	0.728	0.807
bio7	-0.514	0.470	-0.563	0.949	0.561	-0.902	1.000	0.607	-0.586	0.406	-0.845	-0.764	-0.712	-0.747	0.502	-0.735	-0.764	-0.684	-0.767
bio8	0.071	0.515	-0.149	0.533	0.714	-0.352	0.607	1.000	-0.168	0.655	-0.288	-0.439	-0.381	-0.479	0.370	-0.377	-0.480	-0.253	-0.491
bio9	0.823	0.140	0.731	-0.693	0.136	0.771	-0.586	-0.168	1.000	0.226	0.819	0.647	0.621	0.582	-0.470	0.638	0.610	0.599	0.660
bio10	0.500	0.421	0.007	0.347	0.946	0.009	0.406	0.655	0.226	1.000	0.063	-0.079	-0.064	-0.137	0.025	-0.054	-0.141	-0.101	-0.080
bio11	0.883	-0.056	0.802	-0.913	-0.055	0.981	-0.845	-0.288	0.819	0.063	1.000	0.793	0.738	0.737	-0.564	0.774	0.756	0.737	0.794
bio12	0.622	-0.203	0.636	-0.774	-0.207	0.804	-0.764	-0.439	0.647	-0.079	0.793	1.000	0.968	0.981	-0.527	0.983	0.989	0.884	0.987
bio13	0.586	-0.172	0.604	-0.718	-0.191	0.751	-0.712	-0.381	0.621	-0.064	0.738	0.968	1.000	0.924	-0.344	0.993	0.936	0.885	0.944
bio14	0.543	-0.250	0.579	-0.744	-0.264	0.755	-0.747	-0.479	0.582	-0.137	0.737	0.981	0.924	1.000	-0.578	0.945	0.995	0.870	0.963
bio15	-0.449	0.159	-0.396	0.541	0.095	-0.551	0.502	0.370	-0.470	0.025	-0.564	-0.527	-0.344	-0.578	1.000	-0.395	-0.579	-0.371	-0.564
bio16	0.620	-0.158	0.640	-0.746	-0.177	0.786	-0.735	-0.377	0.638	-0.054	0.774	0.983	0.993	0.945	-0.395	1.000	0.955	0.908	0.958
bio17	0.562	-0.242	0.601	-0.766	-0.270	0.772	-0.764	-0.480	0.610	-0.141	0.756	0.989	0.936	0.995	-0.579	0.955	1.000	0.872	0.973
bio18	0.580	0.018	0.733	-0.733	-0.171	0.728	-0.684	-0.253	0.599	-0.101	0.737	0.884	0.885	0.870	-0.371	0.908	0.872	1.000	0.808
bio19	0.624	-0.234	0.614	-0.777	-0.210	0.807	-0.767	-0.491	0.660	-0.080	0.794	0.987	0.944	0.963	-0.564	0.958	0.973	0.808	1.000

Correlation table for climate variables for M. rhomboidea

	bio1	bio2	bio3	bio4	bio5	bio6	bio7	bio8	bio9	bio10	bio11	bio12	bio13	bio14	bio15	bio16	bio17	bio18	bio19
bio1	1.000	0.522	0.489	0.040	0.946	0.881	0.245	0.516	0.465	0.974	0.955	-0.552	-0.310	-0.797	0.414	-0.318	-0.784	-0.581	-0.277
bio2	0.522	1.000	0.740	0.167	0.658	0.246	0.650	0.432	0.110	0.550	0.457	-0.617	-0.475	-0.608	0.137	-0.493	-0.609	-0.439	-0.479
bio3	0.489	0.740	1.000	-0.508	0.445	0.518	-0.021	0.181	0.355	0.366	0.623	-0.248	-0.117	-0.468	0.240	-0.130	-0.444	-0.440	-0.020
bio4	0.040	0.167	-0.508	1.000	0.249	-0.362	0.840	0.384	-0.367	0.260	-0.253	-0.451	-0.397	-0.199	-0.042	-0.403	-0.237	0.078	-0.594
bio5	0.946	0.658	0.445	0.249	1.000	0.748	0.498	0.502	0.400	0.975	0.846	-0.680	-0.471	-0.810	0.288	-0.476	-0.805	-0.662	-0.405
bio6	0.881	0.246	0.518	-0.362	0.748	1.000	-0.203	0.220	0.626	0.774	0.962	-0.272	-0.104	-0.597	0.343	-0.099	-0.567	-0.607	0.064
bio7	0.245	0.650	-0.021	0.840	0.498	-0.203	1.000	0.453	-0.228	0.428	-0.008	-0.647	-0.559	-0.416	-0.022	-0.573	-0.446	-0.184	-0.682
bio8	0.516	0.432	0.181	0.384	0.502	0.220	0.453	1.000	-0.229	0.578	0.384	-0.545	-0.267	-0.613	0.414	-0.311	-0.631	0.028	-0.637
bio9	0.465	0.110	0.355	-0.367	0.400	0.626	-0.228	-0.229	1.000	0.365	0.554	-0.013	-0.062	-0.170	-0.060	-0.033	-0.117	-0.547	0.343
bio10	0.974	0.550	0.366	0.260	0.975	0.774	0.428	0.578	0.365	1.000	0.868	-0.641	-0.402	-0.814	0.375	-0.410	-0.810	-0.558	-0.407
bio11	0.955	0.457	0.623	-0.253	0.846	0.962	-0.008	0.384	0.554	0.868	1.000	-0.407	-0.193	-0.715	0.403	-0.197	-0.691	-0.595	-0.099
bio12	-0.552	-0.617	-0.248	-0.451	-0.680	-0.272	-0.647	-0.545	-0.013	-0.641	-0.407	1.000	0.882	0.685	-0.004	0.903	0.716	0.519	0.855
bio13	-0.310	-0.475	-0.117	-0.397	-0.471	-0.104	-0.559	-0.267	-0.062	-0.402	-0.193	0.882	1.000	0.325	0.421	0.993	0.348	0.501	0.755
bio14	-0.797	-0.608	-0.468	-0.199	-0.810	-0.597	-0.416	-0.613	-0.170	-0.814	-0.715	0.685	0.325	1.000	-0.581	0.351	0.988	0.526	0.457
bio15	0.414	0.137	0.240	-0.042	0.288	0.343	-0.022	0.414	-0.060	0.375	0.403	-0.004	0.421	-0.581	1.000	0.387	-0.589	0.131	-0.032
bio16	-0.318	-0.493	-0.130	-0.403	-0.476	-0.099	-0.573	-0.311	-0.033	-0.410	-0.197	0.903	0.993	0.351	0.387	1.000	0.376	0.489	0.786
bio17	-0.784	-0.609	-0.444	-0.237	-0.805	-0.567	-0.446	-0.631	-0.117	-0.810	-0.691	0.716	0.348	0.988	-0.589	0.376	1.000	0.508	0.505
bio18	-0.581	-0.439	-0.440	0.078	-0.662	-0.607	-0.184	0.028	-0.547	-0.558	-0.595	0.519	0.501	0.526	0.131	0.489	0.508	1.000	0.030
bio19	-0.277	-0.479	-0.020	-0.594	-0.405	0.064	-0.682	-0.637	0.343	-0.407	-0.099	0.855	0.755	0.457	-0.032	0.786	0.505	0.030	1.000

Correlation table for climate variables for M. rufidula

	bio1	bio2	bio3	bio4	bio5	bio6	bio7	bio8	bio9	bio10	bio11	bio12	bio13	bio14	bio15	bio16	bio17	bio18	bio19
bio1	1.000	0.331	0.614	-0.310	0.771	0.866	-0.129	0.615	0.810	0.902	0.918	0.146	0.234	-0.503	0.625	0.230	-0.401	-0.482	0.238
bio2	0.331	1.000	0.540	0.321	0.756	-0.038	0.696	0.109	0.352	0.504	0.147	-0.547	-0.455	-0.825	0.126	-0.460	-0.833	-0.822	-0.445
bio3	0.614	0.540	1.000	-0.600	0.429	0.636	-0.215	0.438	0.545	0.376	0.744	0.255	0.350	-0.525	0.705	0.350	-0.384	-0.454	0.360
bio4	-0.310	0.321	-0.600	1.000	0.278	-0.707	0.899	-0.336	-0.265	0.129	-0.658	-0.803	-0.814	-0.246	-0.659	-0.820	-0.405	-0.302	-0.814
bio5	0.771	0.756	0.429	0.278	1.000	0.384	0.518	0.348	0.684	0.938	0.504	-0.401	-0.303	-0.780	0.275	-0.310	-0.765	-0.794	-0.296
bio6	0.866	-0.038	0.636	-0.707	0.384	1.000	-0.591	0.574	0.730	0.584	0.977	0.548	0.608	-0.151	0.760	0.608	0.000	-0.123	0.609
bio7	-0.129	0.696	-0.215	0.899	0.518	-0.591	1.000	-0.228	-0.079	0.278	-0.465	-0.858	-0.828	-0.542	-0.464	-0.835	-0.668	-0.581	-0.824
bio8	0.615	0.109	0.438	-0.336	0.348	0.574	-0.228	1.000	0.268	0.480	0.615	0.200	0.249	-0.314	0.405	0.249	-0.233	-0.187	0.247
bio9	0.810	0.352	0.545	-0.265	0.684	0.730	-0.079	0.268	1.000	0.736	0.763	0.129	0.209	-0.389	0.623	0.208	-0.307	-0.474	0.213
bio10	0.902	0.504	0.376	0.129	0.938	0.584	0.278	0.480	0.736	1.000	0.661	-0.214	-0.125	-0.642	0.358	-0.132	-0.607	-0.648	-0.121
bio11	0.918	0.147	0.744	-0.658	0.504	0.977	-0.465	0.615	0.763	0.661	1.000	0.444	0.521	-0.306	0.778	0.520	-0.159	-0.270	0.524
bio12	0.146	-0.547	0.255	-0.803	-0.401	0.548	-0.858	0.200	0.129	-0.214	0.444	1.000	0.987	0.506	0.565	0.989	0.685	0.614	0.986
bio13	0.234	-0.455	0.350	-0.814	-0.303	0.608	-0.828	0.249	0.209	-0.125	0.521	0.987	1.000	0.379	0.670	0.999	0.573	0.493	0.999
bio14	-0.503	-0.825	-0.525	-0.246	-0.780	-0.151	-0.542	-0.314	-0.389	-0.642	-0.306	0.506	0.379	1.000	-0.281	0.384	0.963	0.946	0.369
bio15	0.625	0.126	0.705	-0.659	0.275	0.760	-0.464	0.405	0.623	0.358	0.778	0.565	0.670	-0.281	1.000	0.666	-0.104	-0.233	0.673
bio16	0.230	-0.460	0.350	-0.820	-0.310	0.608	-0.835	0.249	0.208	-0.132	0.520	0.989	0.999	0.384	0.666	1.000	0.579	0.498	0.999
bio17	-0.401	-0.833	-0.384	-0.405	-0.765	0.000	-0.668	-0.233	-0.307	-0.607	-0.159	0.685	0.573	0.963	-0.104	0.579	1.000	0.964	0.565
bio18	-0.482	-0.822	-0.454	-0.302	-0.794	-0.123	-0.581	-0.187	-0.474	-0.648	-0.270	0.614	0.493	0.946	-0.233	0.498	0.964	1.000	0.485
bio19	0.238	-0.445	0.360	-0.814	-0.296	0.609	-0.824	0.247	0.213	-0.121	0.524	0.986	0.999	0.369	0.673	0.999	0.565	0.485	1.000

Correlation table for climate variables for M. spicata

	bio1	bio2	bio3	bio4	bio5	bio6	bio7	bio8	bio9	bio10	bio11	bio12	bio13	bio14	bio15	bio16	bio17	bio18	bio19
bio1	1.000	-0.088	0.528	-0.530	0.371	0.810	-0.481	0.469	0.637	0.596	0.853	0.540	0.616	0.438	-0.305	0.621	0.419	0.580	0.438
bio2	-0.088	1.000	0.435	0.595	0.737	-0.549	0.770	0.525	-0.253	0.434	-0.440	-0.172	-0.260	-0.204	0.044	-0.195	-0.210	-0.033	-0.157
bio3	0.528	0.435	1.000	-0.449	0.235	0.419	-0.229	0.129	0.286	0.132	0.523	0.629	0.541	0.558	-0.443	0.627	0.558	0.724	0.575
bio4	-0.530	0.595	-0.449	1.000	0.550	-0.900	0.967	0.417	-0.480	0.360	-0.892	-0.687	-0.708	-0.652	0.368	-0.722	-0.653	-0.655	-0.615
bio5	0.371	0.737	0.235	0.550	1.000	-0.222	0.626	0.899	0.065	0.921	-0.147	-0.161	-0.155	-0.231	0.038	-0.133	-0.249	-0.060	-0.179
bio6	0.810	-0.549	0.419	-0.900	-0.222	1.000	-0.899	-0.063	0.629	0.053	0.989	0.659	0.728	0.592	-0.362	0.725	0.586	0.641	0.566
bio7	-0.481	0.770	-0.229	0.967	0.626	-0.899	1.000	0.453	-0.473	0.371	-0.857	-0.599	-0.652	-0.577	0.307	-0.640	-0.580	-0.539	-0.533
bio8	0.469	0.525	0.129	0.417	0.899	-0.063	0.453	1.000	0.093	0.904	-0.007	-0.249	-0.167	-0.330	0.168	-0.170	-0.359	-0.105	-0.309
bio9	0.637	-0.253	0.286	-0.480	0.065	0.629	-0.473	0.093	1.000	0.260	0.635	0.449	0.465	0.397	-0.354	0.468	0.402	0.398	0.434
bio10	0.596	0.434	0.132	0.360	0.921	0.053	0.371	0.904	0.260	1.000	0.099	-0.059	0.006	-0.138	0.000	-0.004	-0.159	0.008	-0.101
bio11	0.853	-0.440	0.523	-0.892	-0.147	0.989	-0.857	-0.007	0.635	0.099	1.000	0.693	0.749	0.618	-0.381	0.759	0.609	0.690	0.596
bio12	0.540	-0.172	0.629	-0.687	-0.161	0.659	-0.599	-0.249	0.449	-0.059	0.693	1.000	0.912	0.976	-0.615	0.966	0.980	0.914	0.973
bio13	0.616	-0.260	0.541	-0.708	-0.155	0.728	-0.652	-0.167	0.465	0.006	0.749	0.912	1.000	0.859	-0.370	0.977	0.857	0.925	0.816
bio14	0.438	-0.204	0.558	-0.652	-0.231	0.592	-0.577	-0.330	0.397	-0.138	0.618	0.976	0.859	1.000	-0.643	0.914	0.993	0.862	0.971
bio15	-0.305	0.044	-0.443	0.368	0.038	-0.362	0.307	0.168	-0.354	0.000	-0.381	-0.615	-0.370	-0.643	1.000	-0.456	-0.662	-0.465	-0.674
bio16	0.621	-0.195	0.627	-0.722	-0.133	0.725	-0.640	-0.170	0.468	-0.004	0.759	0.966	0.977	0.914	-0.456	1.000	0.911	0.954	0.891
bio17	0.419	-0.210	0.558	-0.653	-0.249	0.586	-0.580	-0.359	0.402	-0.159	0.609	0.980	0.857	0.993	-0.662	0.911	1.000	0.853	0.984
bio18	0.580	-0.033	0.724	-0.655	-0.060	0.641	-0.539	-0.105	0.398	0.008	0.690	0.914	0.925	0.862	-0.465	0.954	0.853	1.000	0.815
bio19	0.438	-0.157	0.575	-0.615	-0.179	0.566	-0.533	-0.309	0.434	-0.101	0.596	0.973	0.816	0.971	-0.674	0.891	0.984	0.815	1.000

Correlation table for climate variables for M. stellaris

	bio1	bio2	bio3	bio4	bio5	bio6	bio7	bio8	bio9	bio10	bio11	bio12	bio13	bio14	bio15	bio16	bio17	bio18	bio19
bio1	1.000	0.724	0.863	-0.410	0.947	0.950	-0.123	0.761	0.765	0.968	0.973	-0.396	-0.319	-0.488	0.297	-0.338	-0.475	-0.482	-0.256
bio2	0.724	1.000	0.613	0.146	0.871	0.523	0.470	0.741	0.452	0.821	0.593	-0.632	-0.546	-0.661	0.417	-0.572	-0.660	-0.544	-0.552
bio3	0.863	0.613	1.000	-0.670	0.731	0.913	-0.391	0.494	0.804	0.751	0.919	-0.197	-0.174	-0.275	0.100	-0.188	-0.250	-0.395	-0.022
bio4	-0.410	0.146	-0.670	1.000	-0.109	-0.666	0.938	0.103	-0.641	-0.171	-0.608	-0.397	-0.345	-0.301	0.254	-0.350	-0.343	-0.032	-0.534
bio5	0.947	0.871	0.731	-0.109	1.000	0.807	0.198	0.838	0.645	0.994	0.852	-0.567	-0.468	-0.640	0.419	-0.491	-0.637	-0.567	-0.450
bio6	0.950	0.523	0.913	-0.666	0.807	1.000	-0.420	0.576	0.840	0.846	0.995	-0.185	-0.142	-0.290	0.149	-0.154	-0.267	-0.387	-0.024
bio7	-0.123	0.470	-0.391	0.938	0.198	-0.420	1.000	0.331	-0.403	0.123	-0.342	-0.564	-0.483	-0.503	0.396	-0.498	-0.537	-0.229	-0.651
bio8	0.761	0.741	0.494	0.103	0.838	0.576	0.331	1.000	0.250	0.842	0.626	-0.655	-0.540	-0.661	0.413	-0.565	-0.687	-0.351	-0.649
bio9	0.765	0.452	0.804	-0.641	0.645	0.840	-0.403	0.250	1.000	0.661	0.837	-0.030	-0.007	-0.189	0.142	-0.018	-0.131	-0.473	0.196
bio10	0.968	0.821	0.751	-0.171	0.994	0.846	0.123	0.842	0.661	1.000	0.886	-0.538	-0.440	-0.615	0.398	-0.462	-0.612	-0.545	-0.419
bio11	0.973	0.593	0.919	-0.608	0.852	0.995	-0.342	0.626	0.837	0.886	1.000	-0.247	-0.193	-0.353	0.201	-0.208	-0.331	-0.428	-0.085
bio12	-0.396	-0.632	-0.197	-0.397	-0.567	-0.185	-0.564	-0.655	-0.030	-0.538	-0.247	1.000	0.956	0.913	-0.411	0.965	0.940	0.747	0.937
bio13	-0.319	-0.546	-0.174	-0.345	-0.468	-0.142	-0.483	-0.540	-0.007	-0.440	-0.193	0.956	1.000	0.794	-0.173	0.994	0.822	0.724	0.903
bio14	-0.488	-0.661	-0.275	-0.301	-0.640	-0.290	-0.503	-0.661	-0.189	-0.615	-0.353	0.913	0.794	1.000	-0.671	0.808	0.988	0.799	0.788
bio15	0.297	0.417	0.100	0.254	0.419	0.149	0.396	0.413	0.142	0.398	0.201	-0.411	-0.173	-0.671	1.000	-0.191	-0.660	-0.398	-0.319
bio16	-0.338	-0.572	-0.188	-0.350	-0.491	-0.154	-0.498	-0.565	-0.018	-0.462	-0.208	0.965	0.994	0.808	-0.191	1.000	0.835	0.732	0.909
bio17	-0.475	-0.660	-0.250	-0.343	-0.637	-0.267	-0.537	-0.687	-0.131	-0.612	-0.331	0.940	0.822	0.988	-0.660	0.835	1.000	0.775	0.833
bio18	-0.482	-0.544	-0.395	-0.032	-0.567	-0.387	-0.229	-0.351	-0.473	-0.545	-0.428	0.747	0.724	0.799	-0.398	0.732	0.775	1.000	0.501
bio19	-0.256	-0.552	-0.022	-0.534	-0.450	-0.024	-0.651	-0.649	0.196	-0.419	-0.085	0.937	0.903	0.788	-0.319	0.909	0.833	0.501	1.000

Correlation table for climate variables for M. tolmiei

	bio1	bio2	bio3	bio4	bio5	bio6	bio7	bio8	bio9	bio10	bio11	bio12	bio13	bio14	bio15	bio16	bio17	bio18	bio19
bio1	1.000	0.419	0.675	-0.327	0.840	0.873	-0.044	0.381	0.866	0.940	0.945	-0.060	0.046	-0.356	0.638	0.050	-0.352	-0.470	0.091
bio2	0.419	1.000	0.664	0.364	0.797	0.036	0.761	0.132	0.423	0.587	0.238	-0.702	-0.628	-0.778	0.097	-0.619	-0.792	-0.798	-0.562
bio3	0.675	0.664	1.000	-0.421	0.651	0.603	0.040	0.181	0.674	0.572	0.716	-0.141	-0.026	-0.527	0.608	-0.005	-0.504	-0.608	0.068
bio4	-0.327	0.364	-0.421	1.000	0.150	-0.717	0.877	0.001	-0.368	0.012	-0.614	-0.712	-0.750	-0.396	-0.571	-0.759	-0.433	-0.287	-0.769
bio5	0.840	0.797	0.651	0.150	1.000	0.507	0.486	0.296	0.761	0.950	0.657	-0.494	-0.396	-0.668	0.364	-0.390	-0.678	-0.748	-0.337
bio6	0.873	0.036	0.603	-0.717	0.507	1.000	-0.506	0.245	0.814	0.667	0.973	0.352	0.441	-0.008	0.725	0.448	0.014	-0.152	0.479
bio7	-0.044	0.761	0.040	0.877	0.486	-0.506	1.000	0.048	-0.064	0.274	-0.330	-0.851	-0.844	-0.661	-0.371	-0.844	-0.693	-0.594	-0.823
bio8	0.381	0.132	0.181	0.001	0.296	0.245	0.048	1.000	0.079	0.390	0.307	-0.051	-0.009	-0.178	0.216	-0.015	-0.176	-0.093	-0.031
bio9	0.866	0.423	0.674	-0.368	0.761	0.814	-0.064	0.079	1.000	0.795	0.857	0.005	0.102	-0.276	0.613	0.109	-0.268	-0.447	0.154
bio10	0.940	0.587	0.572	0.012	0.950	0.667	0.274	0.390	0.795	1.000	0.781	-0.324	-0.226	-0.522	0.462	-0.225	-0.532	-0.609	-0.184
bio11	0.945	0.238	0.716	-0.614	0.657	0.973	-0.330	0.307	0.857	0.781	1.000	0.187	0.289	-0.168	0.726	0.295	-0.153	-0.305	0.335
bio12	-0.060	-0.702	-0.141	-0.712	-0.494	0.352	-0.851	-0.051	0.005	-0.324	0.187	1.000	0.985	0.765	0.252	0.981	0.824	0.739	0.952
bio13	0.046	-0.628	-0.026	-0.750	-0.396	0.441	-0.844	-0.009	0.102	-0.226	0.289	0.985	1.000	0.671	0.385	0.995	0.735	0.637	0.976
bio14	-0.356	-0.778	-0.527	-0.396	-0.668	-0.008	-0.661	-0.178	-0.276	-0.522	-0.168	0.765	0.671	1.000	-0.250	0.634	0.989	0.948	0.545
bio15	0.638	0.097	0.608	-0.571	0.364	0.725	-0.371	0.216	0.613	0.462	0.726	0.252	0.385	-0.250	1.000	0.400	-0.212	-0.311	0.457
bio16	0.050	-0.619	-0.005	-0.759	-0.390	0.448	-0.844	-0.015	0.109	-0.225	0.295	0.981	0.995	0.634	0.400	1.000	0.704	0.602	0.990
bio17	-0.352	-0.792	-0.504	-0.433	-0.678	0.014	-0.693	-0.176	-0.268	-0.532	-0.153	0.824	0.735	0.989	-0.212	0.704	1.000	0.957	0.621
bio18	-0.470	-0.798	-0.608	-0.287	-0.748	-0.152	-0.594	-0.093	-0.447	-0.609	-0.305	0.739	0.637	0.948	-0.311	0.602	0.957	1.000	0.509
bio19	0.091	-0.562	0.068	-0.769	-0.337	0.479	-0.823	-0.031	0.154	-0.184	0.335	0.952	0.976	0.545	0.457	0.990	0.621	0.509	1.000

Correlation table for soil variables for M. apetala

	bulk	clay	sand	silt	ph	organic
bulk	1.000	0.663	-0.543	0.156	0.879	-0.922
clay	0.663	1.000	-0.709	0.081	0.565	-0.623
sand	-0.543	-0.709	1.000	-0.760	-0.478	0.574
silt	0.156	0.081	-0.760	1.000	0.155	-0.238
ph	0.879	0.565	-0.478	0.155	1.000	-0.917
organic	-0.922	-0.623	0.574	-0.238	-0.917	1.000

Correlation table for soil variables for M. aprica

	bulk	clay	sand	silt	ph	organic
bulk	1.000	0.026	0.167	-0.314	0.852	-0.930
clay	0.026	1.000	-0.900	0.655	-0.121	-0.003
sand	0.167	-0.900	1.000	-0.918	0.285	-0.172
silt	-0.314	0.655	-0.918	1.000	-0.386	0.302
ph	0.852	-0.121	0.285	-0.386	1.000	-0.847
organic	-0.930	-0.003	-0.172	0.302	-0.847	1.000

Correlation table for soil variables for M. bryophora

	bulk	clay	sand	silt	ph	organic
bulk	1.000	-0.031	-0.338	-0.338	0.859	-0.936
clay	-0.031	1.000	0.707	0.707	-0.159	0.052
sand	-0.338	0.707	1.000	1.000	-0.427	0.348
silt	-0.338	0.707	1.000	1.000	-0.427	0.348
ph	0.859	-0.159	-0.427	-0.427	1.000	-0.849
organic	-0.936	0.052	0.348	0.348	-0.849	1.000

Correlation table for soil variables for M. calycina

	bulk	clay	sand	silt	ph	organic
bulk	1.000	0.048	0.044	-0.077	0.093	-0.320
clay	0.048	1.000	-0.428	-0.125	0.197	-0.119
sand	0.044	-0.428	1.000	-0.843	0.063	-0.264
silt	-0.077	-0.125	-0.843	1.000	-0.187	0.361
ph	0.093	0.197	0.063	-0.187	1.000	-0.121
organic	-0.320	-0.119	-0.264	0.361	-0.121	1.000

Correlation table for soil variables for M. eriophora

	bulk	clay	sand	silt	ph	organic
bulk	1.000	-0.336	0.565	-0.320	0.805	-0.746
clay	-0.336	1.000	-0.820	-0.418	-0.227	0.416
sand	0.565	-0.820	1.000	-0.177	0.405	-0.563
silt	-0.320	-0.418	-0.177	1.000	-0.254	0.178
ph	0.805	-0.227	0.405	-0.254	1.000	-0.712
organic	-0.746	0.416	-0.563	0.178	-0.712	1.000

Correlation table for soil variables for M. ferruginea

	bulk	clay	sand	silt	ph	organic
bulk	1.000	0.389	-0.105	-0.208	0.674	-0.790
clay	0.389	1.000	-0.654	-0.028	0.514	-0.416
sand	-0.105	-0.654	1.000	-0.738	-0.271	0.195
silt	-0.208	-0.028	-0.738	1.000	-0.101	0.113
ph	0.674	0.514	-0.271	-0.101	1.000	-0.741
organic	-0.790	-0.416	0.195	0.113	-0.741	1.000

Correlation table for soil variables for M. foliolosa

	bulk	clay	sand	silt	ph	organic
bulk	1.000	-0.027	0.107	-0.114	0.089	-0.367
clay	-0.027	1.000	-0.622	-0.053	0.315	0.072
sand	0.107	-0.622	1.000	-0.749	-0.117	-0.030
silt	-0.114	-0.053	-0.749	1.000	-0.117	-0.022
ph	0.089	0.315	-0.117	-0.117	1.000	-0.195
organic	-0.367	0.072	-0.030	-0.022	-0.195	1.000

Correlation table for soil variables for M. fusca

	bulk	clay	sand	silt	ph	organic
bulk	1.000	0.638	-0.436	-0.153	0.564	-0.475
clay	0.638	1.000	-0.729	-0.176	0.440	-0.481
sand	-0.436	-0.729	1.000	-0.545	-0.254	0.183
silt	-0.153	-0.176	-0.545	1.000	-0.173	0.326
ph	0.564	0.440	-0.254	-0.173	1.000	-0.368
organic	-0.475	-0.481	0.183	0.326	-0.368	1.000

Correlation table for soil variables for M. hieraciifolia

	bulk	clay	sand	silt	ph	organic
bulk	1.000	0.091	0.044	-0.127	0.195	-0.505
clay	0.091	1.000	-0.650	0.092	0.519	-0.085
sand	0.044	-0.650	1.000	-0.816	-0.203	-0.029
silt	-0.127	0.092	-0.816	1.000	-0.128	0.104
ph	0.195	0.519	-0.203	-0.128	1.000	-0.203
organic	-0.505	-0.085	-0.029	0.104	-0.203	1.000

Correlation table for soil variables for M. idahoensis

	bulk	clay	sand	silt	ph	organic
bulk	1.000	0.599	-0.425	0.020	0.845	-0.899
clay	0.599	1.000	-0.662	-0.029	0.555	-0.609
sand	-0.425	-0.662	1.000	-0.730	-0.406	0.448
silt	0.020	-0.029	-0.730	1.000	0.034	-0.041
ph	0.845	0.555	-0.406	0.034	1.000	-0.884
organic	-0.899	-0.609	0.448	-0.041	-0.884	1.000

Correlation table for soil variables for M. lyallii

	bulk	clay	sand	silt	ph	organic
bulk	1.000	0.218	-0.078	-0.085	0.626	-0.719
clay	0.218	1.000	-0.513	-0.218	0.521	-0.373
sand	-0.078	-0.513	1.000	-0.726	-0.184	0.104
silt	-0.085	-0.218	-0.726	1.000	-0.209	0.180
ph	0.626	0.521	-0.184	-0.209	1.000	-0.684
organic	-0.719	-0.373	0.104	0.180	-0.684	1.000

Correlation table for soil variables for M. melanocentra

	bulk	clay	sand	silt	ph	organic
bulk	1.000	0.166	0.026	-0.198	0.716	-0.661
clay	0.166	1.000	-0.751	0.163	-0.237	0.063
sand	0.026	-0.751	1.000	-0.774	0.177	-0.129
silt	-0.198	0.163	-0.774	1.000	-0.037	0.131
ph	0.716	-0.237	0.177	-0.037	1.000	-0.754
organic	-0.661	0.063	-0.129	0.131	-0.754	1.000

Correlation table for soil variables for M. micranthidifolia

	bulk	clay	sand	silt	ph	organic
bulk	1.000	0.122	0.226	-0.308	0.541	-0.802
clay	0.122	1.000	-0.626	0.393	0.492	-0.318
sand	0.226	-0.626	1.000	-0.963	-0.360	-0.055
silt	-0.308	0.393	-0.963	1.000	0.255	0.175
ph	0.541	0.492	-0.360	0.255	1.000	-0.487
organic	-0.802	-0.318	-0.055	0.175	-0.487	1.000

Correlation table for soil variables for M. nidifica

	bulk	clay	sand	silt	ph	organic
bulk	1.000	0.464	0.012	-0.356	0.852	-0.915
clay	0.464	1.000	-0.612	0.039	0.342	-0.448
sand	0.012	-0.612	1.000	-0.814	0.045	0.055
silt	-0.356	0.039	-0.814	1.000	-0.308	0.260
ph	0.852	0.342	0.045	-0.308	1.000	-0.877
organic	-0.915	-0.448	0.055	0.260	-0.877	1.000

Correlation table for soil variables for M. nivalis

	bulk	clay	sand	silt	ph	organic
bulk	1.000	0.229	-0.156	0.023	0.323	-0.625
clay	0.229	1.000	-0.670	0.087	0.570	-0.268
sand	-0.156	-0.670	1.000	-0.798	-0.289	0.122
silt	0.023	0.087	-0.798	1.000	-0.075	0.054
ph	0.323	0.570	-0.289	-0.075	1.000	-0.336
organic	-0.625	-0.268	0.122	0.054	-0.336	1.000

Correlation table for soil variables for M. nudicaulis

	bulk	clay	sand	silt	ph	organic
bulk	1.000	-0.079	0.458	-0.475	0.520	-0.487
clay	-0.079	1.000	-0.453	0.006	0.169	0.151
sand	0.458	-0.453	1.000	-0.893	0.355	-0.606
silt	-0.475	0.006	-0.893	1.000	-0.484	0.604
ph	0.520	0.169	0.355	-0.484	1.000	-0.427
organic	-0.487	0.151	-0.606	0.604	-0.427	1.000

Correlation table for soil variables for M. occidentalis

	bulk	clay	sand	silt	ph	organic
bulk	1.000	0.410	-0.251	-0.114	0.819	-0.876
clay	0.410	1.000	-0.716	-0.132	0.530	-0.509
sand	-0.251	-0.716	1.000	-0.598	-0.343	0.310
silt	-0.114	-0.132	-0.598	1.000	-0.121	0.144
ph	0.819	0.530	-0.343	-0.121	1.000	-0.880
organic	-0.876	-0.509	0.310	0.144	-0.880	1.000

Correlation table for soil variables for M. odontoloma

	bulk	clay	sand	silt	ph	organic
bulk	1.000	0.467	0.154	-0.511	0.878	-0.931
clay	0.467	1.000	-0.562	-0.015	0.373	-0.477
sand	0.154	-0.562	1.000	-0.819	0.190	-0.096
silt	-0.511	-0.015	-0.819	1.000	-0.489	0.447
ph	0.878	0.373	0.190	-0.489	1.000	-0.886
organic	-0.931	-0.477	-0.096	0.447	-0.886	1.000

Correlation table for soil variables for M. oregana

	bulk	clay	sand	silt	ph	organic
bulk	1.000	0.446	0.042	-0.367	0.873	-0.907
clay	0.446	1.000	-0.598	0.041	0.319	-0.426
sand	0.042	-0.598	1.000	-0.826	0.096	0.016
silt	-0.367	0.041	-0.826	1.000	-0.344	0.279
ph	0.873	0.319	0.096	-0.344	1.000	-0.866
organic	-0.907	-0.426	0.016	0.279	-0.866	1.000

Correlation table for soil variables for M. pallida

	bulk	clay	sand	silt	ph	organic
bulk	1.000	0.271	-0.104	-0.212	0.641	-0.656
clay	0.271	1.000	-0.857	0.129	-0.273	-0.183
sand	-0.104	-0.857	1.000	-0.621	0.247	0.010
silt	-0.212	0.129	-0.621	1.000	-0.059	0.260
ph	0.641	-0.273	0.247	-0.059	1.000	-0.499
organic	-0.656	-0.183	0.010	0.260	-0.499	1.000

Correlation table for soil variables for M. petiolaris

	bulk	clay	sand	silt	ph	organic
bulk	1.000	-0.022	0.371	-0.409	0.472	-0.799
clay	-0.022	1.000	-0.543	0.350	0.505	-0.071
sand	0.371	-0.543	1.000	-0.977	-0.326	-0.166
silt	-0.409	0.350	-0.977	1.000	0.234	0.203
ph	0.472	0.505	-0.326	0.234	1.000	-0.433
organic	-0.799	-0.071	-0.166	0.203	-0.433	1.000

Correlation table for soil variables for M. razshivinii

	bulk	clay	sand	silt	ph	organic
bulk	1.000	-0.050	0.110	-0.088	0.127	-0.329
clay	-0.050	1.000	-0.367	-0.181	0.193	-0.053
sand	0.110	-0.367	1.000	-0.848	-0.070	-0.378
silt	-0.088	-0.181	-0.848	1.000	-0.036	0.430
ph	0.127	0.193	-0.070	-0.036	1.000	0.026
organic	-0.329	-0.053	-0.378	0.430	0.026	1.000

Correlation table for soil variables for M. reflexa

	bulk	clay	sand	silt	ph	organic
bulk	1.000	0.134	0.038	-0.130	0.050	-0.336
clay	0.134	1.000	-0.431	-0.195	0.013	-0.138
sand	0.038	-0.431	1.000	-0.800	0.065	-0.280
silt	-0.130	-0.195	-0.800	1.000	-0.079	0.397
ph	0.050	0.013	0.065	-0.079	1.000	-0.038
organic	-0.336	-0.138	-0.280	0.397	-0.038	1.000

Correlation table for soil variables for M. rhomboidea

	bulk	clay	sand	silt	ph	organic
bulk	1.000	0.402	0.276	-0.587	0.887	-0.945
clay	0.402	1.000	-0.554	0.030	0.357	-0.463
sand	0.276	-0.554	1.000	-0.849	0.251	-0.223
silt	-0.587	0.030	-0.849	1.000	-0.529	0.563
ph	0.887	0.357	0.251	-0.529	1.000	-0.869
organic	-0.945	-0.463	-0.223	0.563	-0.869	1.000

Correlation table for soil variables for M. rufidula

	bulk	clay	sand	silt	ph	organic
bulk	1.000	0.466	-0.361	0.097	0.782	-0.856
clay	0.466	1.000	-0.704	0.109	0.247	-0.336
sand	-0.361	-0.704	1.000	-0.782	-0.278	0.372
silt	0.097	0.109	-0.782	1.000	0.173	-0.225
ph	0.782	0.247	-0.278	0.173	1.000	-0.871
organic	-0.856	-0.336	0.372	-0.225	-0.871	1.000

Correlation table for soil variables for M. spicata

	bulk	clay	sand	silt	ph	organic
bulk	1.000	-0.094	0.166	-0.126	0.365	-0.379
clay	-0.094	1.000	-0.367	-0.146	0.246	0.013
sand	0.166	-0.367	1.000	-0.866	-0.078	-0.300
silt	-0.126	-0.146	-0.866	1.000	-0.049	0.312
ph	0.365	0.246	-0.078	-0.049	1.000	-0.211
organic	-0.379	0.013	-0.300	0.312	-0.211	1.000

Correlation table for soil variables for M. stellaris

	bulk	clay	sand	silt	ph	organic
bulk	1.000	0.751	-0.573	0.144	0.848	-0.845
clay	0.751	1.000	-0.850	0.347	0.819	-0.662
sand	-0.573	-0.850	1.000	-0.789	-0.651	0.569
silt	0.144	0.347	-0.789	1.000	0.205	-0.241
ph	0.848	0.819	-0.651	0.205	1.000	-0.806
organic	-0.845	-0.662	0.569	-0.241	-0.806	1.000

Correlation table for soil variables for M. tolmiei

	bulk	clay	sand	silt	ph	organic
bulk	1.000	0.462	-0.011	-0.336	0.828	-0.894
clay	0.462	1.000	-0.646	0.086	0.408	-0.426
sand	-0.011	-0.646	1.000	-0.816	-0.004	0.059
silt	-0.336	0.086	-0.816	1.000	-0.304	0.246
ph	0.828	0.408	-0.004	-0.304	1.000	-0.860
organic	-0.894	-0.426	0.059	0.246	-0.860	1.000
