# Supplementary material for: The future of cold‐adapted plants in changing climates: Micranthes (Saxifragaceae) as a case study
Source: Ecol Evol. 2018 Jun 25;8(14):7164–77. doi: 10.1002/ece3.4242 (PMC6065370; doi:10.1002/ece3.4242)
Supplement: Supplementary file 4 [file ECE3-8-7164-s004.pdf]

Appendix S2. Comparisons of different suitability thresholds.

species	TTP	future/current	species	MTP	future/current	species	MSS	future/current
apetala	0.527	0.025	apetala	0.4249	0.042	apetala	0.5214	0.027
aprica	0.4499	0.185	aprica	0.0357	0.716	aprica	0.269	0.402
bryophora	0.4128	0.844	bryophora	0.0301	1.119	bryophora	0.2141	0.967
calycina	0.1945	0.210	calycina	0.0385	0.159	calycina	0.1805	0.203
eriophora	0.0804	0.462	eriophora	0.0804	0.462	eriophora	0.3923	0.483
ferruginea	0.2441	0.256	ferruginea	0.0120	0.765	ferruginea	0.2386	0.262
foliolosa	0.3644	1.905	foliolosa	0.0019	1.196	foliolosa	0.1998	1.901
fusca	0.1426	0.310	fusca	0.0729	0.401	fusca	0.1775	0.277
hieraciifolia	0.2042	3.564	hieraciifolia	0.0052	1.068	hieraciifolia	0.1733	1.347
idahoensis	0.4181	9.260	idahoensis	0.2059	2.885	idahoensis	0.4413	10.240
lyallii	0.3128	0.650	lyallii	0.0532	0.823	lyallii	0.395	0.537
melanocentra	0.2921	0.629	melanocentra	0.1251	0.925	melanocentra	0.4332	0.693
micranthidifolia	0.3779	1.574	micranthidifolia	0.1489	1.244	micranthidifolia	0.3998	1.613
nidifica	0.3012	1.607	nidifica	0.0368	1.044	nidifica	0.3142	1.638
nivalis	0.195	0.676	nivalis	0.0067	0.261	nivalis	0.1778	0.659
nudicaulis	0.173	0.206	nudicaulis	0.1381	0.292	nudicaulis	0.1988	0.160
occidentalis	0.3031	5.173	occidentalis	0.0397	1.168	occidentalis	0.359	6.212
odontoloma	0.3165	1.066	odontoloma	0.0162	0.986	odontoloma	0.2843	1.056
oregana	0.4129	0.442	oregana	0.0344	0.651	oregana	0.3876	0.456
pallida	0.2581	0.744	pallida	0.0918	0.873	pallida	0.3764	0.806
petiolaris	0.4662	0.282	petiolaris	0.1216	0.287	petiolaris	0.4144	0.317
razshivinii	0.3549	0.255	razshivinii	0.1365	0.441	razshivinii	0.4179	0.247
reflexa	0.221	0.395	reflexa	0.1557	0.475	reflexa	0.3735	0.470
rhomboidea	0.4559	0.398	rhomboidea	0.0630	1.228	rhomboidea	0.3657	0.655
rufidula	0.2061	1.925	rufidula	0.0480	1.404	rufidula	0.3786	3.002
spicata	0.2379	0.258	spicata	0.0790	0.610	spicata	0.4468	0.237
stellaris	0.2774	0.625	stellaris	0.0109	0.968	stellaris	0.2458	0.628
tenuis	0.1906	0.518	tenuis	0.0122	0.545	tenuis	0.1637	0.536
tolmiei	0.3602	0.271	tolmiei	0.0276	0.272	tolmiei	0.313	0.039

10% Training Presence (TTP), Minimum Training Presence (MTP), and the Maximum training Sensitivity Plus Specificity (MSS).
Future/current equals total future area divided by total current area; number > 1 equals increase in habitat under climate change.
Species with numbers >1 are in red
