# Supplementary material for: The future of cold‐adapted plants in changing climates: Micranthes (Saxifragaceae) as a case study
Source: Ecol Evol. 2018 Jun 25;8(14):7164–77. doi: 10.1002/ece3.4242 (PMC6065370; doi:10.1002/ece3.4242)
Supplement: Supplementary file 5 [file ECE3-8-7164-s005.pdf]

Appendix S3. Correlation matrix of variables used in PCA.

	Latitude	Elevation	Mean Annual Temperature	Annual Precipitation	Soil pH	Silt
Latitude	1	-0.73	-0.6	-0.25	0.03	0.3
Elevation	-0.73	1	-0.01	0.01	0.12	-0.23
Mean Annual Temperature	-0.6	-0.01	1	0.49	-0.24	-0.28
Annual Precipitation	-0.25	0.01	0.49	1	-0.41	-0.34
Soil pH	0.03	0.12	-0.24	-0.41	1	-0.01
Silt	0.3	-0.23	-0.28	-0.34	-0.01	1
