# Supplementary material for: The future of cold‐adapted plants in changing climates: Micranthes (Saxifragaceae) as a case study
Source: Ecol Evol. 2018 Jun 25;8(14):7164–77. doi: 10.1002/ece3.4242 (PMC6065370; doi:10.1002/ece3.4242)
Supplement: Supplementary file 6 [file ECE3-8-7164-s006.pdf]

Appendix S4. Results from Ecological Niche Models (ENMs) for dataset not corrected for sampling bias.

	**Dataset with sampling bias not corrected**				
Taxon	Schoener's *D*	Current Niche (km2)	Future Niche (km2)	Overlap (km2)	% Change
M. apetala	0.785	29572	2433	2433	-91.77
M. aprica	0.687	19843	2253	1910	-88.65
M. bryophora	0.802	13859	18496	12758	+33.46
M. calycina	0.320	170988	19418	14298	-88.64
M. eriophora	0.904	135300	66261	66261	-51.03
M. ferruginea	0.827	232976	130994	124197	-43.77
M. foliolosa	0.474	239995	464630	96992	+93.6
M. fusca	0.776	23512	12294	12079	-47.71
M. hieraciifolia	0.527	766210	695180	312154	-9.27
M. idahoensis	0.632	51914	381705	48514	+635.26
M. lyallii	0.675	387606	421273	221070	+8.69
M. melanocentr	0.917	895071	848994	768149	-5.15
M. micranthidifc	0.738	56788	72426	42139	+27.54
M. nidifica	0.742	168048	213322	120112	+26.94
M. nivalis	0.609	347056	273843	221374	-21.1
M. nudicaulis	0.582	8998	195	130	-97.83
M. occidentalis	0.634	139630	501351	112976	+259.06
M. odontoloma	0.846	210351	273359	192982	+29.95
M. oregana	0.736	150944	141151	97530	-6.49
M. pallida	0.866	374905	318697	268603	-14.99
M. petiolaris	0.721	3527	308	239	-91.27
M. razshivinii	0.833	178740	124985	113207	-30.07
M. reflexa	0.688	456031	208090	124470	-54.37
M. rhomboidea	0.663	53963	45675	31153	-15.36
M. rufidula	0.688	82259	208907	79623	+153.96
M. spicata	0.724	413497	236462	168467	-42.81
M. stellaris	0.772	502937	303223	281727	-39.71
M. tenuis	0.509	187197	94649	69323	-49.44
M. tolmiei	0.675	37666	9432	8800	-74.96
