# Supplementary material for: The future of cold‐adapted plants in changing climates: Micranthes (Saxifragaceae) as a case study
Source: Ecol Evol. 2018 Jun 25;8(14):7164–77. doi: 10.1002/ece3.4242 (PMC6065370; doi:10.1002/ece3.4242)
Supplement: Supplementary file 7 [file ECE3-8-7164-s007.pdf]

Appendix S5. Results from each Principle Component Analysis for each subset of data.

All Species			
	PC1	PC2	PC3
Latitude	0.5472456	-0.33396711	0.2749086
Elevation	-0.3316509	0.59369203	-0.274036
Annual Mean Temperature	-0.4890342	-0.24128802	-0.0159184
Annual Precipitation	-0.4199879	-0.42565812	0.1276262
Soil pH	0.1869577	0.54235175	0.4922778
Silt Content	0.3742091	-0.04945281	-0.7684076
Importance of components:			
	PC1	PC2	PC3
Standard	1.5456	1.2426	0.9338
Proportion	0.3981	0.2574	0.1453
Cumlative	0.3981	0.6555	0.8008

Mountain Species			
	PC1	PC2	PC3
Latitude	-0.44781364	-0.20847419	0.4940061
Elevation	0.63930402	-0.04836202	0.1951796
Annual Mean Temperature	-0.40997605	0.30592513	-0.5955495
Annual Precipitation	-0.20132227	-0.59707742	-0.3305732
Soil pH	-0.05397508	0.70858932	0.1060732
Silt Content	-0.42335446	0.04482389	0.4925987
Importance of components:			
	PC1	PC2	PC3
Standard	1.4908	1.259	1.1336
Proportion	0.3704	0.2642	0.2142
Cumlative	0.3704	0.6346	0.8488

Arctic and Mountain/Artic Species			
	PC1	PC2	PC3
Latitude	0.5048068	-0.3659555	0.05888311
Elevation	-0.1708139	0.81022348	0.19781939
Annual Mean Temperature	-0.4966348	-0.31630794	-0.17187308
Annual Precipitation	-0.509271	-0.1003055	0.18080294
Soil pH	0.3557798	0.07426171	0.61438732
Silt Content	0.2888085	0.30657428	-0.71951263
Importance of components:			
	PC1	PC2	PC3
Standard	1.6388	1.1358	1.006
Proportion	0.4476	0.215	0.1687
Cumlative	0.4476	0.6626	0.8313

Narrowly Endemic Species			
	PC1	PC2	PC3
Latitude	-0.281946	0.5100043	-0.4795136
Elevation	0.5993291	-0.1680946	-0.171626
Annual Mean Temperature	-0.4242911	-0.1005454	0.668392
Annual Precipitation	-0.3931828	-0.5310327	-0.1456614
Soil pH	0.1649538	0.6024159	0.447503
Silt Content	-0.4466382	0.2379703	-0.2690486
Importance of components:			
	PC1	PC2	PC3
Standard	1.5481	1.246	1.0929
Proportion	0.3994	0.2588	0.1991
Cumlative	0.3994	0.6582	0.8573
