# Supplementary material for: The future of cold‐adapted plants in changing climates: Micranthes (Saxifragaceae) as a case study
Source: Ecol Evol. 2018 Jun 25;8(14):7164–77. doi: 10.1002/ece3.4242 (PMC6065370; doi:10.1002/ece3.4242)
Supplement: Supplementary file 8 [file ECE3-8-7164-s008.docx]

Appendix S1. Correlation tables for all *Micranthes* species.

Appendix S2. Comparison of different suitability thresholds.

Appendix S3. Correlation matrix for variables included in PCA and MANOVA analyses.

Appendix S4. Results from Ecological Niche Models (ENMs) for dataset not corrected for sampling bias.

Appendix S5. Results from each Principle Component Analysis for each subset of data.

Figure S1. Ecological niche models for all *Micranthes* species in analysis. All models corrected for sampling models. From left to right, in the first panel green areas represent the original shapefiles that were used to trim layers. They represent all current and potential habitats. Yellow dots are accessions used to train and test the models. In the second panel, purple areas are designating current geographical area. In the third panel, orange areas are designating future predicted geographical area. In the second and third panels, darker shades represent more suitable. Suitable areas designated by the TTP threshold. All areas are in the Northern Hemisphere. Letters correspond to species listed in Table 1 and images are alphabetical, except for species with larger distributions.

Figure S2. Plot of principal component 1 versus principal component 2 for the PCA performed on all *Micranthes* species. All species distinguished by color and shape. Statistically significant separation among species that gain and lose habitat occurs along both PCA axis (overall: F_1,24723_ = 1726.7, P < 0.001; x-axis: F_1,24723_ = 960.8, P < 0.001; y-axis F_1,24723_ = 2301.5, P < 0.001). The x-axis explains 39.8% of the variation and the y-axis explains 25.7%.
